# Adapalene Inhibits Prostate Cancer Cell Proliferation *In Vitro* and *In Vivo* by Inducing DNA Damage, S-phase Cell Cycle Arrest, and Apoptosis

**DOI:** 10.3389/fphar.2022.801624

**Published:** 2022-02-22

**Authors:** Hai-bin Nong, Ya-nan Zhang, Yi-guang Bai, Qiong Zhang, Ming-fu Liu, Quan Zhou, Zhuo-hua Shi, Gao-feng Zeng, Shao-Hui Zong

**Affiliations:** ^1^ Department of Spine Osteopathia, The First Affiliated Hospital of Guangxi Medical University, Guangxi Medical University, Nanning, China; ^2^ Collaborative Innovation Center of Guangxi Biological Medicine, Guangxi Medical University, Nanning, China; ^3^ Department of Orthopaedics, Nanchong Central Hospital, The Second Clinical Institute of North Sichuan Medical College, Nanchong, China; ^4^ Department of Nutrition and Food Hygiene, College of Public Hygiene of Guangxi Medical University, Nanning, China; ^5^ Research Centre for Regenerative Medicine and Guangxi Key Laboratory of Regenerative Medicine, Guangxi Medical University, Nanning, China

**Keywords:** adapalene, prostate cancer, DNA damage, cell cycle, apoptosis

## Abstract

**Aims:** Prostate cancer is a well-known aggressive malignant tumor in men with a high metastasis rate and poor prognosis. Adapalene (ADA) is a third-generation synthetic retinoid with anticancer properties. We investigated the anti-tumor activity and molecular mechanisms of ADA in the RM-1 prostate cancer cell line *in vivo* and *in vitro*.

**Methods:** The effects of ADA on cell proliferation were estimated using the CCK-8 and colony formation assays. The wound-healing assay and the Transwell assay were employed to examine the migratory capacity and invasiveness of the cells. Flow cytometry was utilized to evaluate the cell cycle and apoptosis, and Western blotting analysis was used to assess the expression of the associated proteins. Micro-CT, histomorphological, and immunohistochemical staining were used to assess the effects of ADA on bone tissue structure and tumor growth in a mouse model of prostate cancer bone metastasis.

**Result:** ADA dramatically inhibited cell proliferation, migration, invasiveness, and induced S-phase arrest and apoptosis. ADA also regulated the expression of S-phase associated proteins and elevated the levels of DNA damage markers, p53, and p21 after ADA treatment, suggesting that the anti-tumor effect of ADA manifests through the DNA damage/p53 pathway. Furthermore, we observed that ADA could effectively inhibited tumor growth and bone destruction in mice.

**Conclusion:** ADA inhibited prostate cancer cell proliferation, elicited apoptosis, and arrested the cell cycle in the S-phase. ADA also slowed the rate of tumor growth and bone destruction *in vitro*. Overall, our results suggest that ADA may be a potential treatment against prostate cancer.

## Introduction

Approximately 1.41 million new cases and 37.5 million deaths worldwide are estimated to be caused by prostate cancer, which was the second most frequent cancer in 2020 and the fifth leading cause of cancer-related deaths (Sung et al., 2021). Furthermore, 85–100% of patients who died from prostate cancer presented bone metastasis (Carlin and Andriole, 2000). Prostate cancer is often androgen-dependent and initially responds to hormone therapy (Tang and Porter, 1997). Currently, the standard treatments for prostate cancer include hormone therapy, chemotherapy and radiotherapy (Cornford et al., 2017). Unfortunately, these treatments are eventually difficult to avoid androgen resistance and tumor metastasis (Gao et al., 2015). Moreover, these forms of treatment not only cannot inhibit tumor development and metastasis, but also are highly toxic and drug resistance in normal tissues (Li et al., 2015). Therefore, the search for safe, effective, and reliable treatments, especially those targeting androgens remains a hot topic in the field of prostate cancer research.

Adapalene, a synthetic derivative of retinoic acid, is a topical retinoid that is often used clinically to treat various skin diseases (Rusu et al., 2020). The pharmacological effects of ADA including comedolytic activity, anti-inflammatory activity, and anticancer activity have been studied widely (Millikan, 2010). In the field of anticancer research, one study found that ADA exhibited greater anticancer effects through inhibit CDK2 in colorectal cell lines (Shi et al., 2015). Retinoic acid as an anticancer drug exerts differentiation-promoting activity, which may be associated with intrinsic cytotoxic and pro-apoptotic effects (Simoni and Tolomeo, 2001). As recently reported, ADA inhibited the growth of ovarian cancer ES-2 cells by targeting glutamicoxaloacetic transaminase 1 (GOT1) and induced apoptosis by regulating the Bax/Bcl-2 ratio in hepatoma cells (Ocker et al., 2004; Wang et al., 2019). ADA triggered cell cycle arrest in the G1 phase of colorectal cancer cells and inhibited the proliferation of melanoma cells through the arrest of the cell cycle in the S phase, and then inhibited apoptosis by inducing DNA damage (Ocker et al., 2003; Li et al., 2019). However, to date, there are no reports on the use of ADA in the treatment of prostate cancer. Hence, we aimed to investigate the anticancer effect of ADA in the prostate cancer cell and provide a novel strategy for prostate cancer treatment.

## Materials and Methods

### Chemicals and Antibodies

Adpalene (ADA, 106685-40-9) was purchased from AbMole Bioscience (Shanghai, China), Antibodies Cyclin-B1, CyclinD2, CDK2, Rb, ATM, phosphorylated histone variant H2A.X at serine139 (γ-H2A.X), p-CDK2 (Thr-160), and β-actin were procured from CST (Cell Signaling Technology, Beverly, MA, United States). Bax, Bcl-2, p53, p21 Waf1/Cip1(p21), Cyclin-A2, Cyclin-E1, phosphorylated (p)-CDK2, p-Rb(Ser-795), and the secondary antibody(goat anti-rabbit horseradish peroxidase-conjugated IgG) were acquired from Beyotime Biotechnology (Beijing, China).

### Cell Lines and Cell Culture

The RM-1 cell line was purchased from the Shanghai Biochemical Cell Institute of the Chinese Academy of Sciences. RM-1 cells were cultured in DMEM (Gibco, United States) containing 10% FBS (VWR, Australia), 100 U/ml penicillin, and 100 pg/ml streptomycin (Gibco) at a temperature of 37°C, 5% CO_2_ concentration, and 95% humidified air. Cells were passaged every 48–72 h using 0.25% trypsin containing 0.02% EDTA (Solarbio, Beijing, China).

### Proliferation-Cytotoxicity Assay

RM-1 cells were collected and suspended in DMEM at a concentration of 2 × 10^4^ cells/ml. The suspended cells were subsequently seeded overnight in 96-well plates with each well containing 100 μl medium and treated with increasing ADA dosages (0–40 µM) and then incubated for 24 h or 48 h in a humidified incubator at 37°C and 5% CO_2._ Next, 100 μl fresh DMEM containing 10% CCK-8 reagent (Dojindo, Japan) was added to each well and the cells were incubated at37 °C in the dark for 30 min. The absorbance of each well was measured using a microplate reader (Gen5; BioTek, United States) at a wavelength of 450 nm.

### Colony Formation Assay

RM-1 cells were collected and suspended at a concentration of 100 cells/ml. Then, the suspended cells were seeded in 6-well culture plates at a volume of 2 ml per well overnight and treated with (0, 1.25, 2.5, and 5 µM) ADA for 24 h. The cells were cultured for an additional week after the replacement of fresh DMEM. Next, the cells were fixed using 4% paraformaldehyde for 20 min and stained using 0.5% crystal violet solution at room temperature for 10 min. Colonies containing more than 50 cells were pictured and then quantified using the Image-J software.

### Wound-Healing Assay

RM-1 cells were collected and suspended in DMEM at a concentration of 3 × 10^5^ cells/ml. Subsequently, the suspended cells were plated in 6-well culture plates in a volume of 2 ml per well and placed in an incubator until the cell density was 90% or more. A sterile 100-µl plastic pipette tip was used to create a wound. The cell debris was cleaned by washing with PBS and the wounds were imaged using an inverted light microscope (Olympus Corporation) with a digital camera (magnification 40X) at 0 h. Each well was added serum-free DMEM containing different concentrations of ADA and were incubated for 12 or 24 h. After washing the cells with PBS, 10 fields were randomly photographed using an inverted light microscope. The areas of wound healing were analyzed using the Image-J software.

### Cell Invasion Experiments

Cell invasiveness was detected using an 8-µm Transwell assay. Following treatment with different concentrations of ADA for 24 h, the cells were collected and then resuspended with serum-free DMEM, and adjusted to a concentration of 5 × 10^5^ cells/ml. The upper chamber was covered with the BD MatrigelTM matrix as per the protocols instructions. The Transwell inserts were plated in a 24-well plate, and 600 µl of DMEM containing 20% FBS was added to the lower chamber, a volume of 100 µl of the cell suspension was introduced into the upper chamber. The Transwell plate was placed in an incubator for 24 h, after which, the medium was aspirated and a cotton swab was used to gently wipe the cells on the upper surface of the chamber. The cells in lower chamber were subsequently fixed with 4% paraformaldehyde for 20 min at ambient temperature, and then stained using 0.5% crystal violet solution for 10 min. The cells were then washed thrice using PBS to remove the unbound crystal violet. After drying, six visual fields were randomly selected, imaged, and the cells were quantified using an inverted light microscope.

### Apoptosis Assay

Trypsin without EDTA was used to digest RM-1 cells, which were then suspended at a concentration of 2 × 10^5^ cells/ml. The suspended cells were plated overnight into 6-well culture plates (2 ml per well) and treated with the indicated doses of ADA for 24 or 48 h, and cell morphology was observed. Next, apoptosis was assessed using an Annexin V-APC Apoptosis Detection Kit (Invitrogen, CA, United States); the cells were collected, washed once in pre-cooled PBS, then once in 1X binding buffer, resuspended, and incubated with Annexin V-APC working solution for 15 min at ambient temperature. The cells were then washed once in 1X binding buffer, resuspended, and incubated with propidium iodide (PI) working solution at ambient temperature for 10 min. After incubation, the cells were stored at 2–8°C in the dark and subjected to flow cytometry (Beckman Coulter, United States) within 4 h.

### Cell Cycle Analysis

The cell cycle was analyzed using a Cell Cycle Analysis Kit (Beyotime Biotechnology, Beijing, China). Trypsin without EDTA was used to digest the cells and suspend cells at the concentration of 2 × 10^5^ cells/ml. The suspended cells were plated into 6-well culture plates at a volume of 2 ml per well overnight and then treated using the specified doses of ADA for 12 or 24 h. The cells were then harvested and fixed in 70% ice-cold ethanol at a temperature of 4°C overnight. The cells were then washed using PBS and incubated with PI staining solution for 30 min at ambient temperature. Subsequently, the cells were subjected to flow cytometry. The data obtained were evaluated using the FlowJo V10 software to determine the cell cycle progression.

### Western Blotting

After treatment with ADA for 24 h, RM-1 cells were collected and washed thrice with ice-cold PBS, and subsequently lysed using RIPA lysis buffer on ice for 30 min. The total protein concentration was then determined using the BCA assay kit (Beyotime, China). Subsequently, 60 µg of protein was separated on an 8–16% SDS-PAGE separation system at 100V. Proteins were then transferred to a 0.22 μm PVDF membrane (Millipore, Billerica, MA, United States). The PVDF membranes were blocked using TBST comprising 5% BSA at ambient temperature for 1 h and incubated with primary antibodies at 4°C overnight. Each antibody was diluted as per manufacturer instructions. The PVDF membranes were washed three times using TBST, 5 min each, and incubated with secondary antibodies for 1 h at ambient temperature. Finally, the intensity of the immunoreactive bands was detected using the GE Amersham Imager 600 (General Electric, Boston, MA, United States). The data were analyzed using the Image J and SPSS software.

### Animals and Experimental Groups

All mice (C57BL/6; Female; weighing 20–25 g; 8 weeks old) were procured and maintained at the Laboratory Animal Center of Guangxi Medical University. The mice were reared in specified pathogen-free environments and subjected to light/dark cycles of 12 h at 50–60% humidity at 22–26°C. The environment was disinfected and nursed as per the standards of the Experimental Animal Ethics Committee of Guangxi Medical University (China). To construct the bone metastasis model, RM-1 cells were collected, and resuspended in serum and adjusted to a concentration of 2 × 10^7^ cells/mL. The mice were weighed and anesthetized intraperitoneally using 5% chloral hydrate at a dose of 400 mg/kg. Subsequently, the skin of the left knee was prepared and disinfected, at the anterior slope of the tibial plateau. A 1 ml syringe needle was used to rotate into the medullary cavity along the long axis of the tibia and long axis of the tibia and withdrawn. A 10 μl volume of RM-1 cells was injected slowly into the medullary cavity formed using a 100 μl microsyringe. After one week, if the volume of the tumor increased to about 80–100 mm^3^, the mice were randomly divided into groups (5 mice/group). Subsequently, the mice received daily treatment with 0.5% carboxymethylcellulose (CMC)-NaCl (Yuanye Biotechnology, China) containing varying doses of ADA (15, 30, and 60 mg/kg) for 14 days Shi et al., 2015. The tumor growth was monitored every 3 days. Subsequently, the mice were anesthetized and sacrificed by cervical dislocation; the left leg tumors were removed, weighed, measured, and scanned using a micro-CT scanner, and subjected to histomorphological and immunohistochemical staining, and images were captured and analyzed using the Image J software. The tumor volume was computed using the equation $\text{V}=\text{a}b^{2}/2$ (a = longest axis; b = shortest axis).

### Osteolytic Lesions and Architecture Assay

Osteolytic lesions and architecture were determined using the μCT system (SkyScan1072; Skyscan, Aartselaar, Belgium). Three-dimensional reconstructions were constructed and evaluated using the Skyscan NRecon and CTAn software (Bruker). For tibial specimens, a square region of interest was delineated near the bone growth plate for quantitative and qualitative analysis. For each tibia specimen, the morphometric bone parameters of trabecular spacing (Tb.Sp, mm), trabecular number (Tb.N, 1/mm), trabecular thickness (Tb.Th, mm), and bone volume to tissue volume (BV/TV, %) were measured.

### Histomorphology and Immunohistochemical Analysis

The excised tumors were fixed in 4% paraformaldehyde, neutral polyformaldehyde, paraffin-embedded, dewaxed, sliced and subsequently stained using hematoxylin and eosin (H&E). The Ki-67 Immunohistochemistry Detection System Kit (Yaji Biological, Shanghai, China) was used to determine tumor cell proliferation in tissues. Tumor segments were subjected to incubation using primary antibody against Ki-67 at 4°C overnight. Subsequently, the tumor segments were incubated in the presence of horseradish peroxidase-conjugated secondary antibody for 30 min at ambient temperature and visualized using DAB Horseradish Peroxidase Chromogenic kit. Images were captured using a Zeiss microscope.

### Tumor Tissue Apoptosis Analysis

To detect whether ADA induced tumor apoptosis, TUNEL staining was employed. The tumor segments were stained and incubated to allow the TUNEL reaction as per the instructions provided for the FITC-TUNEL cell apoptosis detection kit (Wuhan Servicebio Technology, Hubei, China). With the aid of an upright fluorescent microscope (Olympus BX53, Tokyo, Japan), the TUNEL-stained segments were examined and photographed. TUNEL-positive cells were quantified as a percentage of all cells in tumor tissue specimens from different cohorts.

### Statistical Analysis

All data were analyzed the SPSS software (v22.0; IBM Corp) and are expressed as the mean ± SD of three replicates experiments. One-way analysis of variance was used to demonstrate the differences among the groups. Histograms were drawn and analyzed using the GraphPadPrism 7.0 software. *p*-value < 0.05 was considered statistically significant.

## Result

### Adapalene Inhibited the Proliferation of RM-1cells

**FIGURE 1:**
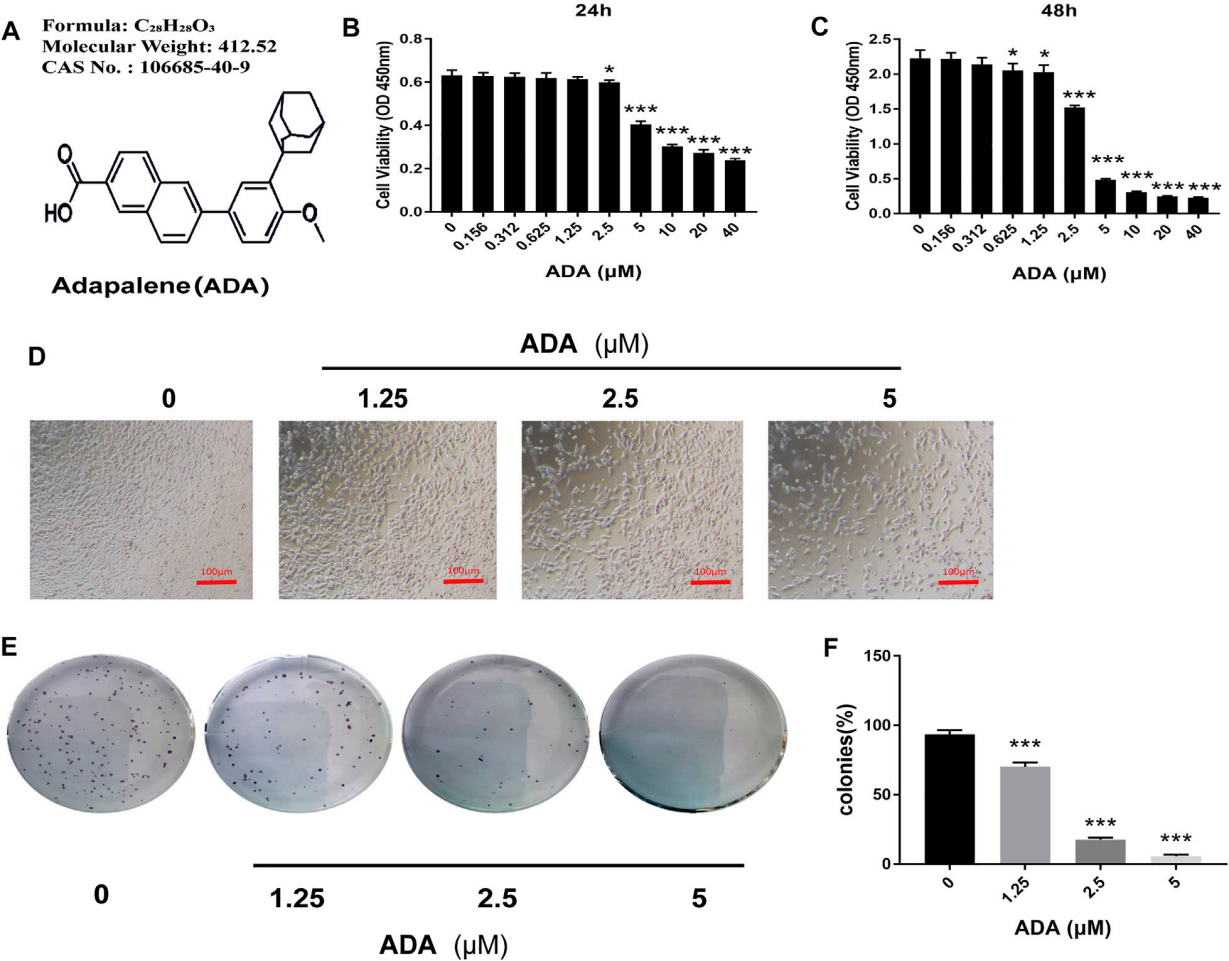
ADA significantly suppressed the proliferation of RM-1 cells. **(A)** Molecular structure of ADA. **(B,C)** CCK8 assays were used to detect the effects of varying ADA concentrations (0–40 μM) on the proliferation of RM-1 cells and evaluated by CCK8 assays. **(D)** Following treatment of ADA for 24 h, the morphology of RM-1 cells was visualized and captured utilizing an inverted microscope (100×). **(E)** The long-term effects of ADA in RM-1 cells were detected by colony formation assays and the colonies were stained by 0.5% crystal violet solution and manually counted. **(F)** ADA significantly inhibited colony formation in a dosage-dependent way. Data are shown as mean ± standard deviation. (*p < 0.05; **p < 0.01; ***p < 0.001 vs. control)

To evaluate the anticancer properties of ADA in prostate cancer, RM-1 cells were treated with(0, 0.156, 0.312, 0.625, 1.25, 2.5, 5, 10, 20, and 40 μM) ADA for 24 and 48 h. CCK-8 assay of the treated cells revealed that ADA suppressed the proliferation and viability of RM-1 cells and that the inhibitory effect was proportional to the treatment time and dose (Figures 1A–C). The half-maximal inhibitory concentration (IC50) of ADA was approximately 8.23 μM for RM-1 cells at 24 h whereas 3.08 μM at 48 h. Furthermore, RM-1 cells showed dramatic morphological changes after treatment with ADA after 24 h—cell proliferation was inhibited and the number of dead cells was increased in a dose-dependent manner (Figure 1D). Colony formation assays were performed to further assess if ADA showed anti-proliferative effects on RM-1 cells in the long term. We found that ADA significantly inhibited the colony-forming ability of RM-1 cells in a dose-dependent manner (Figures 1E,F). These findings indicate that ADA has an anti-proliferation effect on RM-1 cells.

### Adapalene Suppressed the Migration and Invasion of RM-1 Cells

**FIGURE 2:**
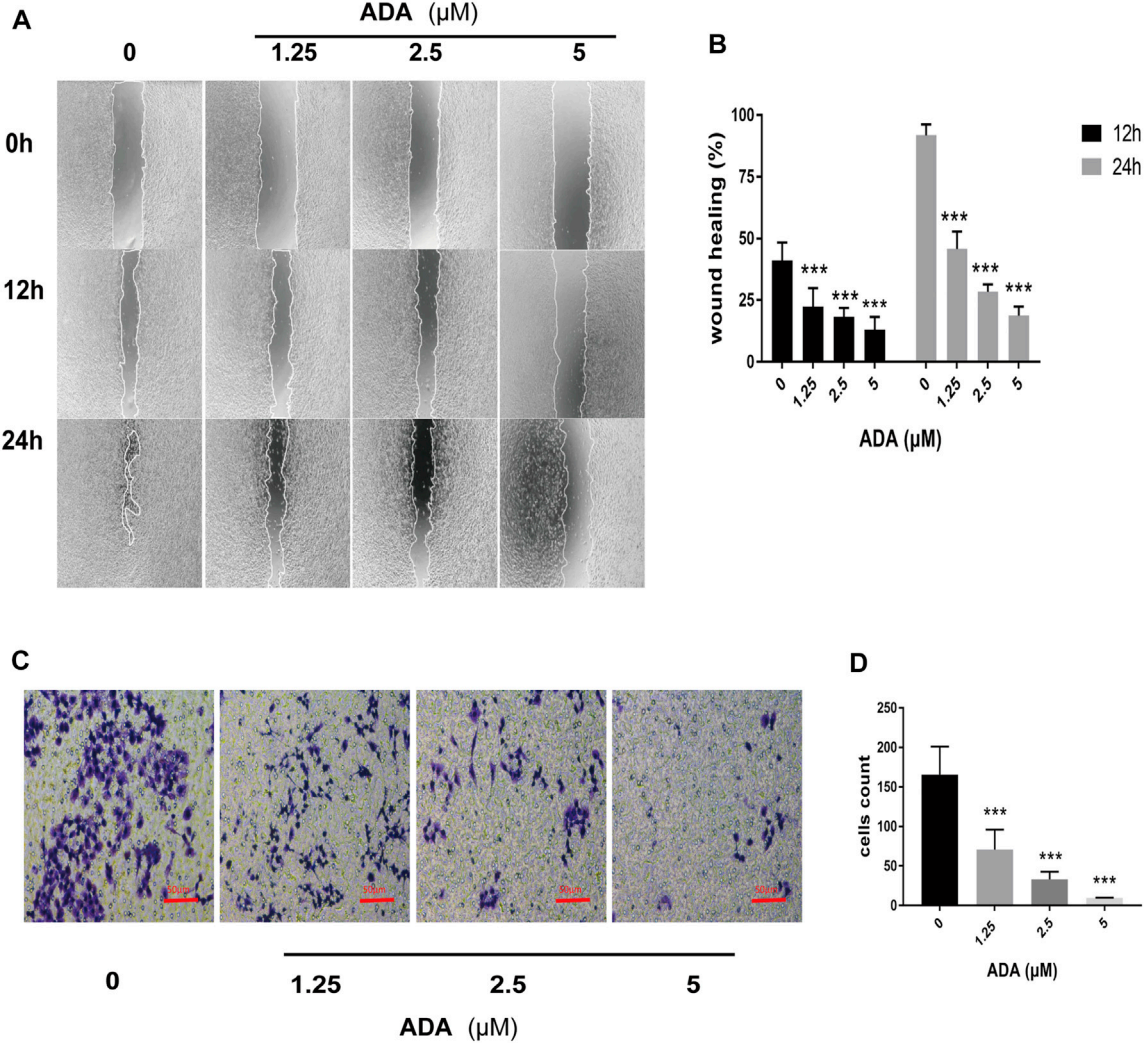
ADA suppressed the migration and invasion of RM-1 cells. **(A,B)** The healing effect and migration of RM-1 cells was determined using the scratch test. After treatment with varying concentrations of ADA for 12 or 24 h, the degree of wound healing of the cells was measured (40×). **(C,D)** The ratio of invasion of RM-1 cells was further determined using Transwell assay. After treating cells with varying ADA concentrations for 24 h, the proportion of invaded cells was stained and calculated (200×), Data are articulated as mean ± standard deviation. (*p < 0.05, **p < 0.01,***p < 0.001 vs. control).

The wound healing assay was used to determine the migration of RM-1 cells. We found that ADA significantly suppressed the migration ability of RM-1 cells and delayed wound healing time and ratio of acreage in a dose-dependent manner (Figures 2A,B). Furthermore, a Transwell assay was performed to determine the invasiveness of RM-1 cells. We found that ADA at high doses decreased the invasiveness of RM-1 cells (Figures 2C,D). Taken together, these results indicated that ADA prevented further RM-1 cells migration and invasion.

### Adapalene Induced Dramatic S Phase Arrest and Affected the S Phase-Related Proteins in RM-1 Cells

**FIGURE 3:**
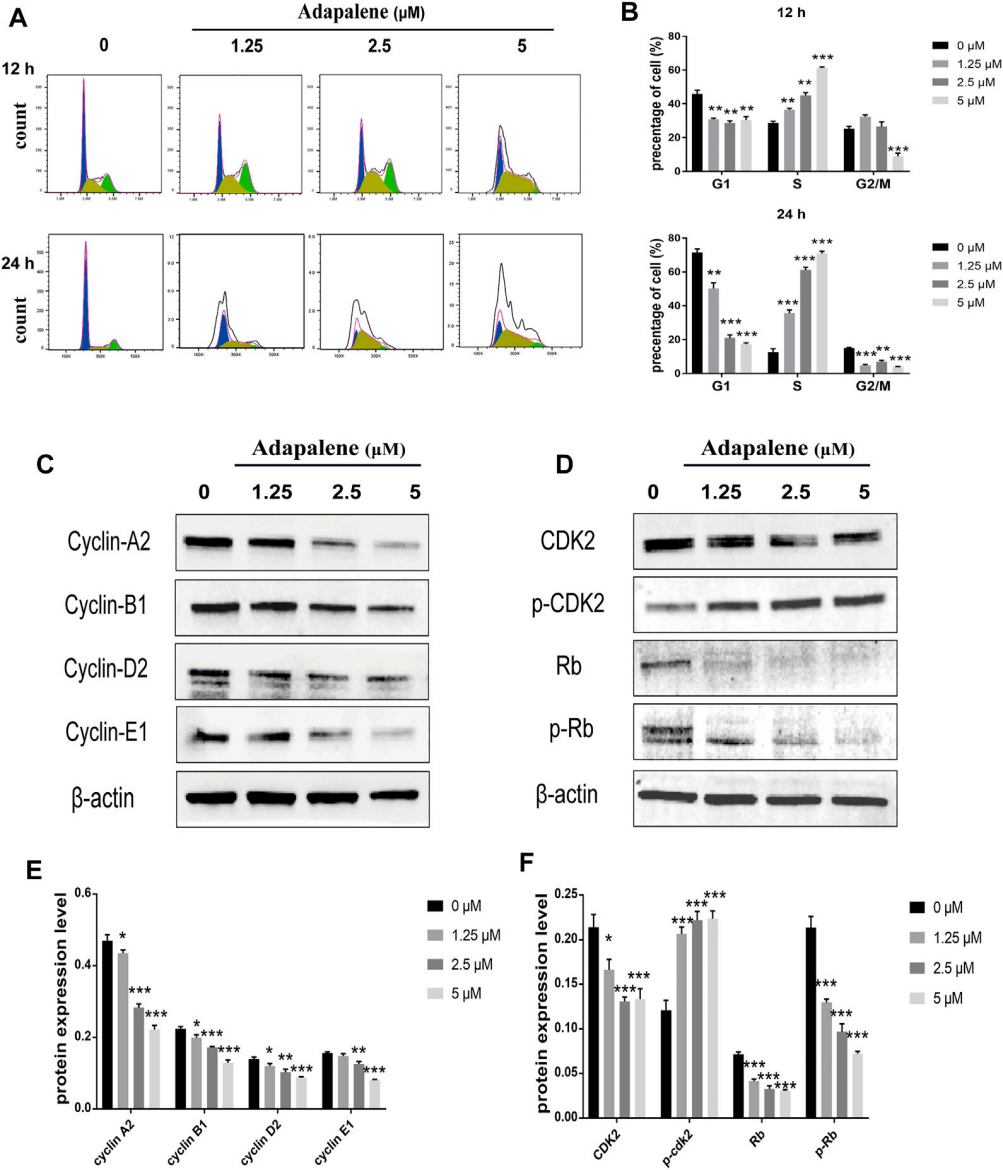
Impacts of ADA on cell cycle distribution and expression of related proteins in RM-1 cells. RM-1 cells were treated using 1.25, 2.5, or 5 μM ADA for 12 or 24 h. **(A)** The proportion of cells in each cell cycle phase was evaluated utilizing flow cytometry. **(B)** Histograms showed quantitative data on the cell cycle distribution. **(C,D)** The protein expression of CDK2, P-CDK2, Cyclin A2, Cyclin B1, Cyclin D2, Cyclin E1, Rb, and P-Rb were detected by western blotting and **(E,F)** Histograms illustrated the expression of each protein, and β-actin acted as the loading control. The samples derive from the same experiment and that blots were processed in parallel. Data are articulated as mean ± standard deviation. (*p < 0.05; **p < 0.01; ***p < 0.001 vs. control).

After treatment with ADA (0, 1.25, 2.5, and 5 μM) for 12 or 24 h, the percentage of cells in the S phase increased from 28.7% to 36.7%, 44.9%, and 61.2% in RM-1 cells after 12 h, respectively, and the percentage of cells in S phase increased from 12.6% to 35.5%, 61.1%, and 70.9% after 24 h, respectively (Figures 3A,B). associated proteins perform crucial functions in cell cycle progression in many tumors. Thus, to further explore the effects of ADA on the cell cycle, we assessed the expressions of cell cycle-related proteins in RM-1 cells by Western blotting. The results illustrated that ADA treatment considerably suppressed the expression of CDK2, Cyclin A2, Cyclin B1, Cyclin D2, Cyclin E1, Rb, and p-Rb in RM-1 cells at 24 h (Figures 3C–F), leading to S phase arrest in RM-1 cells.

### Adapalene Induces Apoptosis in RM-1 Cells

**FIGURE 4:**
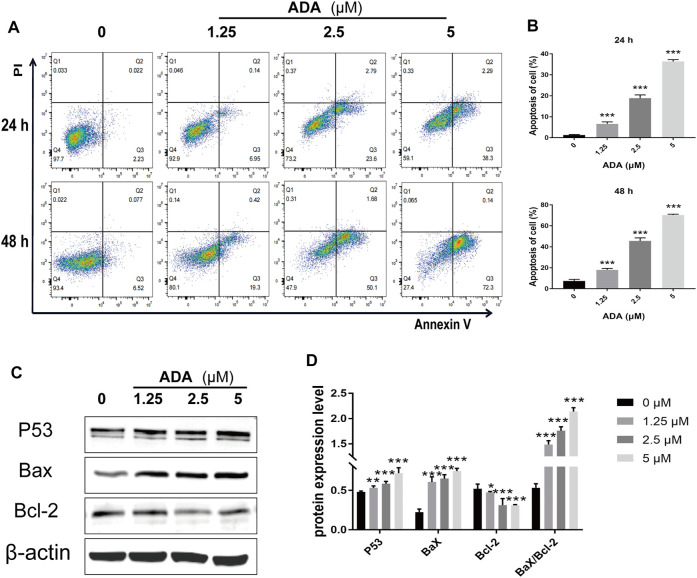
ADA significantly induced apoptosis and regulated the expression of related proteins in RM-1 cells. RM-1 cells were treated with ADA (0, 1.25, 2.5, and 5 μM) for 24 or 48 h respectively. **(A)** Cell apoptosis was detected by Annexin V-APC/PI double staining. **(B)** Histograms showed the proportion of apoptosis cells by flow cytometry. **(C)** Bax, Bcl-2, and P53 protein expression was evaluated utilizing western blotting after 24 h, and **(D)** histograms showed quantitative protein analyzes and the Bax/Bcl-2 ratio β-actin acted as the loading control. The samples derive from the same experiment and that gels/blots were processed in parallel. Data are articulated as mean ± standard deviation. (*p < 0.05; **p < 0.01; ***p < 0.001 vs. control).

After treatment with ADA (0, 1.25, 2.5, and 5 μM) for 24 or 48 h, respectively, compared with the controls, the percentage of apoptotic cells increased from 2.23% to 6.95%, 23.6%, and 38.3% in RM-1 cells after 24 h, 6.52% to 19.3%, 50.1%, and 72.3% after 48 h (Figures 4A,B). Furthermore, we detected Bax and Bcl-2 activation by Western blotting analysis and found that ADA treatment elevated Bax and suppressed Bcl-2 expression, and upregulated the Bax/Bcl-2 ratio in RM-1 cells in a dose-dependent manner (Figures 4C,D). Therefore, ADA promoted apoptosis, which may be responsible for the antiproliferative effect induced in RM-1 cells.

### Adapalene Induced Cell Cycle Arrest by Activating the DNA Damage/p53/p21 Pathway in RM-1 Cells

**FIGURE 5:**
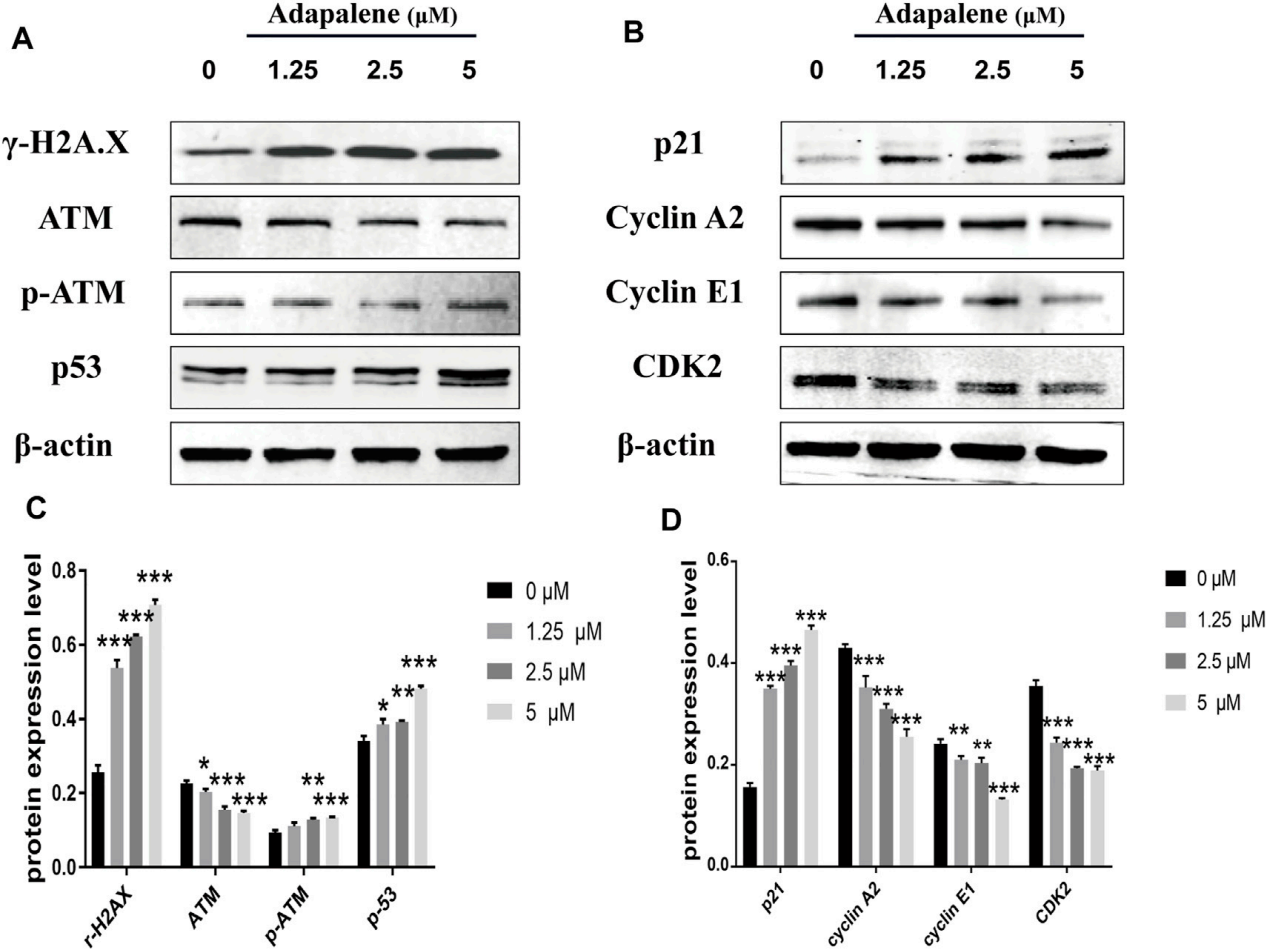
ADA triggered a significant DNA damage pathway/p53/p21 in RM-1 cells. RM-1 cells were treated with the specified dosages of ADA for 24 h. **(A,B)** The protein expressions of γ-H2A.X, ATM, p-ATM, p53, p21, Cyclin A2, Cyclin E1, and CDK2 were analyzed by western blotting, and **(C,D)** histograms showed the protein quantitative analyses, and β-actin acted as the loading control. The samples derive from the same experiment and that gels/blots were processed in parallel. Data are articulated as mean ± standard deviation. (*p < 0.05; **p < 0.01; ***p < 0.001 vs. control).

To assess the affect of ADA on cell DNA damage and repair, we assessed the expression of DNA damage, DNA repair, and cell cycle-related proteins by Western blotting in RM-1 cells following ADA treatment. The expression of γ-H2A.X, P-ATM, p53, and p21 in ADA-treated RM-1 cells was dramatically elevated in a dosage-dependent manner, and the expression of ATM, Cyclin A2, Cyclin E1, and CDK2 was lowered in a dose-dependent manner (Figures 5A–D). Above all, the findings illustrated that ADA mediated its anticancer effects through the activation of the DNA damage/p53/p21 pathway and inhibition of DNA repair may be due to the arrest of the cell cycle in RM-1 cells.

### Adapalene Inhibited Tumor Growth and Inhibited Osteolytic Lesions *in vivo*

**FIGURE 6:**
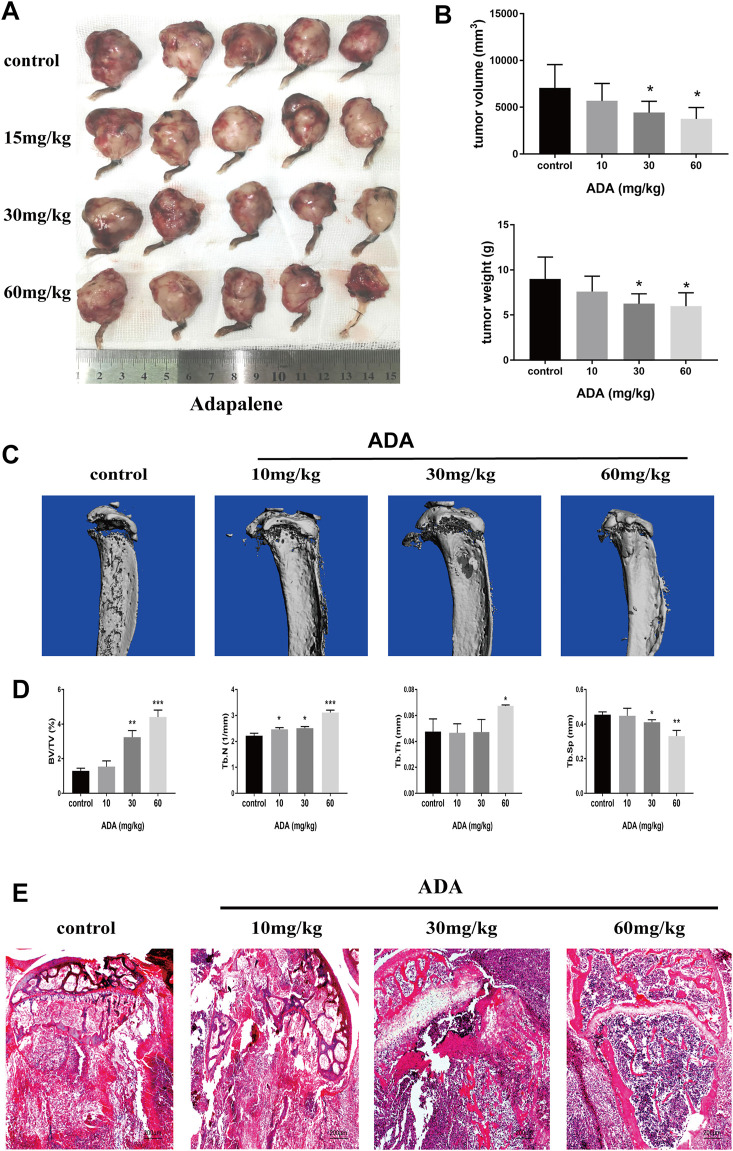
ADA significantly suppressed tumor growth and osteolytic lesions in mice bone metastased with RM-1 cells. Bone metastasis mice were sacrificed after treatment following treatment with increasing doses of ADA (15, 30, and 60 mg/kg) for 14 days. **(A)** Tumors were measured and photographed at the end of treatment. **(B)** Analysis of tumor volume and weight. **(C)** The evaluation of osteolytic lesions and architecture were determined using a micro‐CT scanner. **(D)** Statistical analysis of bone parameters in osteolytic lesions, including trabecular separation (Tb.Sp, mm), connectivity density (1/mm^3^), trabecular thickness (Tb.Th, mm), trabecular number (Tb.N, 1/mm), and bone volume/tissue volume (BV/TV,%). **(E)** Bone tumor tissues were detected using Hematoxylin and eosin staining (100×). Data are articulated as mean ± standard deviation. (*p < 0.05; **p < 0.01; ***p < 0.001 vs. control).

To confirm the anticancer effects of ADA *in vivo*, we used a bone metastasis mouse model for further studies. The mice were treated with varying doses of ADA (10, 30, and 60 mg/kg in 0.5% CMC-NaCl) by oral gavage each day for 14 days; all the mice survived till the end of the experiment. We found that tumor volume and weight reduced in mice treated with 30 and 60 mg/kg than in control mice, while mice treated with 10 mg/kg ADA showed no considerable difference from the control mice (Figures 6A,B). Areas of osteolytic lesions and architecture were determined using a high‐resolution micro‐CT scanner (Figure 6C). Morphometric parameters of the osteolytic lesions, including trabecular separation (Tb.Sp, mm), connectivity density (1/mm^3^), trabecular thickness (Tb.Th, mm), trabecular number (Tb.N, 1/mm), and bone volume/tissue volume (BV/TV,%), were measured. We found that the microarchitectural bone parameters, namely, Tb.Th, Tb.N, and BV/TV,% was greater in mice treated with high doses of ADA(30 and 60 mg/kg) than in control mice, and the associated reduction in Tb.Sp was also detected in mice treated with high doses of ADA while no significant difference from control mice was found in mice receiving lower doses of ADA (Figure 6D). Furthermore, the tissue of bone tumor metastases was stained with H&E (Figure 6E). We found that in control mice, all trabecular bones were destroyed and the growth plate and bone marrow cavity were completely replaced by metastatic RM-1 cells. In contrast, ADA-treated mice showed reduced colonization of metastatic RM-1 prostate cancer cells and part of the trabecular bones remained intact.

### Adapalene Inhibited the Expression of Ki-67 and Promoted Apoptosis in Mice Bone Metastasized With RM-1 Cells

**FIGURE 7:**
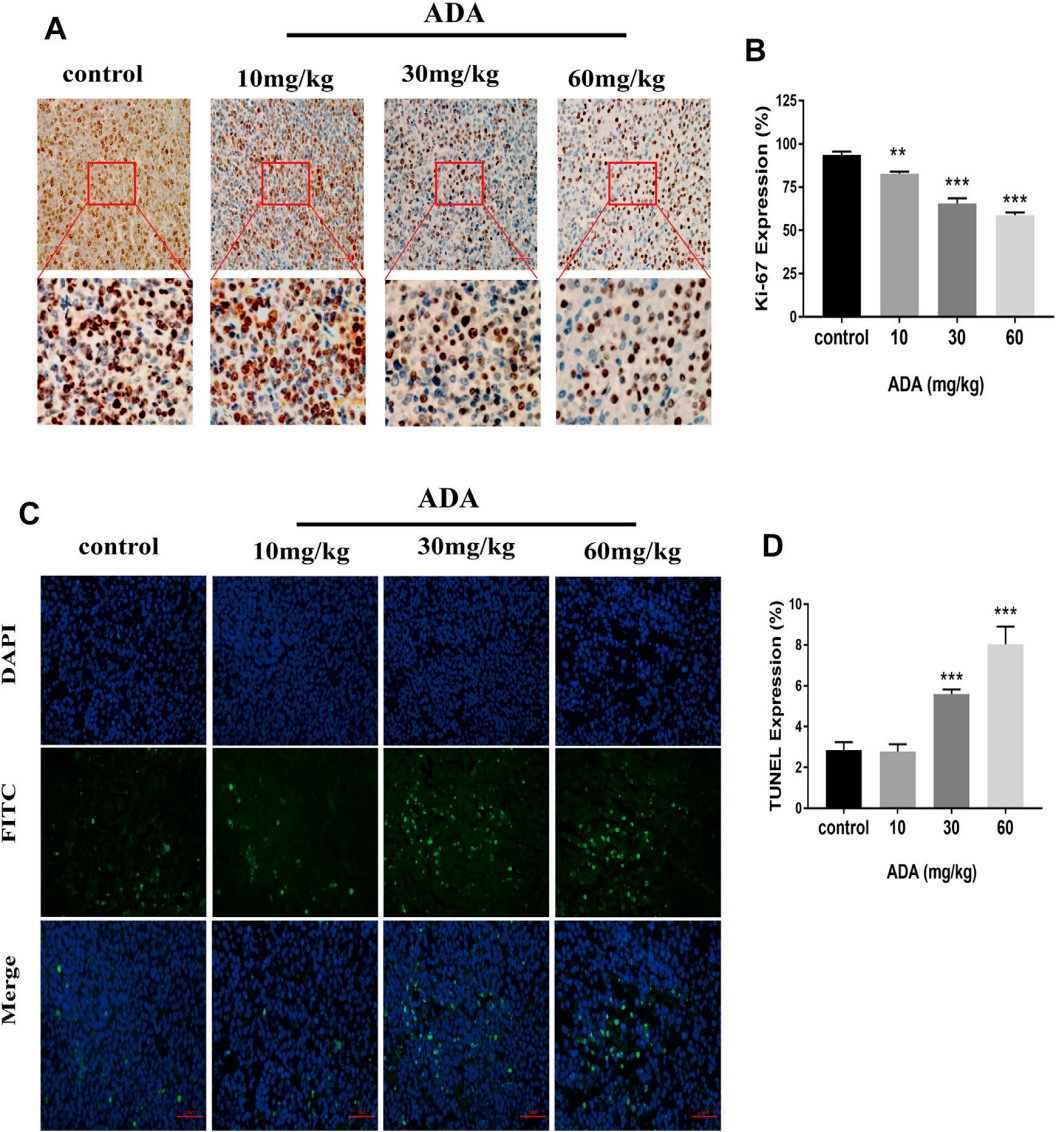
ADA inhibited the Ki-67 expression and promotes the apoptosis of RM-1 prostate cancer cells in tumor tissues. **(A)** The expression of Ki-67 was analyzed by immunohistochemistry (IHC) staining (200×). **(B)** Histograms showing quantitative Ki-67 expression analyses. **(C)** The RM-1 cells were stained by TUNEL staining (400×). **(D)** Histograms showing the quantitative percentage of TUNEL-positive cells. Data are presented as mean ± standard deviation. (*p < 0.05; **p < 0.01; ***p < 0.001 vs. control).

The Ki-67 expression in tumor tissues was analyzed by immunohistochemical staining (Figures 7A,B). The expression of Ki-67 was greatly reduced in the tumor tissues of ADA-treated mice than in control mice. Further, the tumor tissues were stained by TUNEL staining (Figure 7C). The proportion of TUNEL-positive cells was substantially elevated in ADA-treated mice than in control mice (Figure 7D). Taken together, these findings indicate that ADA significantly inhibited the growth of RM-1 xenografts in mice and induced apoptosis in a dose-dependent manner in RM-1 prostate cancer cells.

## Discussion

Prostate cancer is the most malignant cancer of the male urinary tract and often presents with bone invasion and metastasis. ADA is a third-generation synthetic retinoid with anti-inflammatory and anti-cancer activity. In this study, we assessed the impact of ADA on the proliferation of the prostate cancer cell line RM-1.

Almost all tumors present the same characteristic of uncontrolled cell proliferation (Dick, 2008), and the objective of anti-tumor therapy is to suppress tumor cell proliferation and invasion and to promote tumor cell apoptosis. To confirm the anti-proliferative effects of ADA on RM-1 cells, CCK-8 analysis, colony formation, migration, and invasion assays were performed *in vitro*. As expected, the results of the CCK-8 analysis revealed that ADA suppressed the growth of RM-1 cells in a dose- as well as time-dependent manner. With time, ADA significantly inhibited the colony-forming ability of RM-1 cells. In addition, ADA also decreased the migration ability and invasiveness of RM-1 cells in a dose- and time-dependent manner. *In vivo* experiments in an RM-1 cell bone metastasis mouse model revealed that ADA effectively inhibited tumor size and weight. Furthermore, we found that ADA inhibited Ki-67 expression in tumor tissue in a dose-dependent manner. The morphometric analysis of microarchitectural bone parameters and tumor tissue by H&E staining revealed that ADA effectively inhibited bone destruction in mice. Taken together, the *in vivo* and *in vitro* studies indicated that ADA effectively inhibited the proliferation of RM-1 cells.

According to several studies, ADA was involved in cell cycle arrest by regulating cell cycle independent kinase (CDK). For instance, ADA inhibits the melanoma cells proliferation by arresting the S phase and subsequently inducing apoptosis caused by DNA damage (Li et al., 2019). ADA has also been shown to suppress the growth of colorectal cancer cells by inhibiting the activity of CDK2 (Ocker et al., 2004; Shi et al., 2015). CDK2 is a significant target for cancer therapy (Roskoski, 2019). CDK2 with its binding partners performs a critical function throughout the cell cycle progression (Bertoli et al., 2013). The CDK2 complexes with cyclin E or cyclin A and is necessary for the initiation and progression of the S phase, and the CDK1 complexes with cyclin A or cyclin B are essential for cell mitosis (Malumbres and Barbacid, 2009). CDK2 can drive the cell cycle into the S phase and initiate replication; CDK2 activity increases with cell entry into the G2 phase and the S phase (Spencer et al., 2013). During phase transition from G1- to S- phase, the cyclin partners switch from cyclin E to CyclinA, and the CyclinE-CDK2 and CyclinD1-CDK4 complexes are activated sequentially and Rb is hyper-phosphorylated (Cobrinik, 2005; Ditano et al., 2021). In this study, we investigated cell cycle distribution by flow cytometry and found that ADA induced S phase arrest in RM-1 cells after ADA treatment. We also assessed the expression of cell cycle-related proteins in RM-1 cells and found that ADA triggered S-phase arrest in RM-1 cells by inhibiting CDK2, Cyclin A2, Cyclin E1, Rb, and P-Rb at 24 h. During the transition from the G1- to S-phase, CDK2, Cyclin A2, Cyclin E1, Rb, and P-Rb play a significant role. Therefore, the reduced activity of CDK2/Cyclin A2 that drives entry into the G2 phase from the S-phase, and CDK2/Cyclin E1 and Cyclin A2 downregulation eventually caused the cell cycle arrest in the S-phase.

In addition, we found that ADA induced S phase arrest while promoting apoptosis in RM-1 cells. The percentage of cells arrested in the S-phase increased 44.9% (12 h) and 70.9%(24 h) after treatment with ADA (5 μM), the percentage of apoptotic cells only increased to 38.3% at 24 h but 72.3% at 48 h. The percentage of cell arrest increased at first, subsequently, the percentage of apoptosis increased. Therefore, we postulated that S-phase cell cycle arrest might be a reason for inducing apoptosis after prolonged ADA treatment. ADA also induced up-regulation of the expression of Bax/Bcl-2 as p53 expression increased. Apoptosis is a process of cellular suicide, which usually responds to growth hormone or cytokine exposure, factor deprivation, and DNA damage (Sharma et al., 2012). Some Bcl-2 family members including Bak and Bax are activated by p53, while Bcl-2 is inhibited by p53 (Tang et al., 2021). Previous studies have shown that ADA possesses anti-proliferative and pro-apoptotic function in colon carcinoma and hepatoma cell lines by increasing caspase-3 activity by increasing Bax and reducing Bcl2 expression (Ocker et al., 2003; Ocker et al., 2004). In addition, the p53/p21 complex modulates the invasion as well as apoptosis of cancer cells by targeting Bcl-2 family proteins (Kim et al., 2017). Herein, ADA significantly promoted the expression of p53, and thereby promoted the Bax expression and suppressed the Bcl-2 expression, positively regulating the alteration in the Bax/Bcl-2 ratio, leading to the induction of RM-1 cell apoptosis. Furthermore, TUNEL staining illustrated that the proportion of TUNEL-positive cells was considerably elevated after ADA treatment. Therefore, our study suggests that ADA effectively inhibited proliferation and triggered apoptosis in RM-1 cells.

The fate of cells is determined by the dynamic equilibrium between DNA damage and repair. DNA is vulnerable to environmental and dietary carcinogens, endogenous metabolites, anti-inflammatory drugs, and genotoxic cancer drugs (Roos et al., 2016). The induction of DNA damage activates either interim checkpoints that facilitate the genetic repair or nonreversible growth arrest that leads to cell necrosis and apoptosis (Krüger et al., 2018). Thus, activation of checkpoints encompasses a comprehensive response involving sensors (ATM, CHK, BRCA, and RAD) and effectors (p53, p21, CDK) (Pawlik and Keyomarsi, 2004; He et al., 2005; Wang, 2019). As reports showed that the rapid phosphorylation of H2AX at ser139 (a biomarker of DNA damage) was triggered by ATM in the presence of DNA damage, which is necessary for recruiting DNA-damage response proteins (Tanaka et al., 2006; Yuan et al., 2010). We observed that the expression of P-ATM and γ-H2AX increased significantly in RM-1 cells after ADA treatment, indicating that DNA damage was induced. Consequently, the continuous increase in DNA damage may have induced S-phase arrest of the cell cycle and subsequent cell apoptosis.

P21 is encoded by the CDKN1A gene and has been identified as a CDK regulator that participates in a variety of cell functions, such as cell cycle progression, DNA damage, and cell growth (Lee et al., 1998). As the main inhibitor of CDK2, p21 can arrest the G1/S phase of the cell cycle and inhibit the phosphorylation of retinoblastoma protein (Rb) (Cheng and Scadden, 2002). Thus, p21 is also called CDK-interaction protein (CIP1) or CDKN1A (p21) (Gartel and Radhakrishnan, 2005). p53 tumor suppressor/transcription factor regulates multiple cellular functions, including cell growth, migration, invasion, apoptosis, and aging (Muller et al., 2011). p21 inhibits the growth of tumors by targeting p53, and interaction between p21 and proliferating cell nuclear antigen maintains cell cycle arrest after DNA damage (Xiao et al., 2020). In addition, elevated levels of p21 result in a delay of S phase progression and cell mitosis (Moniaux et al., 2020). We found that the levels of p53 and p21 gradually increased in ADA-treated cells in a dose- and time-dependent manner. In contrast, the expression of CDK2, Rb, and p-Rb, the downstream molecules of p21, decreased gradually with the increase in p21 expression, suggesting that ADA promoted the expression of p21 through a p53-dependent pathway, which may represent the mechanism of action of ADA in prostate cancer treatment.

There are some deficiencies in our research. Although many biological functions and behavior of RM-1 cells with prostate cancer model are close to human cells (Li et al., 2021), our results can not completely represent the effects of ADA in human prostate cancer cells, and further research should be combined with human prostate cancer cell experiments in the future.

**FIGURE 8:**
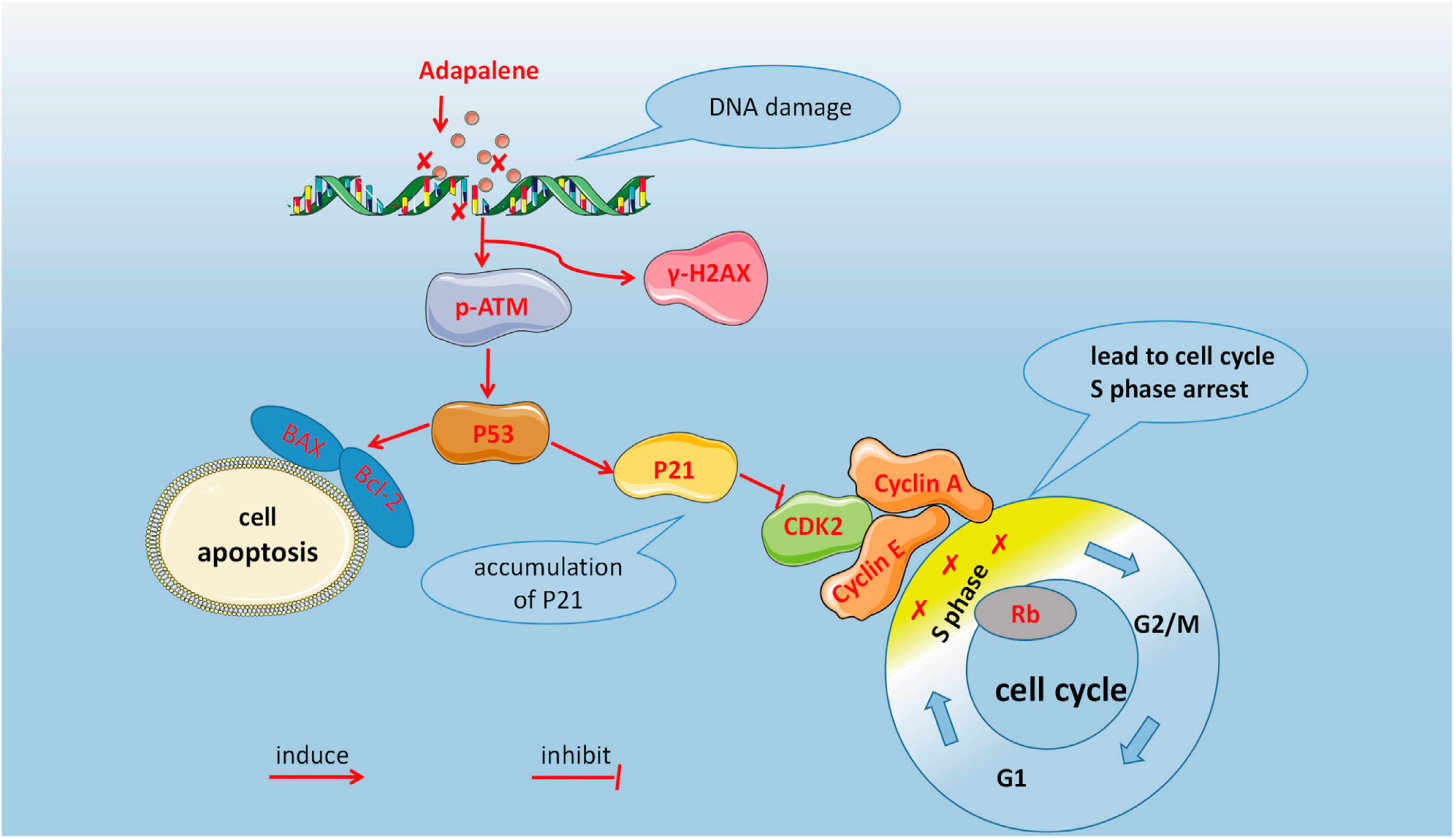
Anticancer mechanism of ADA in RM-1 cells.

In summary, our study showed that ADA inhibits prostate cancer cell proliferation *in vivo* and *in vitro*. As shown in Figure 8, DNA damage activated the ATM/p53/p21 signaling and arrested cell cycle in the S-phase by suppressing CDK2 levels, as well as induced apoptosis by regulating the Bax/Bcl-2 ratio. In conclusion, ADA controls the growth and proliferation of prostate cancer cells likely by inducing DNA damage. Therefore, ADA may be a prospective therapeutic agent for treating prostate cancer.

## Data Availability

The original contributions presented in the study are included in the article/Supplementary Material, further inquiries can be directed to the corresponding authors
