# Supplementary figures and images for: Adapalene Inhibits Prostate Cancer Cell Proliferation In Vitro and In Vivo by Inducing DNA Damage, S-phase Cell Cycle Arrest, and Apoptosis
Source: Front Pharmacol. 2022 Feb 22;13:801624. doi: 10.3389/fphar.2022.801624 (PMC8902295; doi:10.3389/fphar.2022.801624)

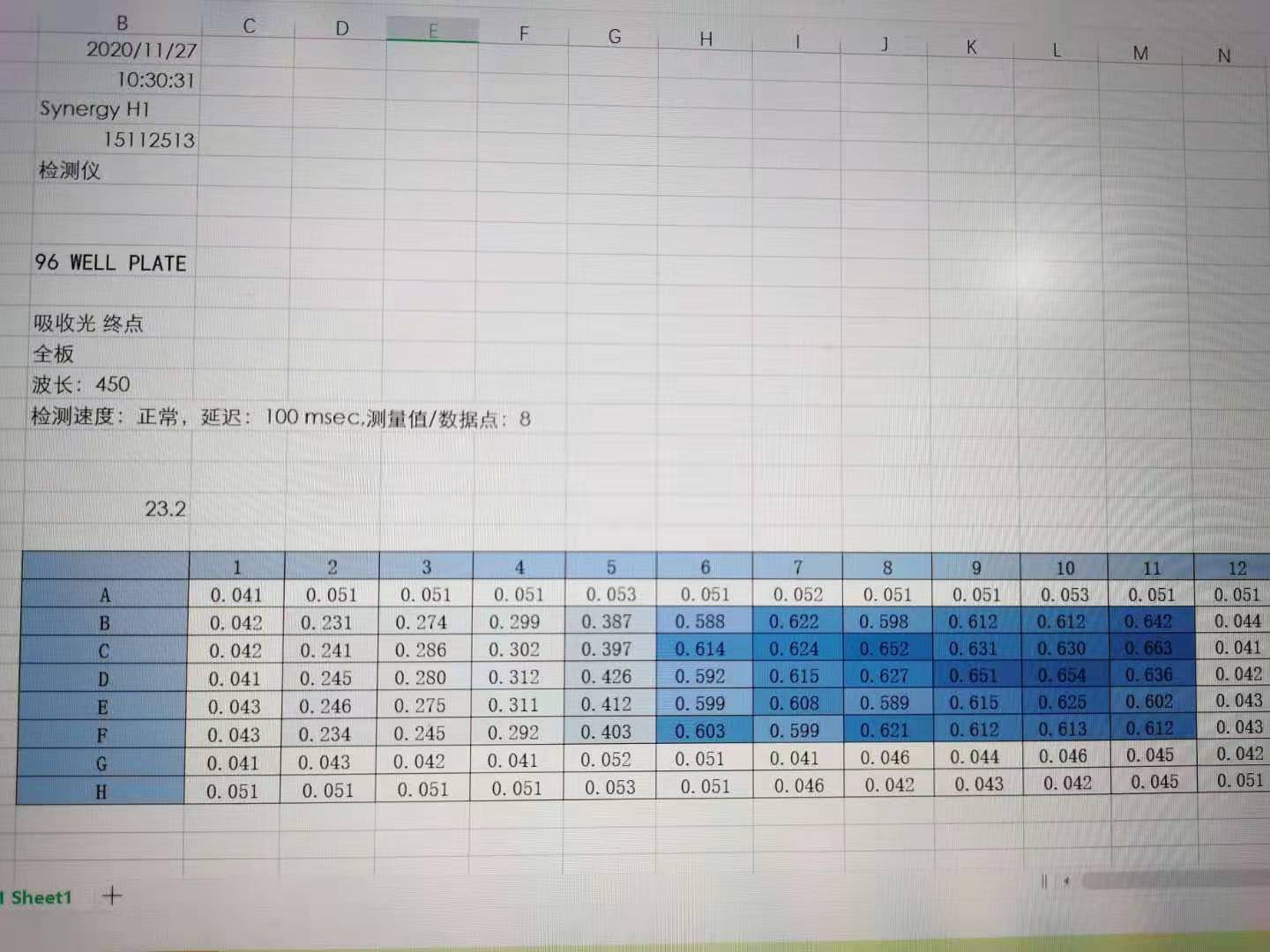

Supplement: Supplementary file 1 [file DataSheet1.ZIP › Raw data/CCK8 SPSS&GraphPad7 statistics/24h.jpg]

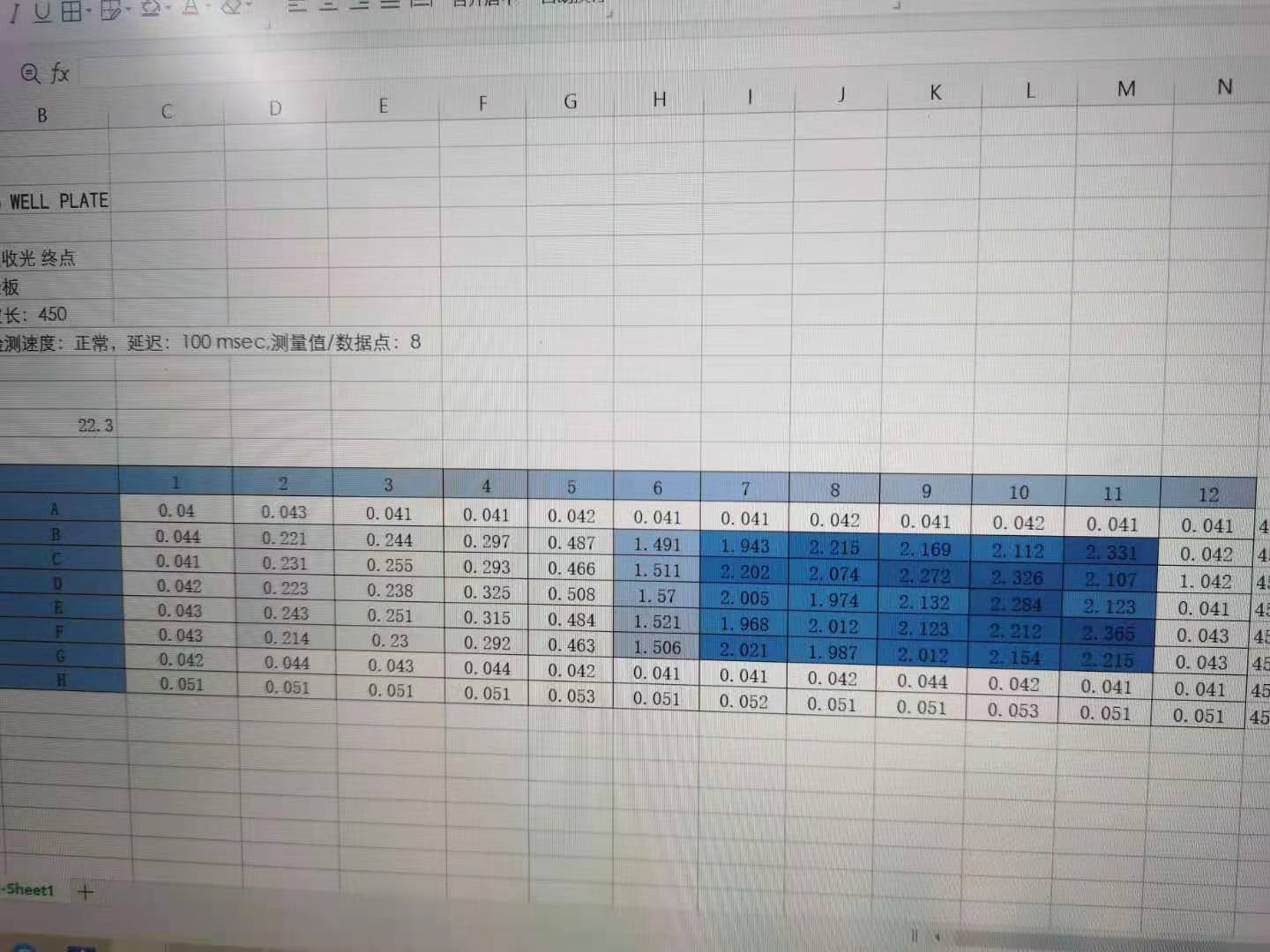

Supplement: Supplementary file 1 [file DataSheet1.ZIP › Raw data/CCK8 SPSS&GraphPad7 statistics/48h.jpg]

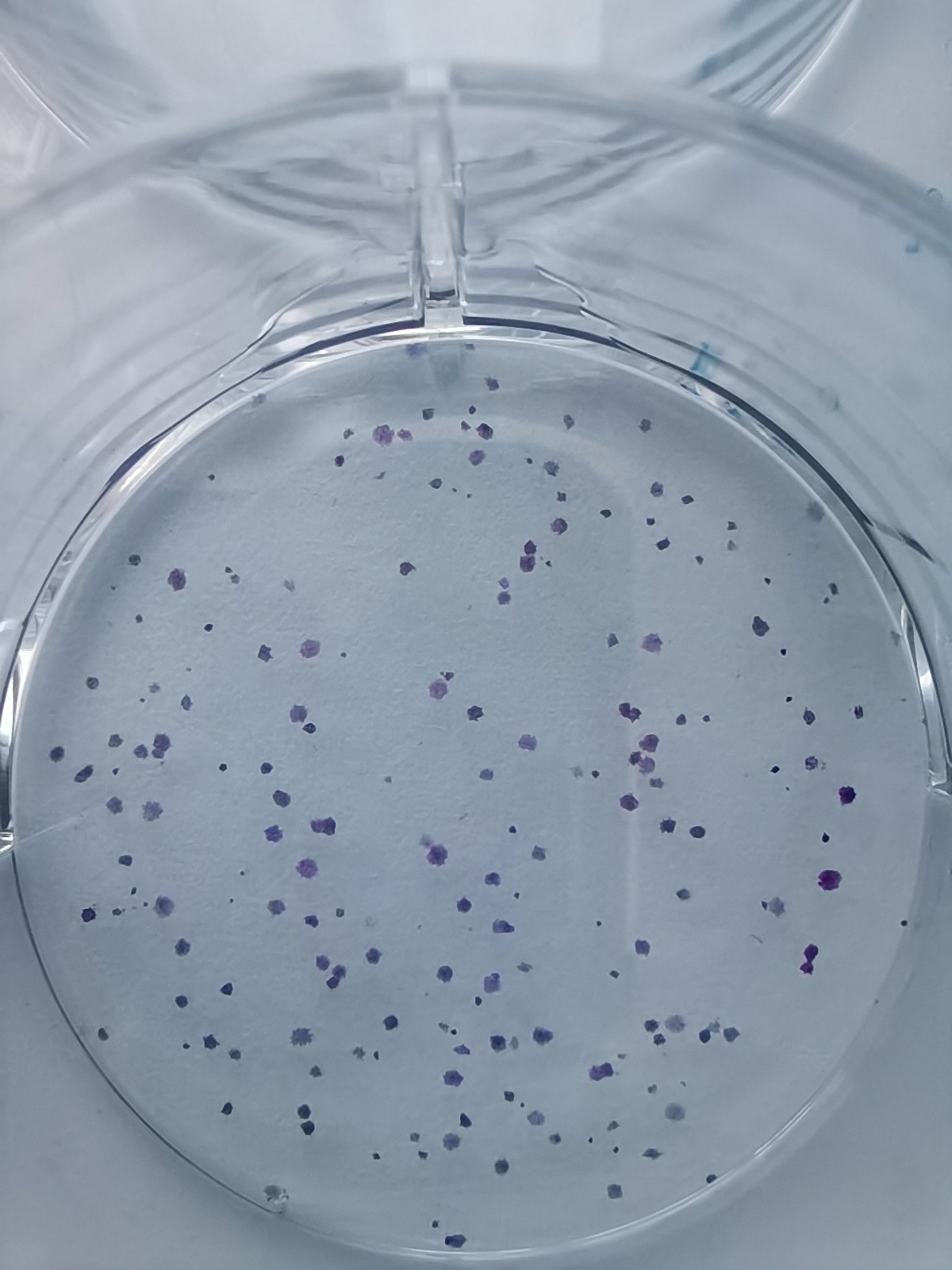

Supplement: Supplementary file 1 [file DataSheet1.ZIP › Raw data/colone picture & data-SPSS&GraphPad7 statistics/0uM.jpg]

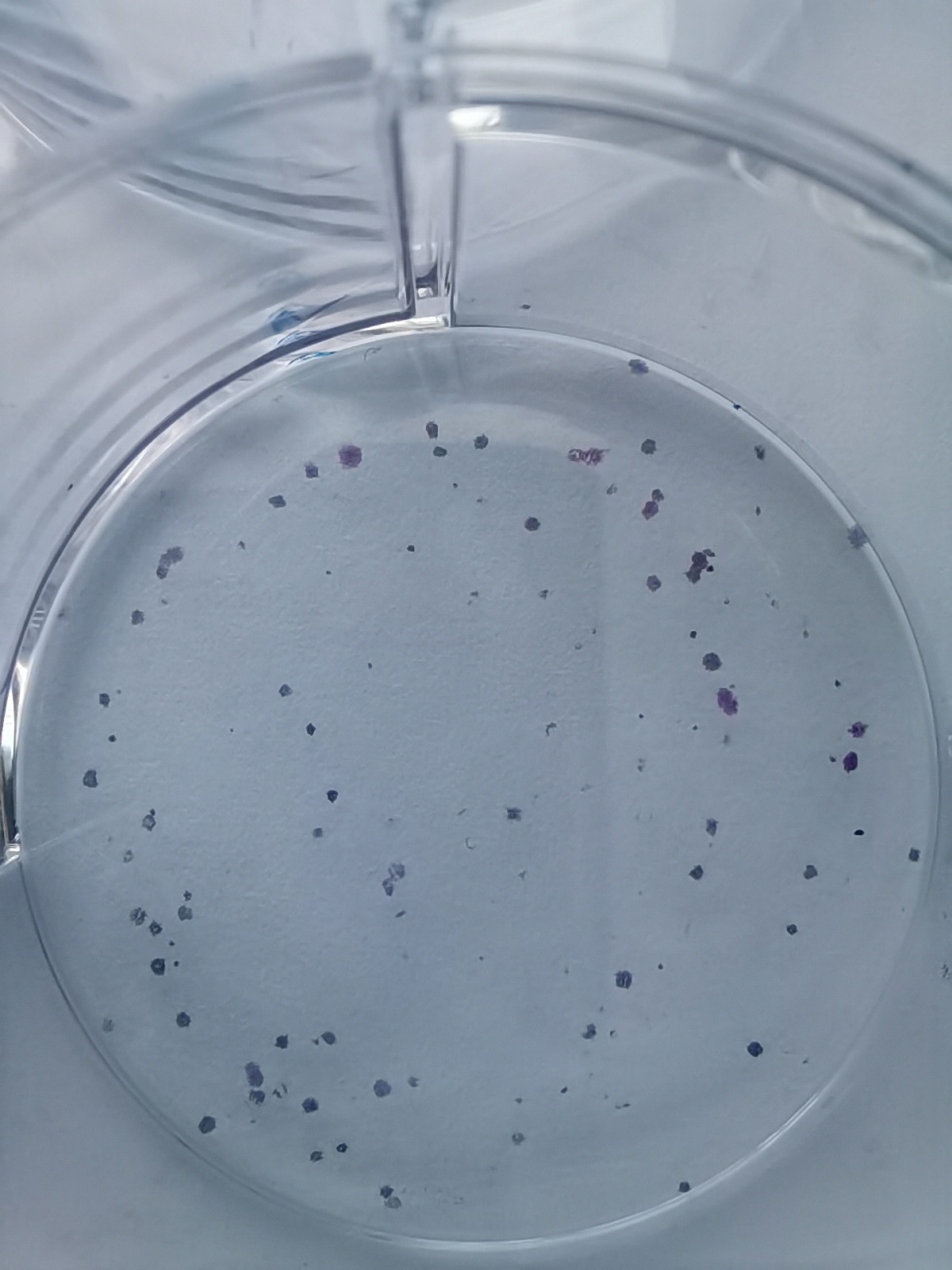

Supplement: Supplementary file 1 [file DataSheet1.ZIP › Raw data/colone picture & data-SPSS&GraphPad7 statistics/1.25uM.jpg]

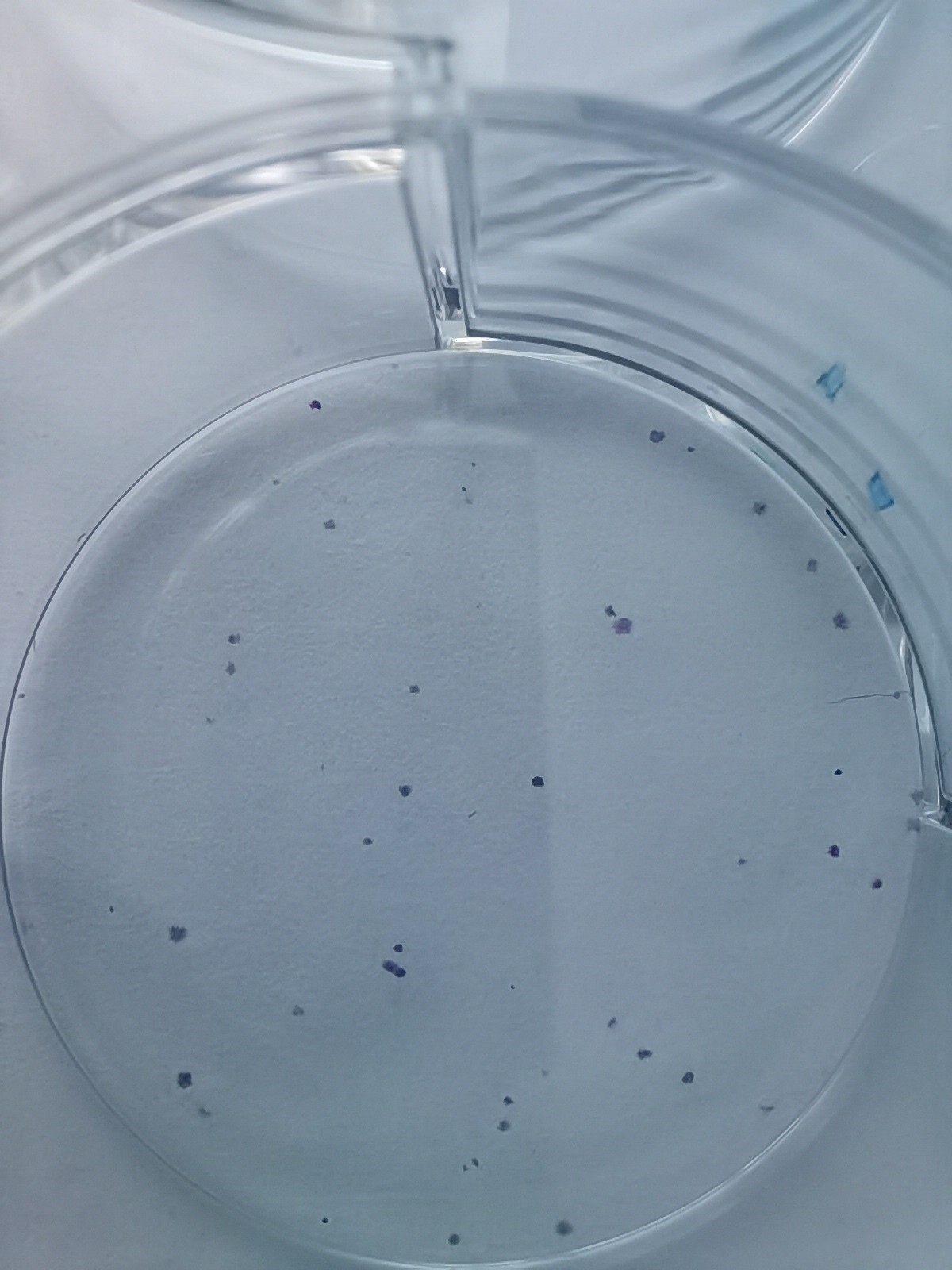

Supplement: Supplementary file 1 [file DataSheet1.ZIP › Raw data/colone picture & data-SPSS&GraphPad7 statistics/2.5uM.jpg]

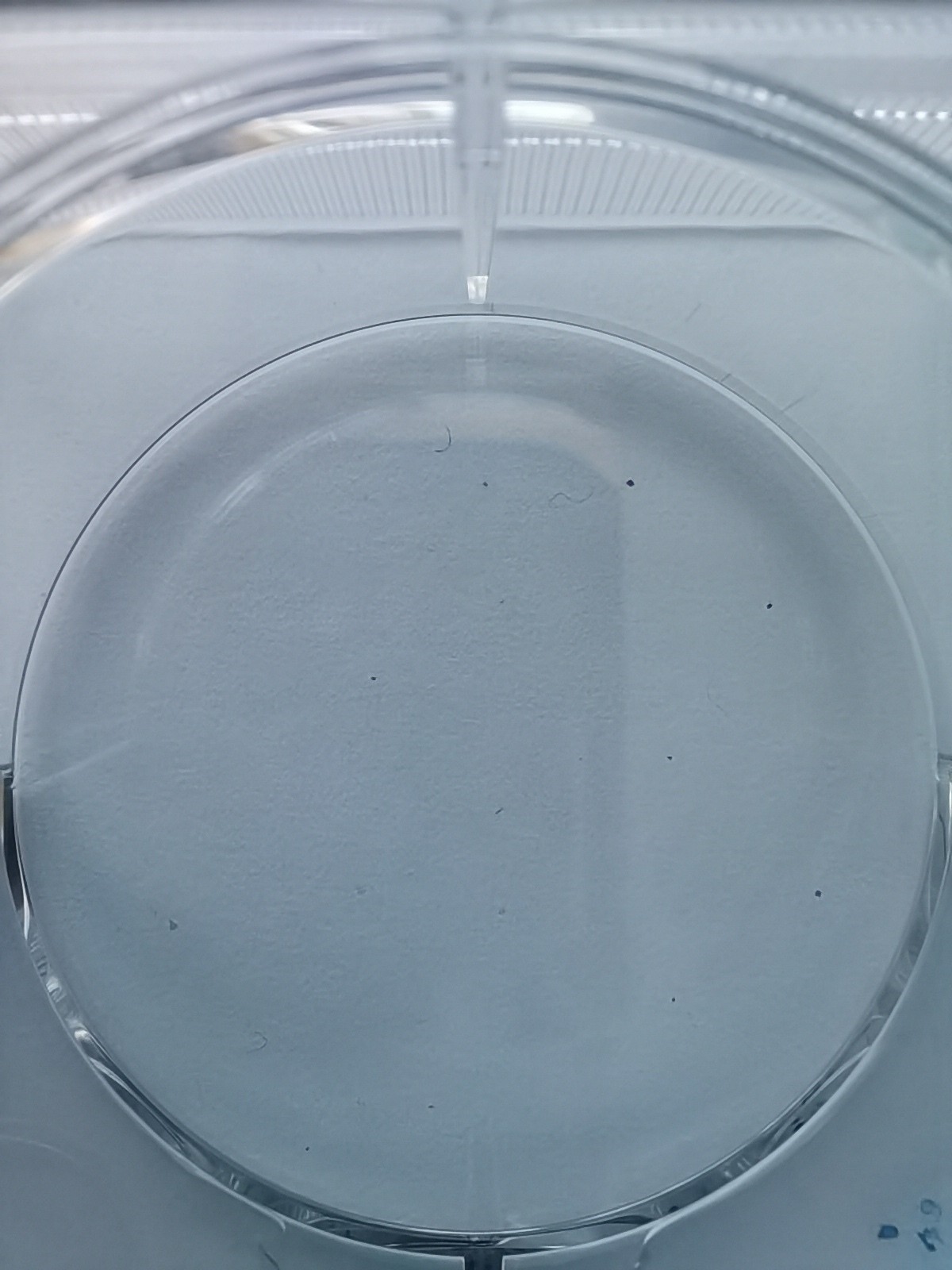

Supplement: Supplementary file 1 [file DataSheet1.ZIP › Raw data/colone picture & data-SPSS&GraphPad7 statistics/5uM.jpg]

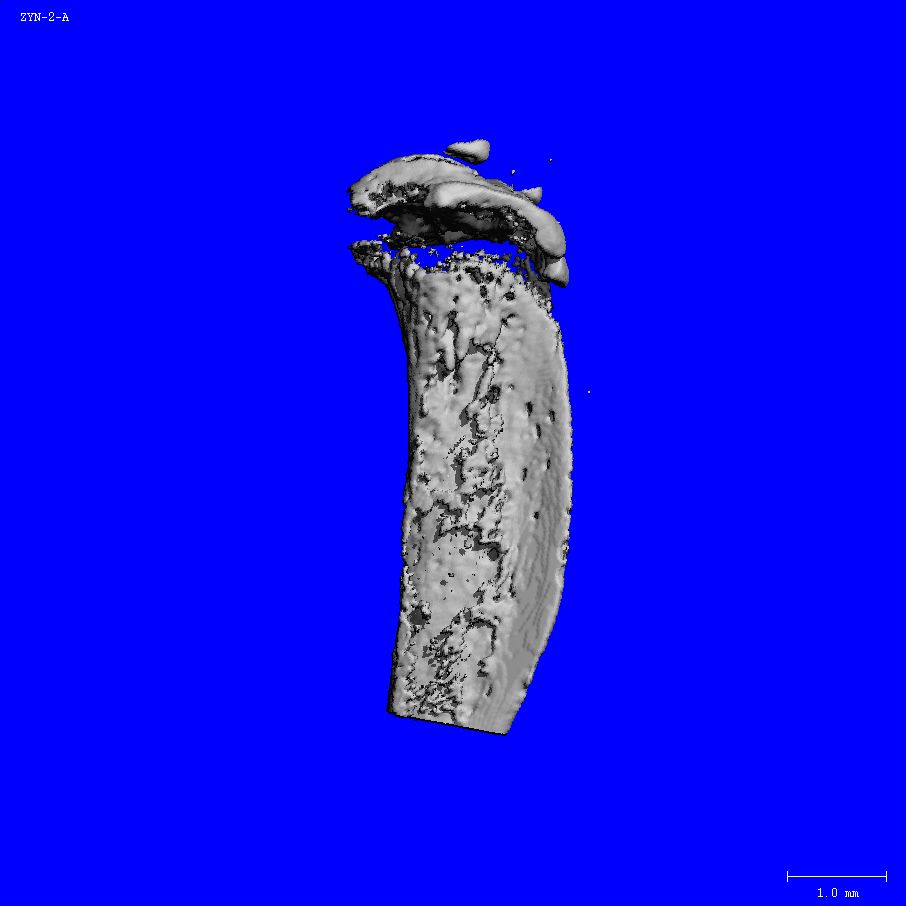

Supplement: Supplementary file 1 [file DataSheet1.ZIP › Raw data/CT picture &data/0mgkg-1.jpg]

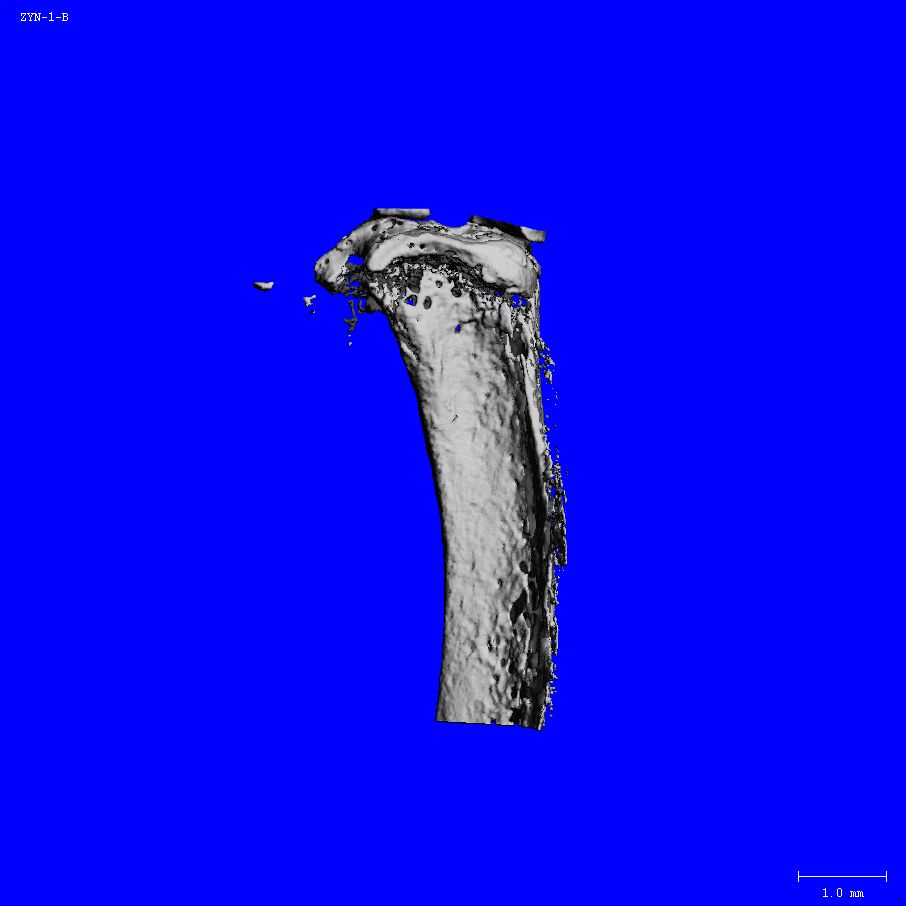

Supplement: Supplementary file 1 [file DataSheet1.ZIP › Raw data/CT picture &data/10mgkg-1.jpg]

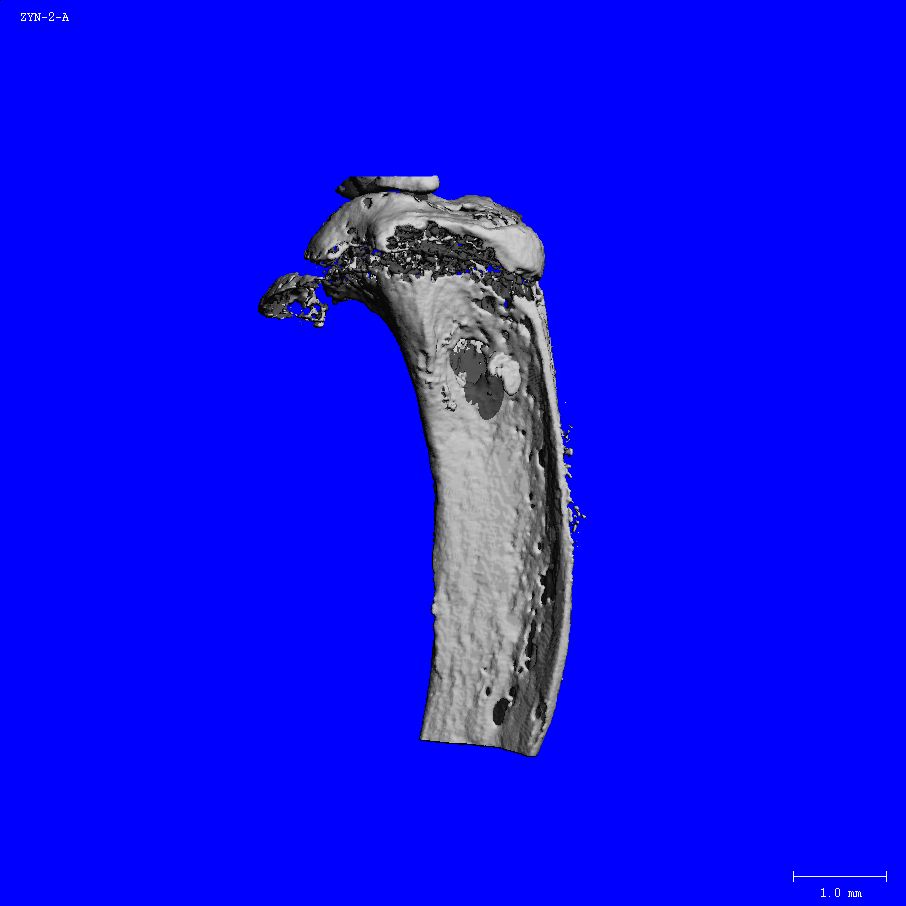

Supplement: Supplementary file 1 [file DataSheet1.ZIP › Raw data/CT picture &data/30mgkg-1.jpg]

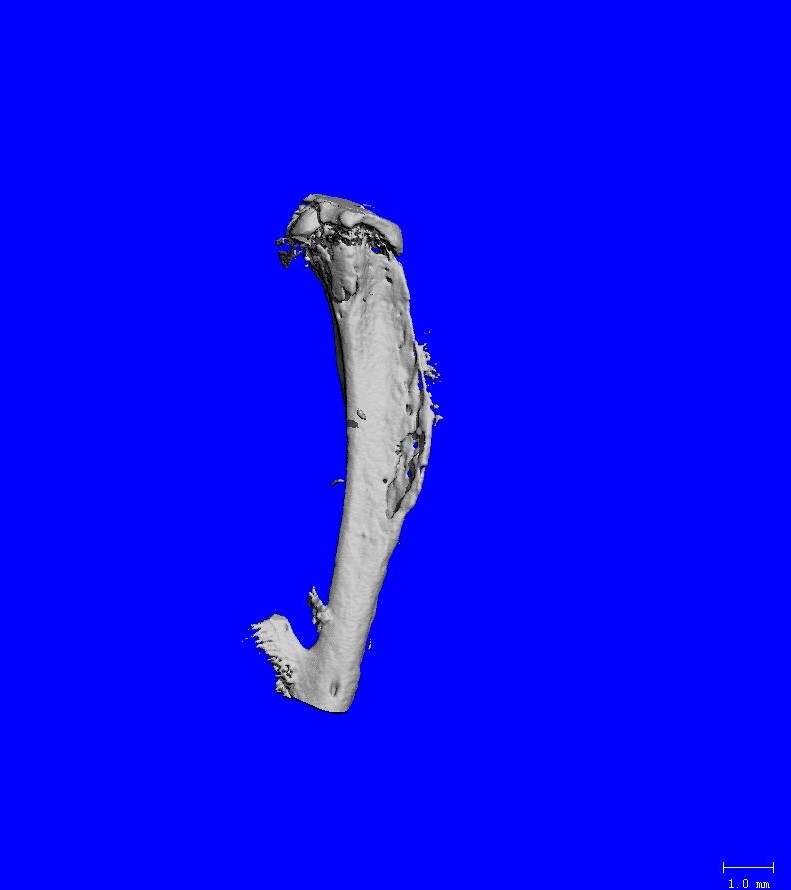

Supplement: Supplementary file 1 [file DataSheet1.ZIP › Raw data/CT picture &data/60-mgkg-1.jpg]

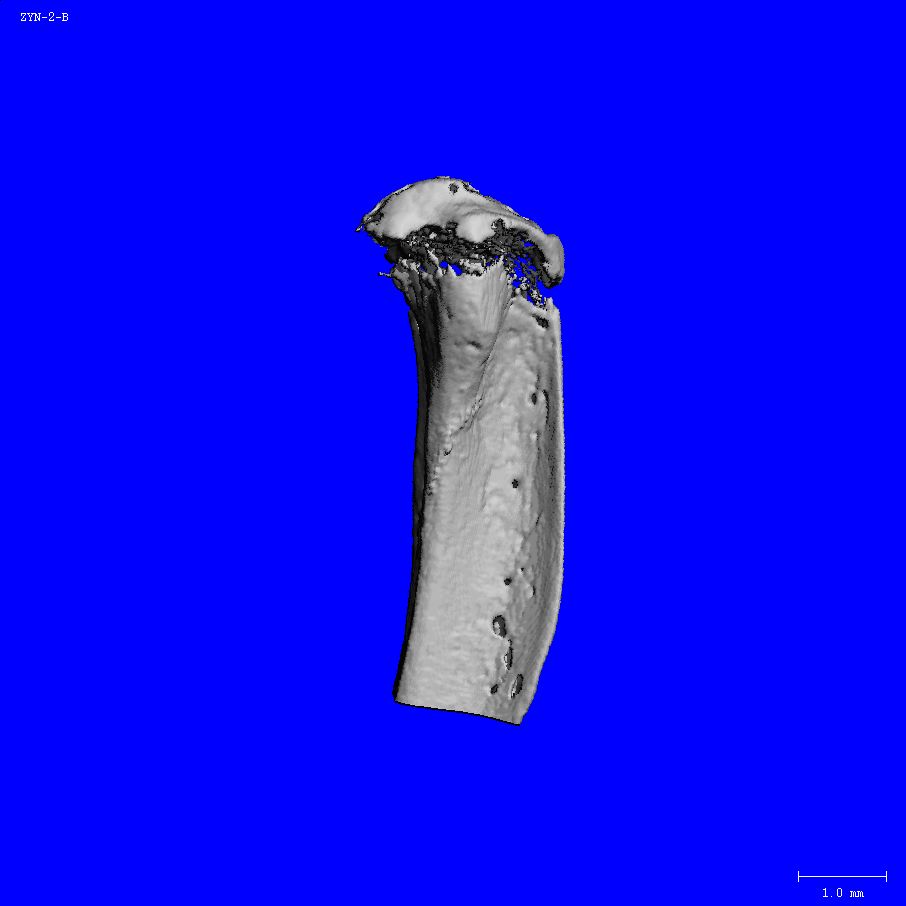

Supplement: Supplementary file 1 [file DataSheet1.ZIP › Raw data/CT picture &data/60mgkg-2.jpg]

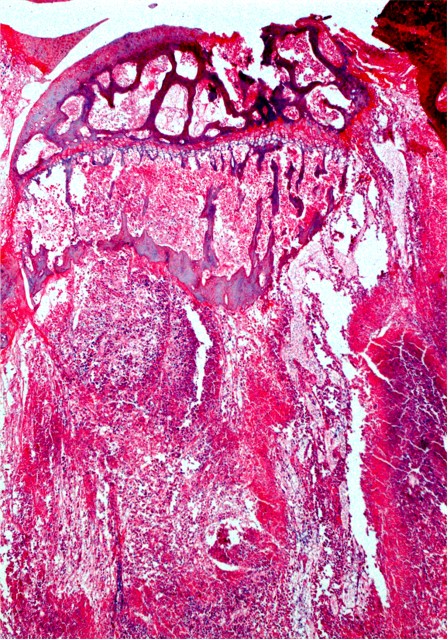

Supplement: Supplementary file 1 [file DataSheet1.ZIP › Raw data/H&E/0-1HE.jpg]

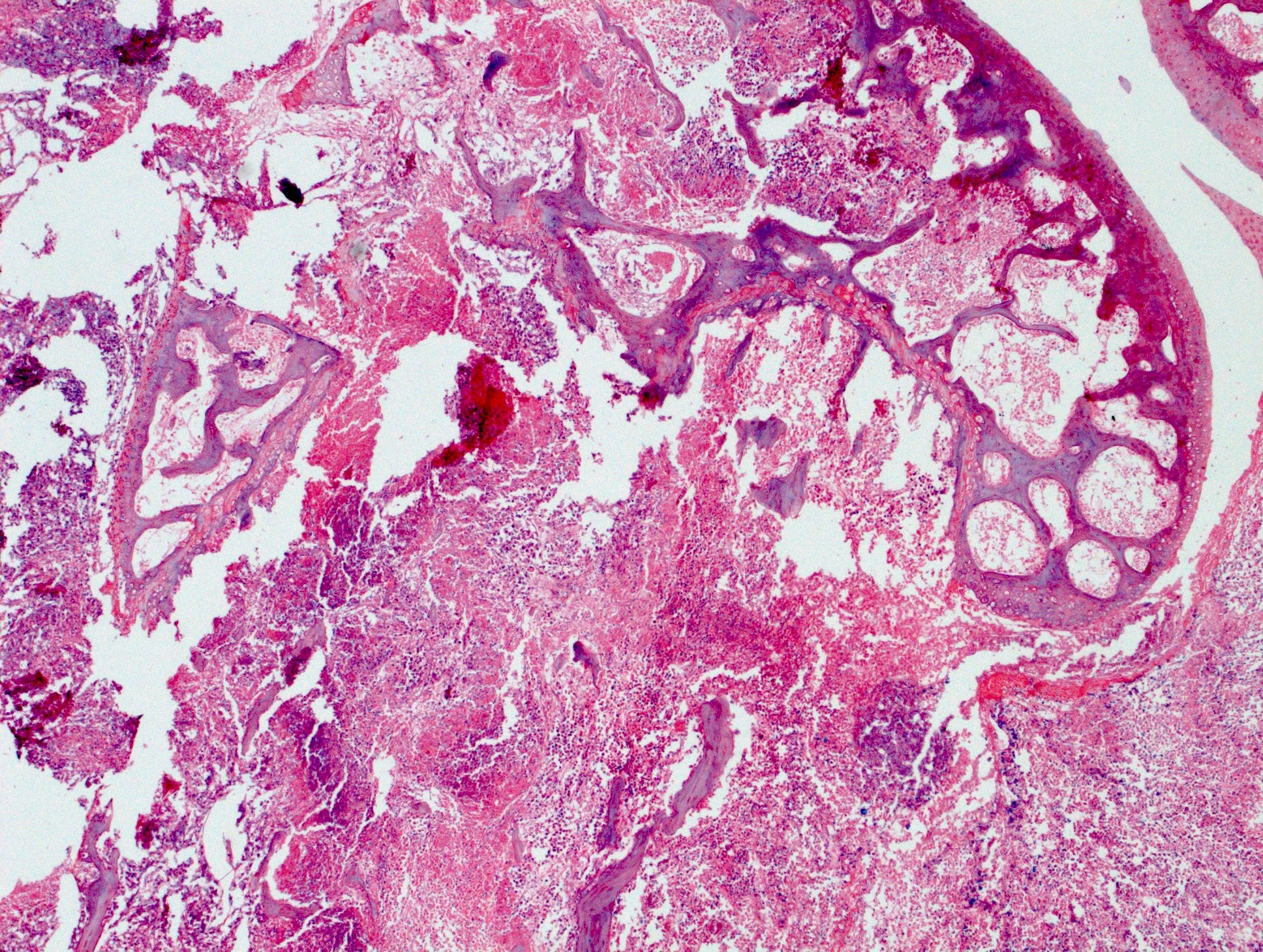

Supplement: Supplementary file 1 [file DataSheet1.ZIP › Raw data/H&E/10-1HE.jpg]

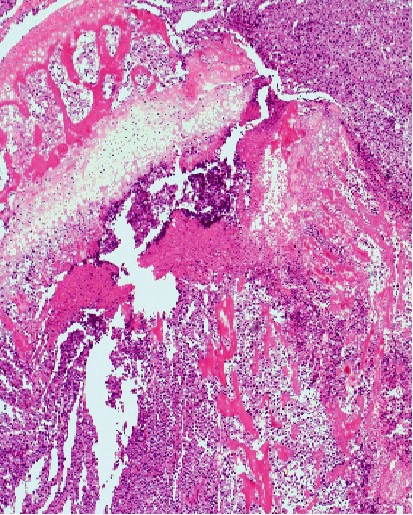

Supplement: Supplementary file 1 [file DataSheet1.ZIP › Raw data/H&E/30-1HE.jpg]

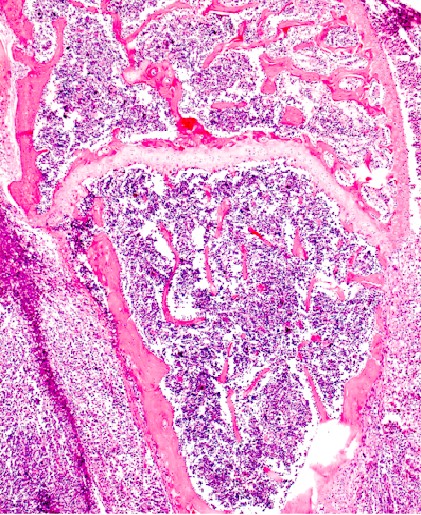

Supplement: Supplementary file 1 [file DataSheet1.ZIP › Raw data/H&E/60-1HE.jpg]

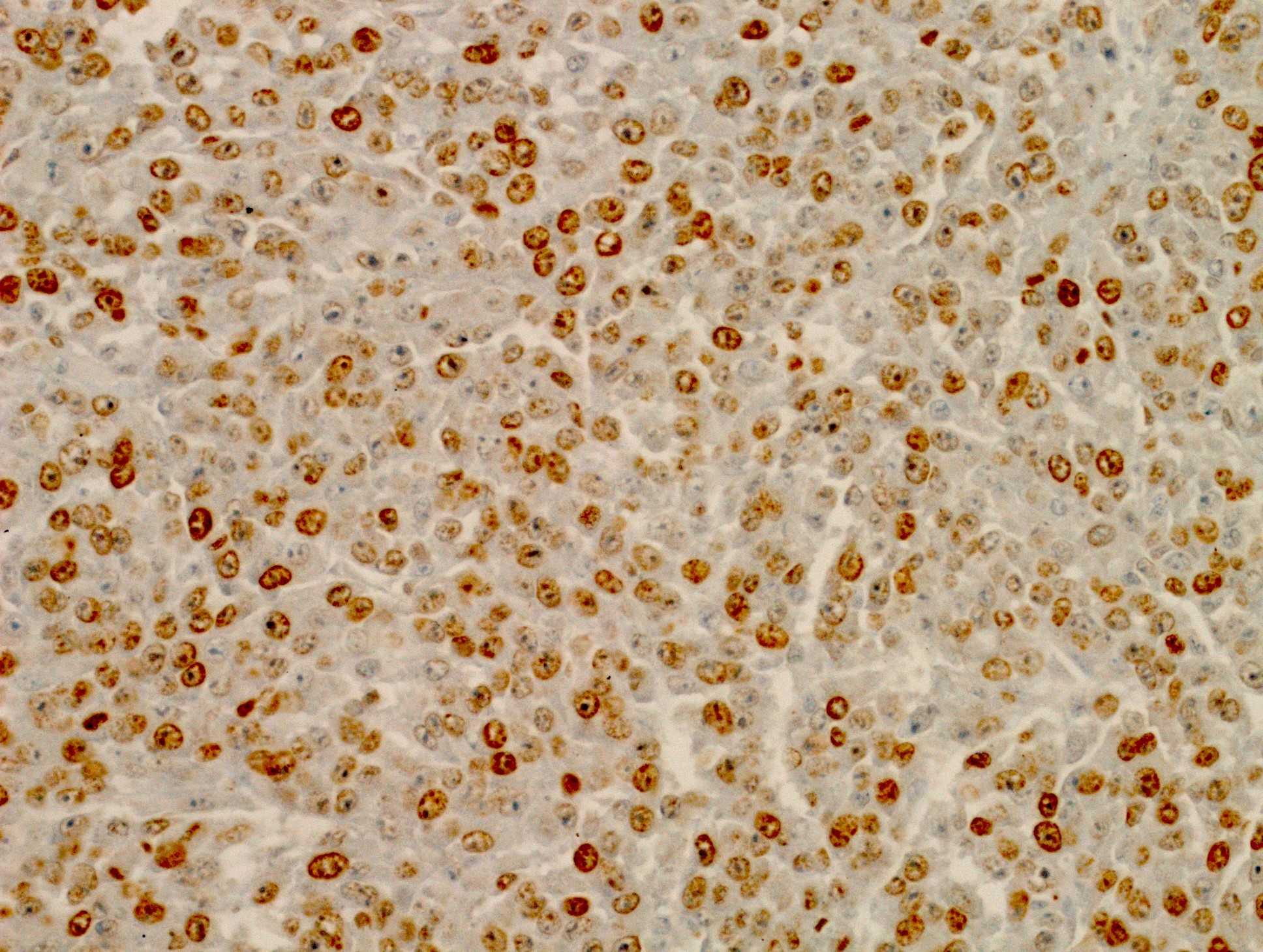

Supplement: Supplementary file 1 [file DataSheet1.ZIP › Raw data/Ki67& TUNEL stain/ki67/0-1-200x.jpg]

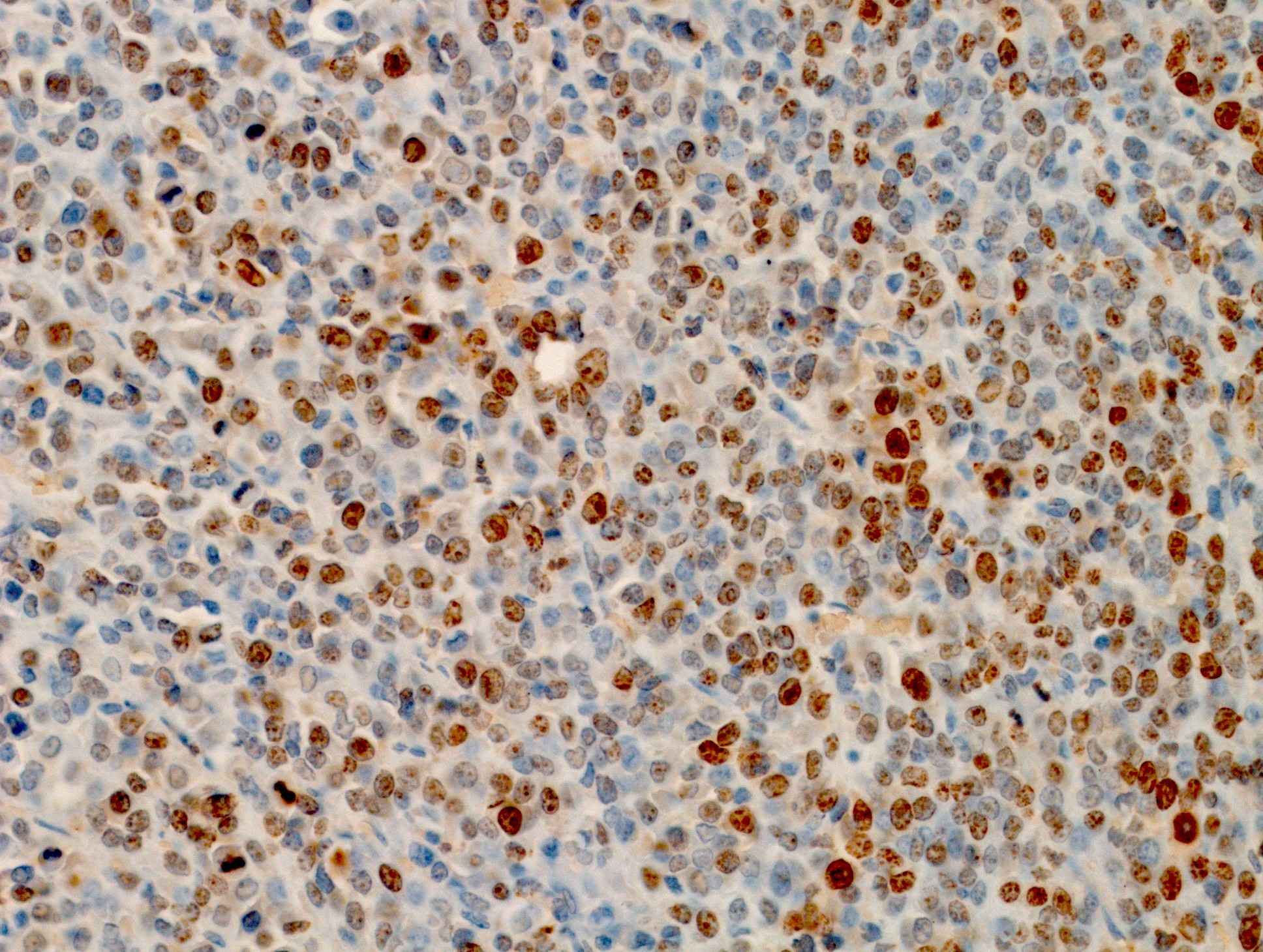

Supplement: Supplementary file 1 [file DataSheet1.ZIP › Raw data/Ki67& TUNEL stain/ki67/10-200x.jpg]

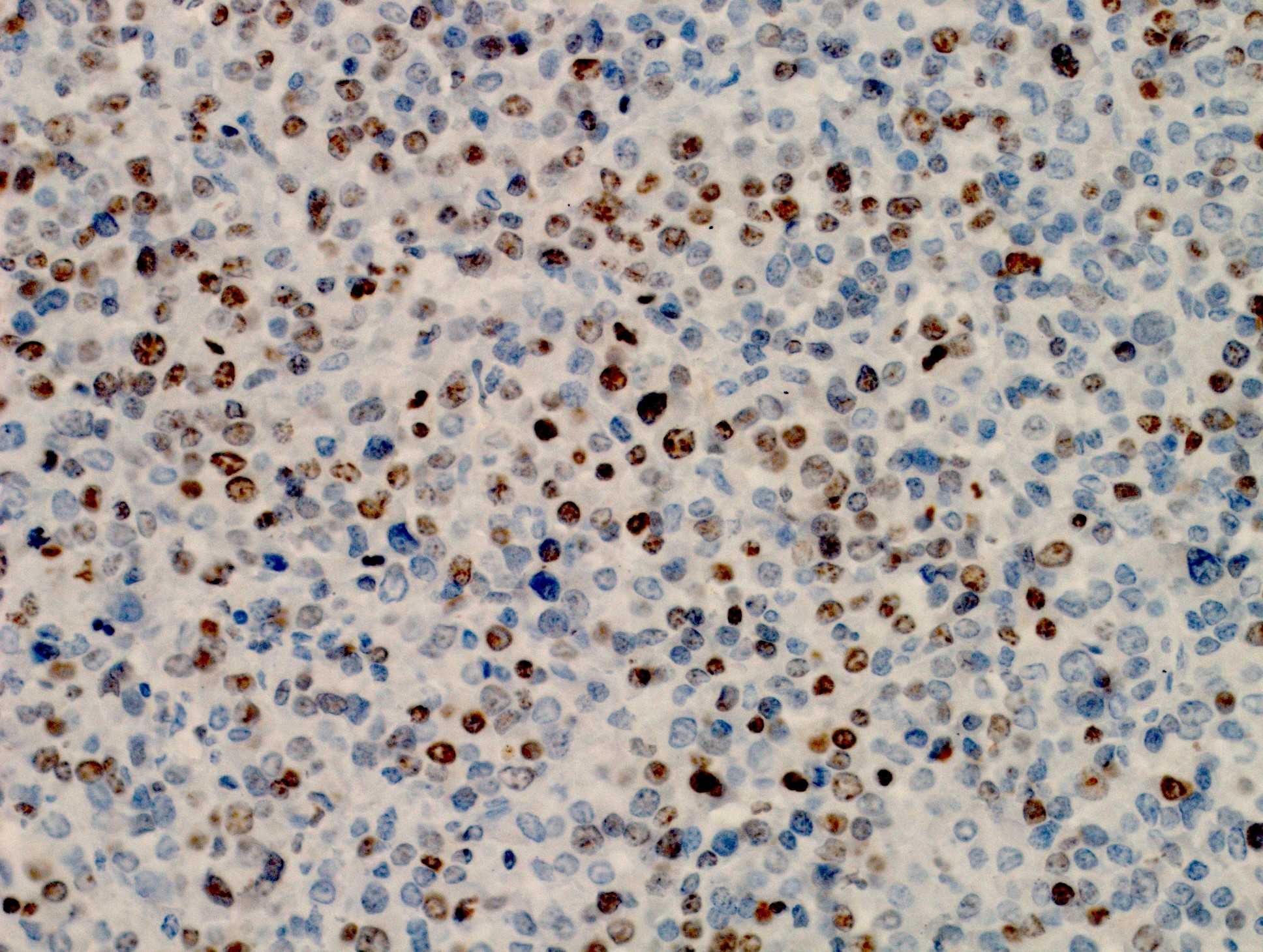

Supplement: Supplementary file 1 [file DataSheet1.ZIP › Raw data/Ki67& TUNEL stain/ki67/30-200X.jpg]

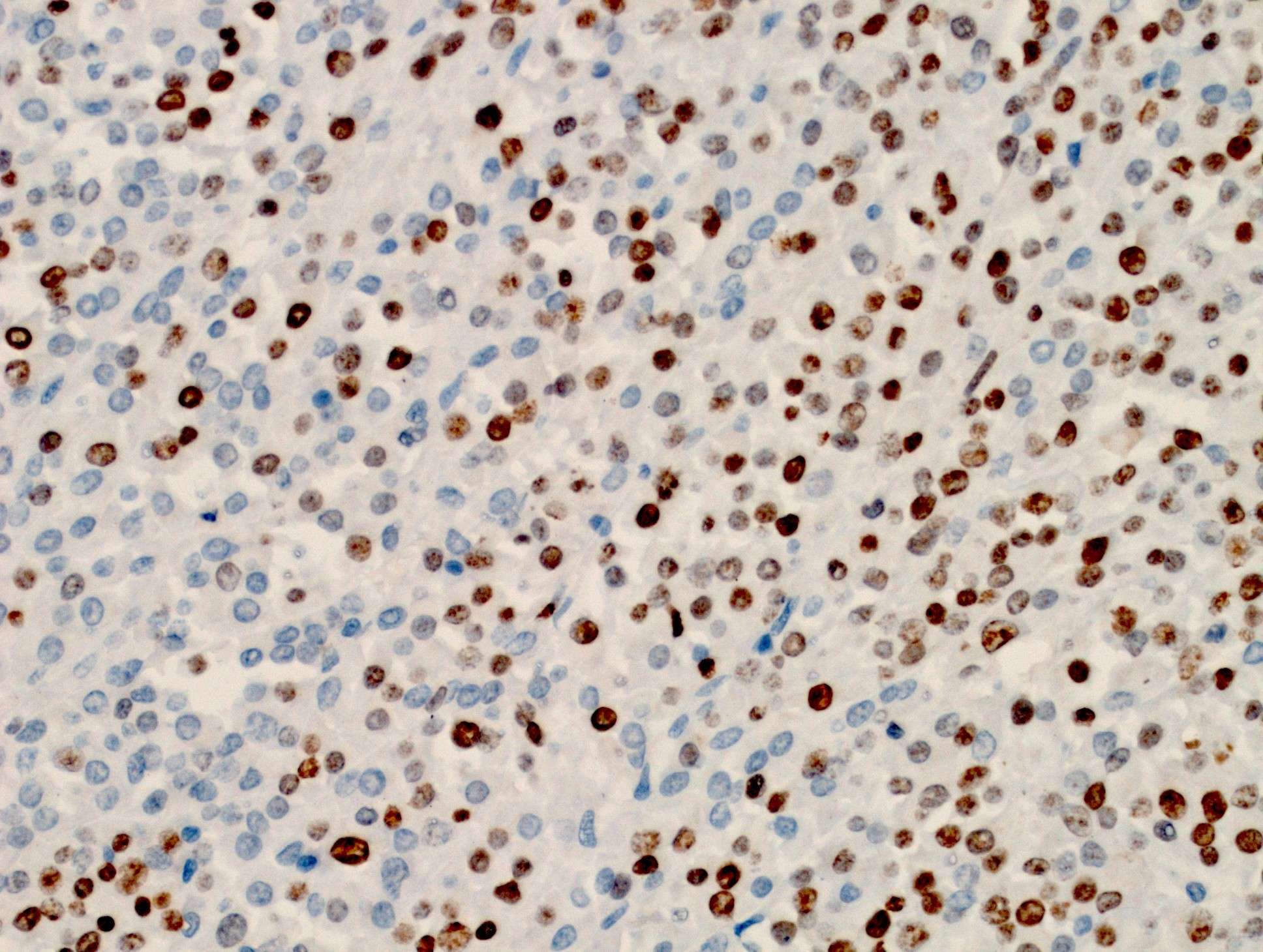

Supplement: Supplementary file 1 [file DataSheet1.ZIP › Raw data/Ki67& TUNEL stain/ki67/60-200x.jpg]

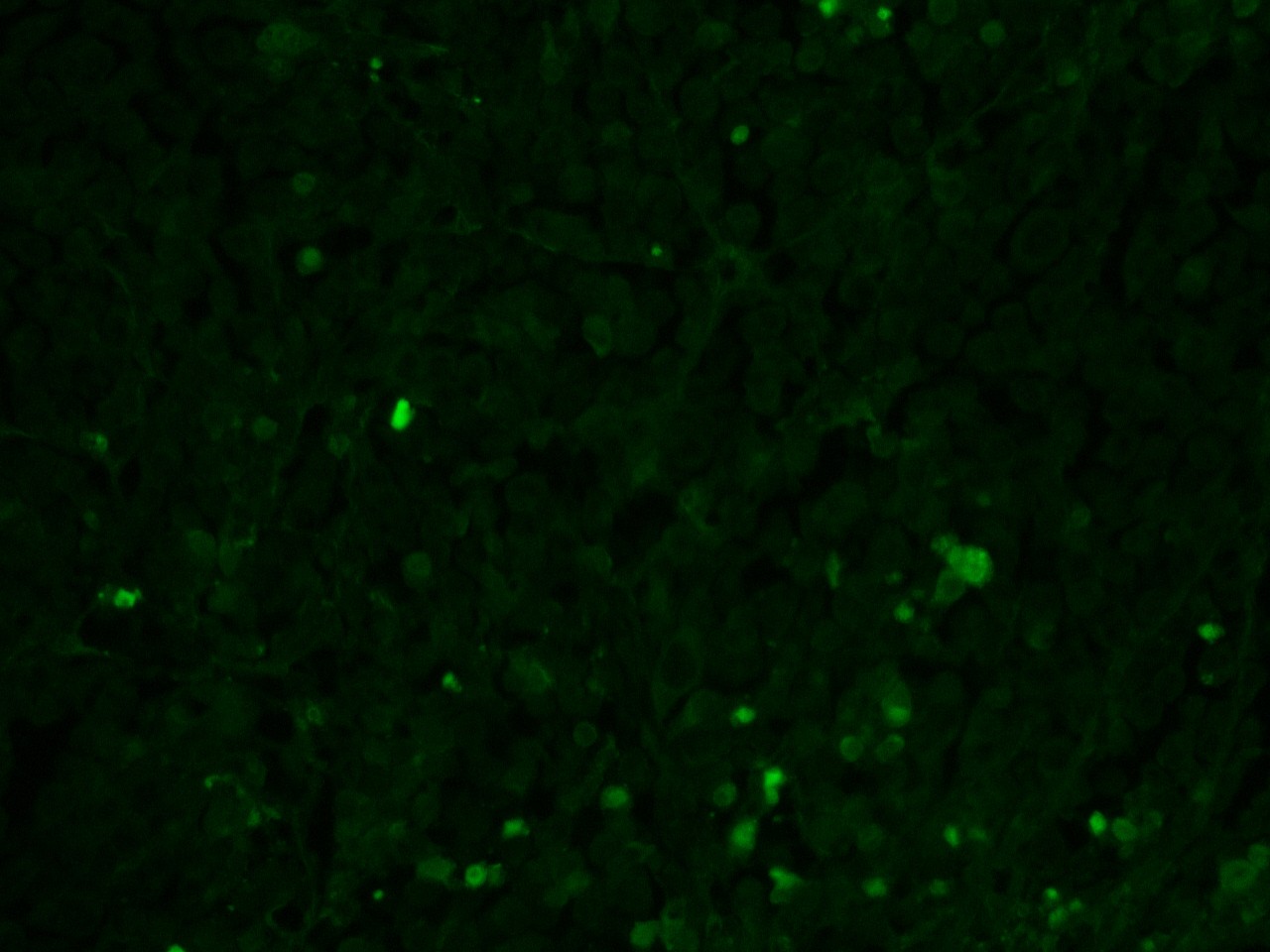

Supplement: Supplementary file 1 [file DataSheet1.ZIP › Raw data/Ki67& TUNEL stain/TUNEL/0 mgkg-400X-1.jpg]

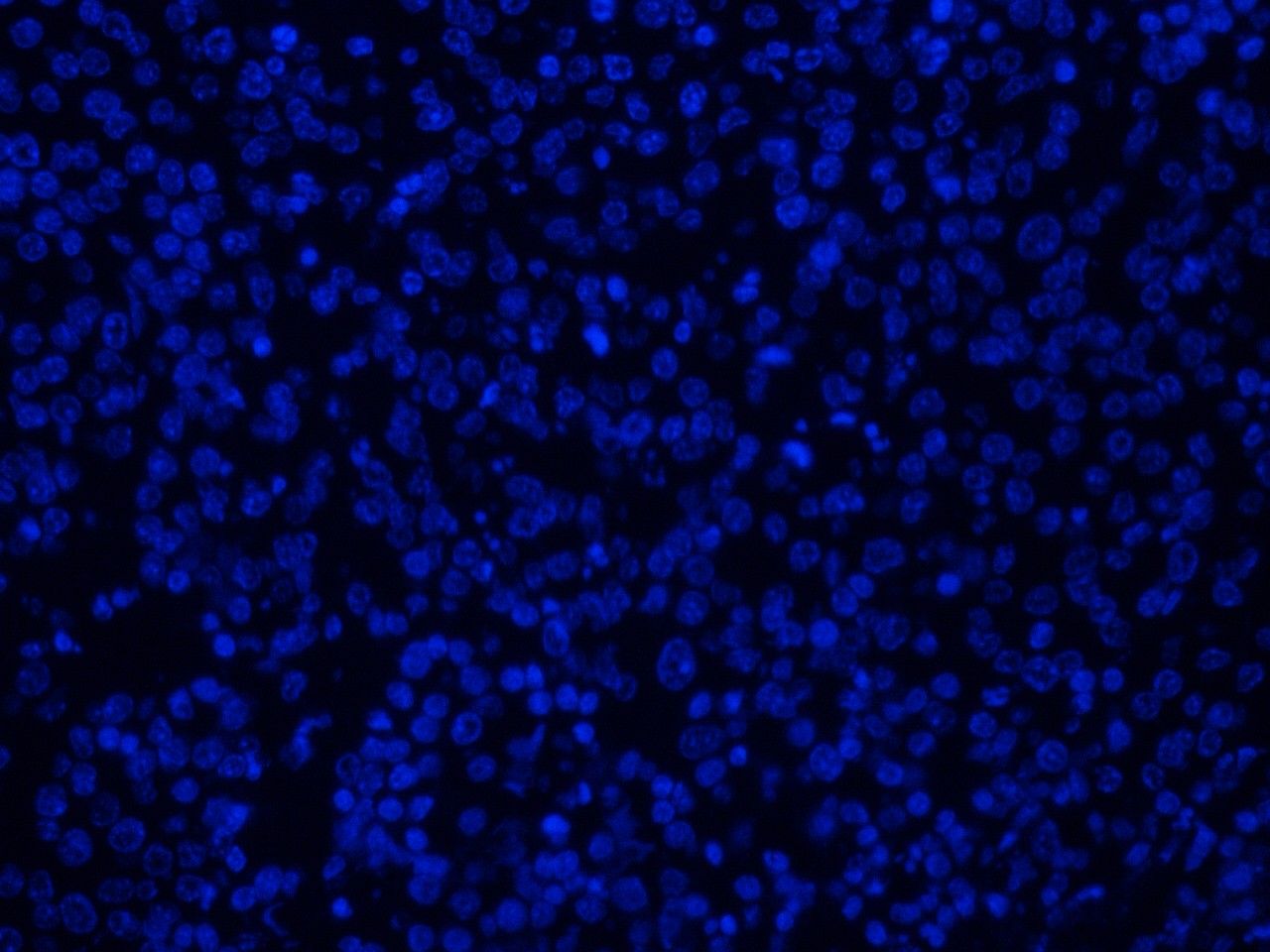

Supplement: Supplementary file 1 [file DataSheet1.ZIP › Raw data/Ki67& TUNEL stain/TUNEL/0 mgkg-400X-2.jpg]

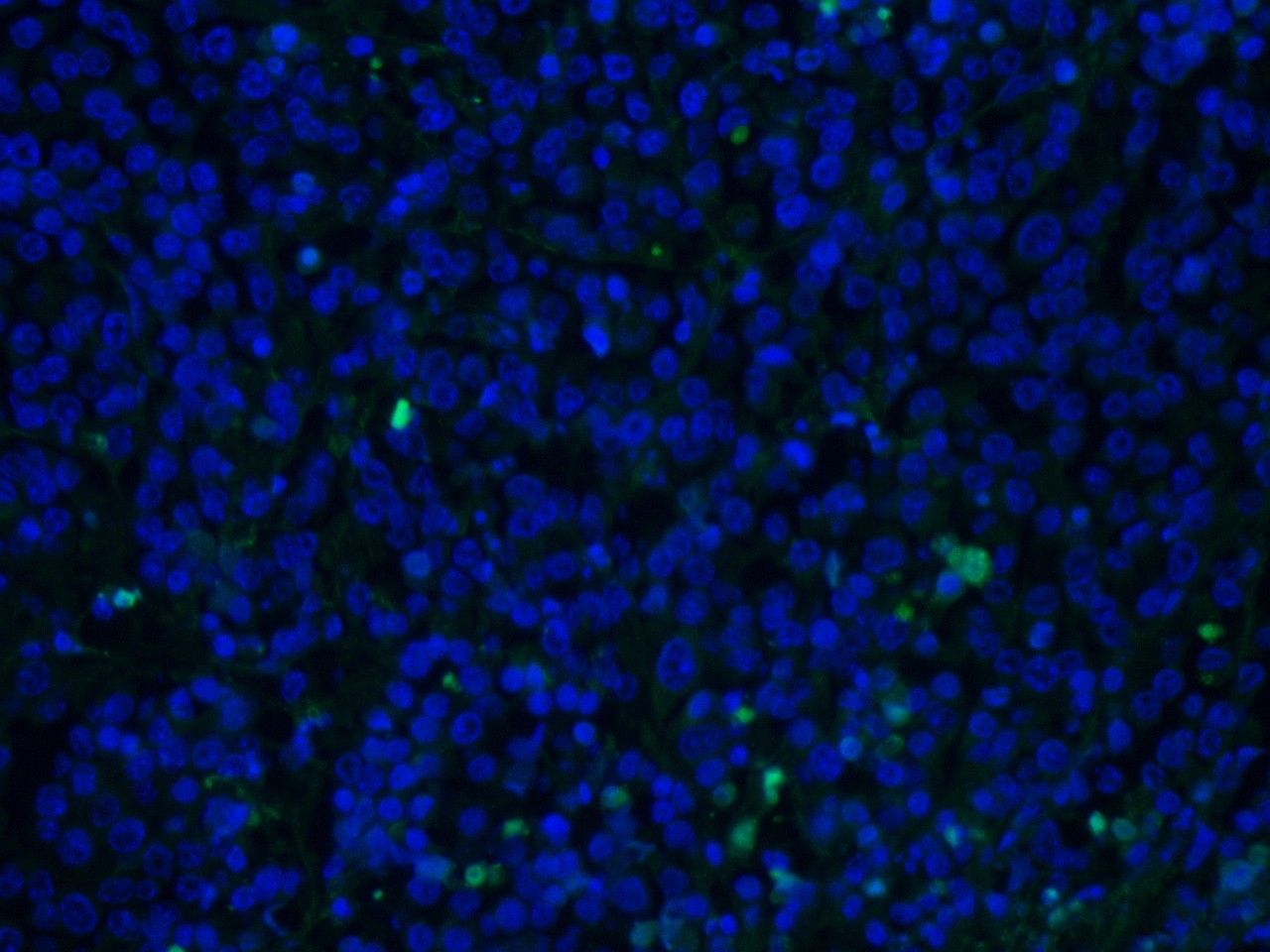

Supplement: Supplementary file 1 [file DataSheet1.ZIP › Raw data/Ki67& TUNEL stain/TUNEL/0 mgkg-400X.jpg]

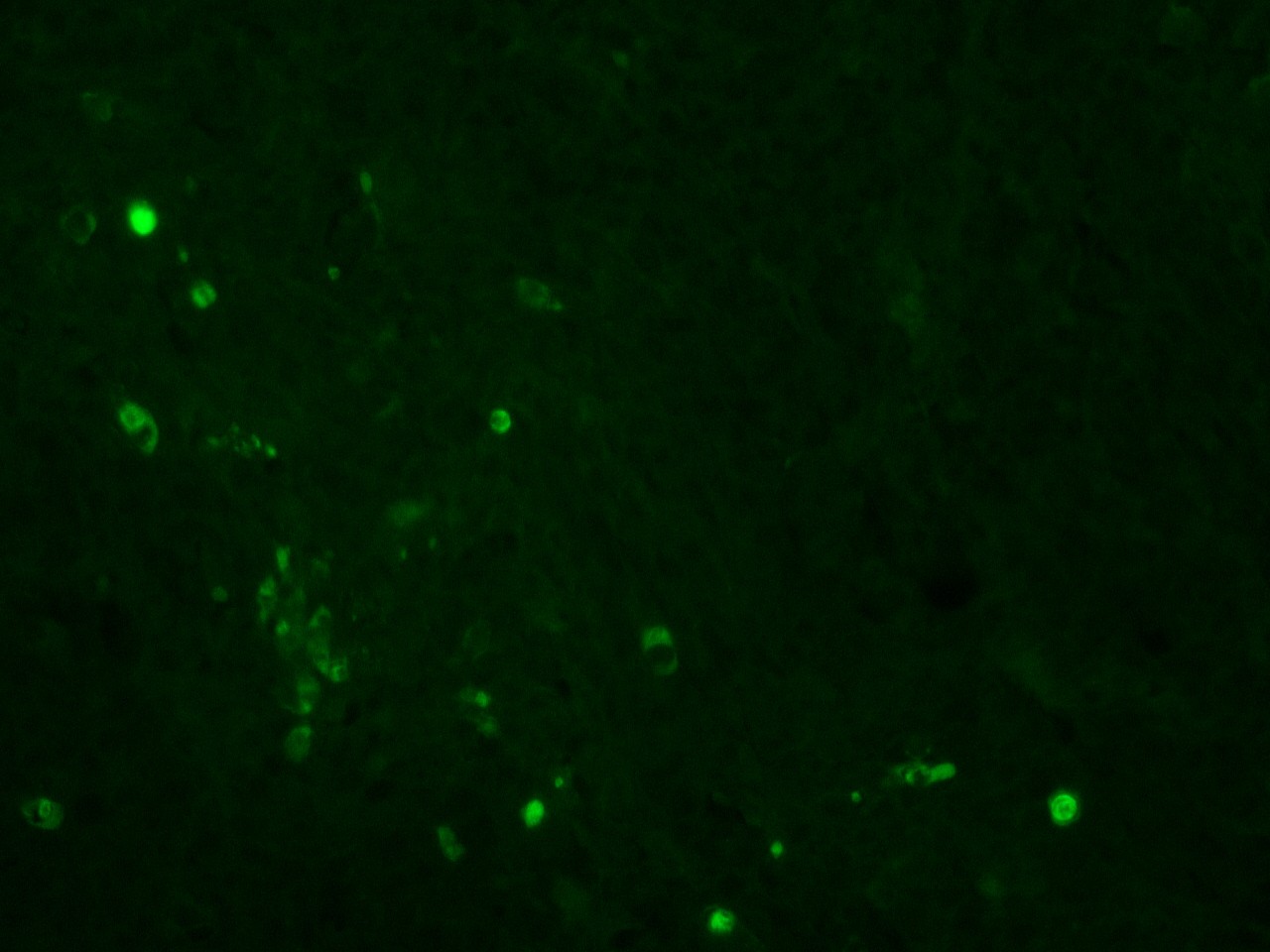

Supplement: Supplementary file 1 [file DataSheet1.ZIP › Raw data/Ki67& TUNEL stain/TUNEL/10mgkg-400X-1.jpg]

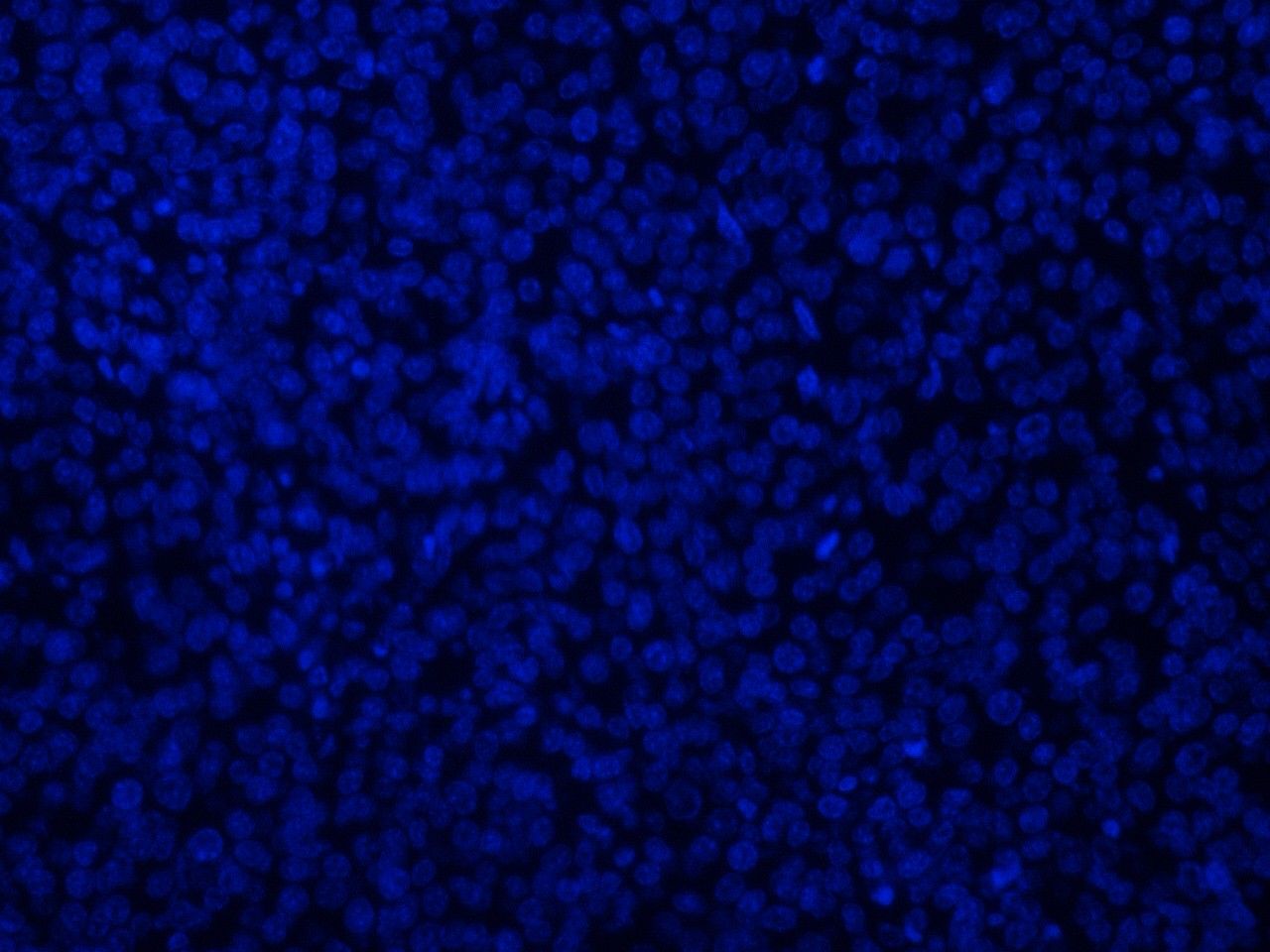

Supplement: Supplementary file 1 [file DataSheet1.ZIP › Raw data/Ki67& TUNEL stain/TUNEL/10mgkg-400X-2.jpg]

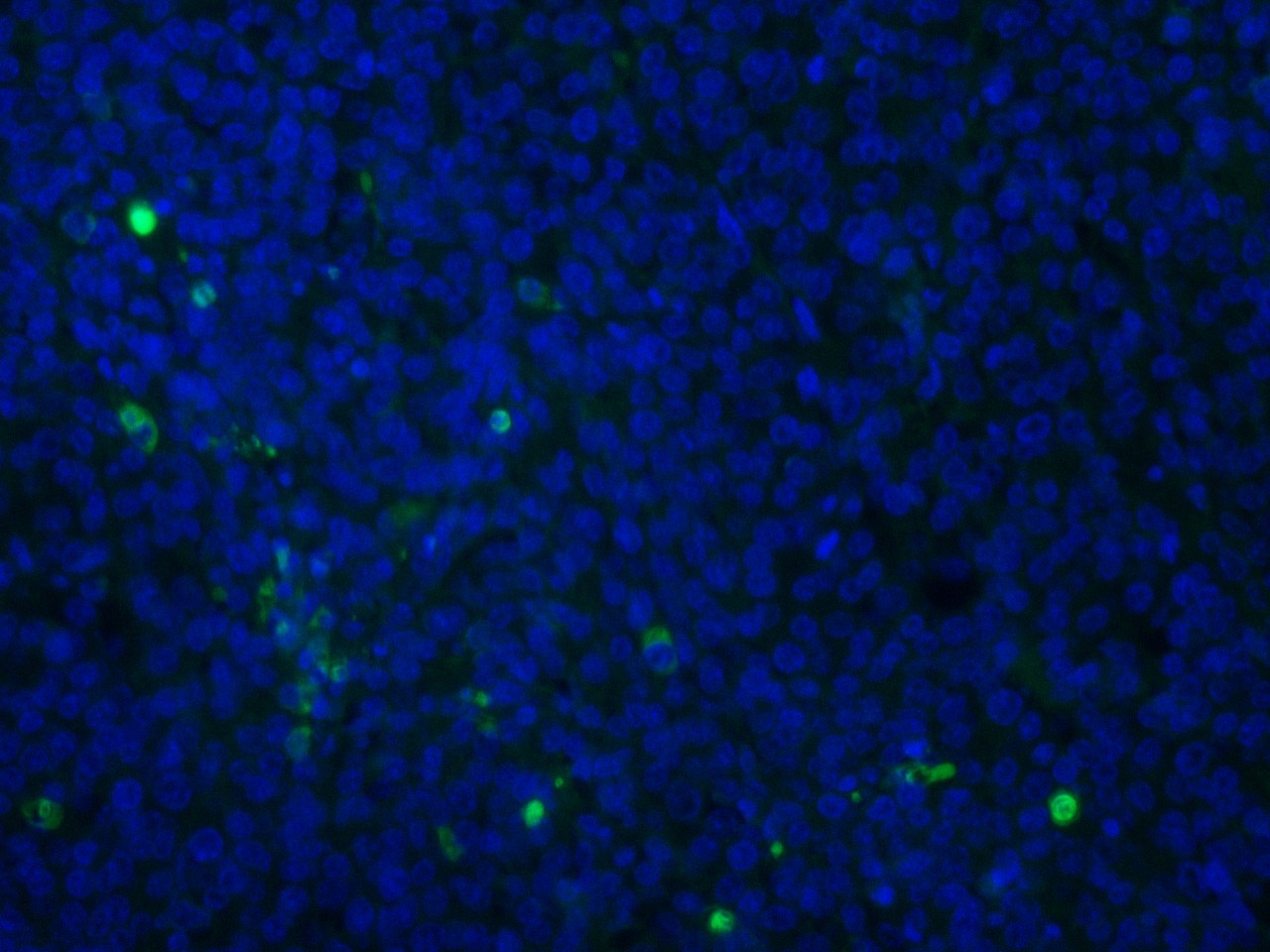

Supplement: Supplementary file 1 [file DataSheet1.ZIP › Raw data/Ki67& TUNEL stain/TUNEL/10mgkg-400X.jpg]

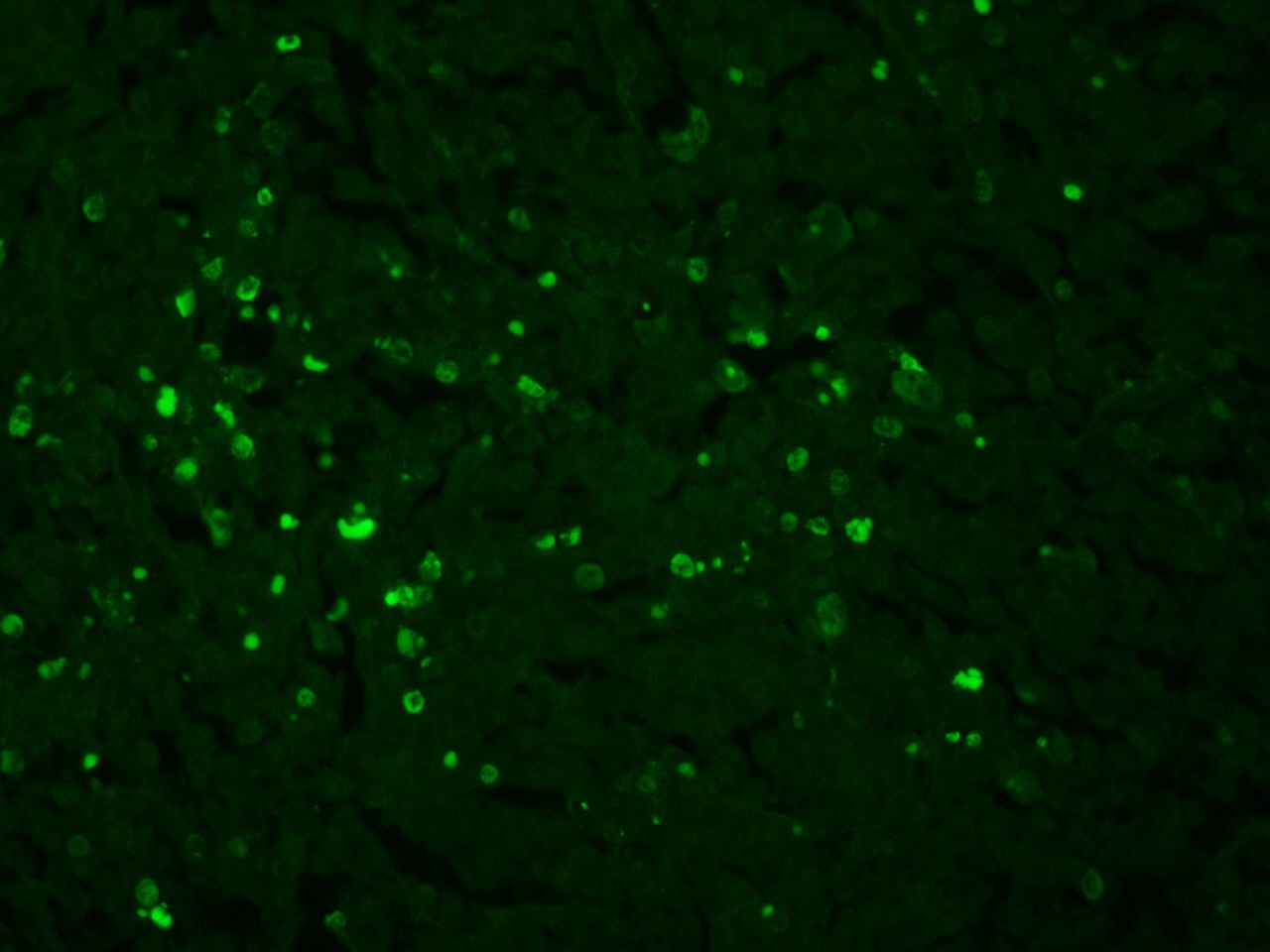

Supplement: Supplementary file 1 [file DataSheet1.ZIP › Raw data/Ki67& TUNEL stain/TUNEL/30mgkg-400X-1.jpg]

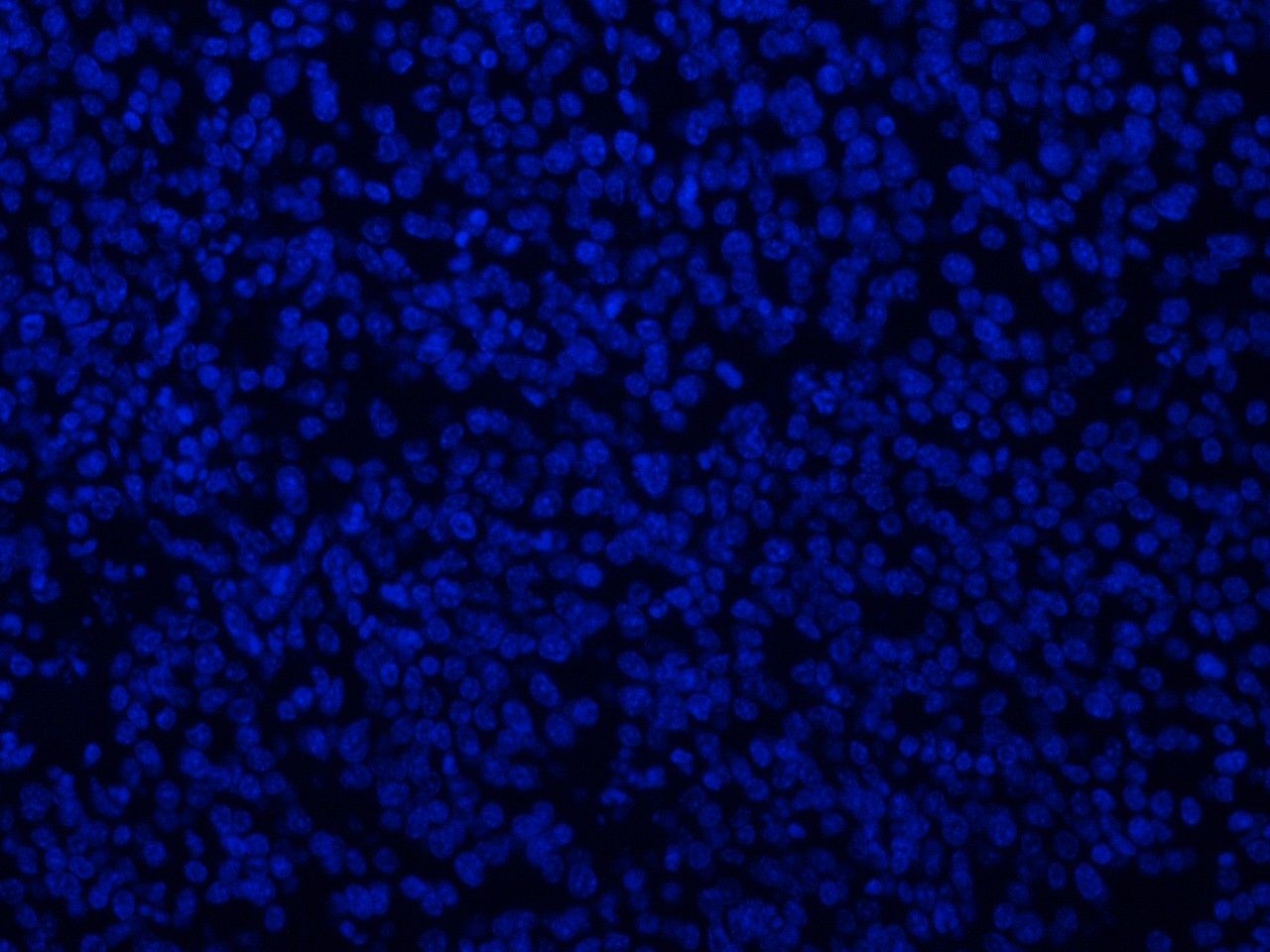

Supplement: Supplementary file 1 [file DataSheet1.ZIP › Raw data/Ki67& TUNEL stain/TUNEL/30mgkg-400X-2.jpg]

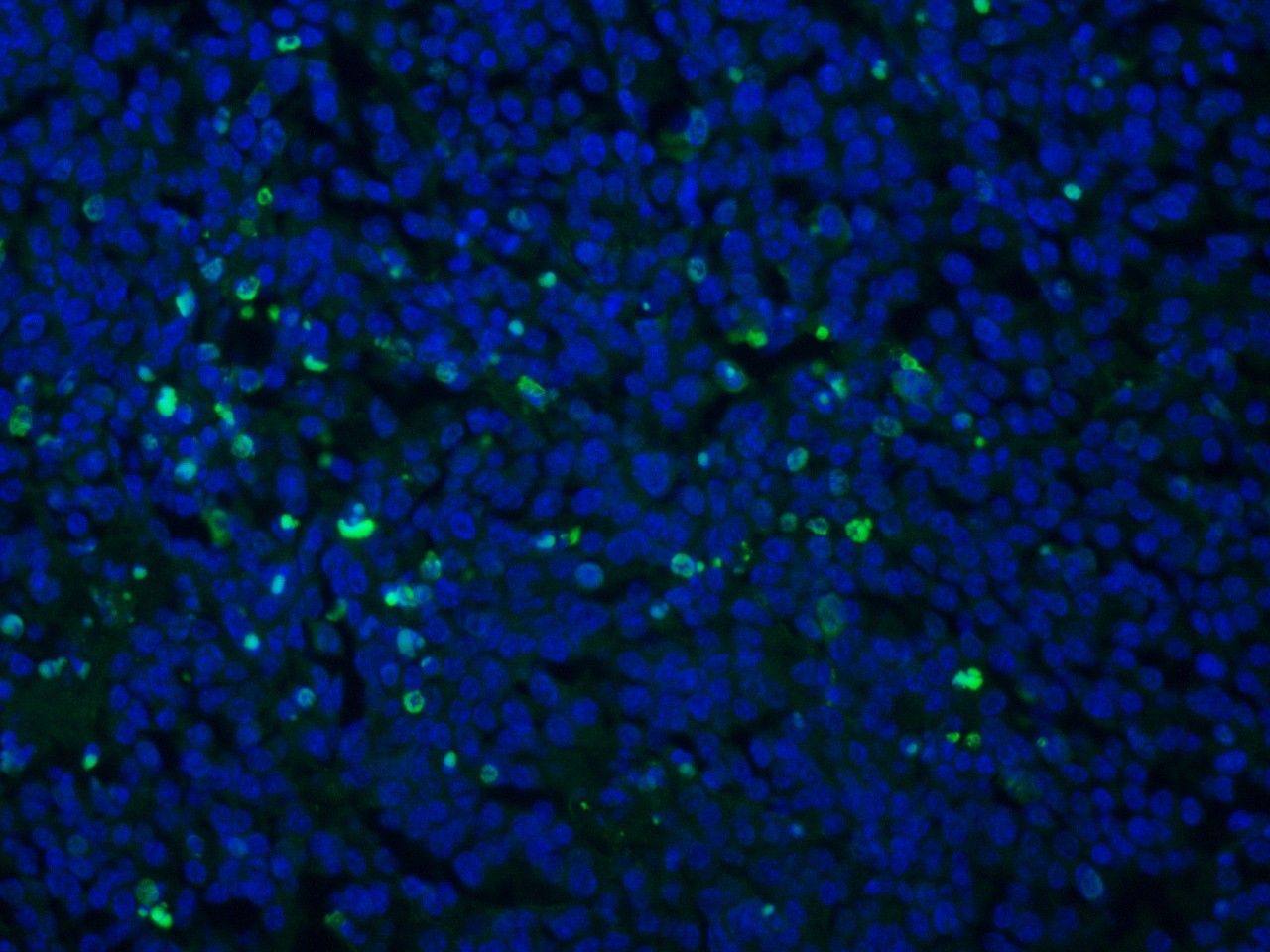

Supplement: Supplementary file 1 [file DataSheet1.ZIP › Raw data/Ki67& TUNEL stain/TUNEL/30mgkg-400X.jpg]

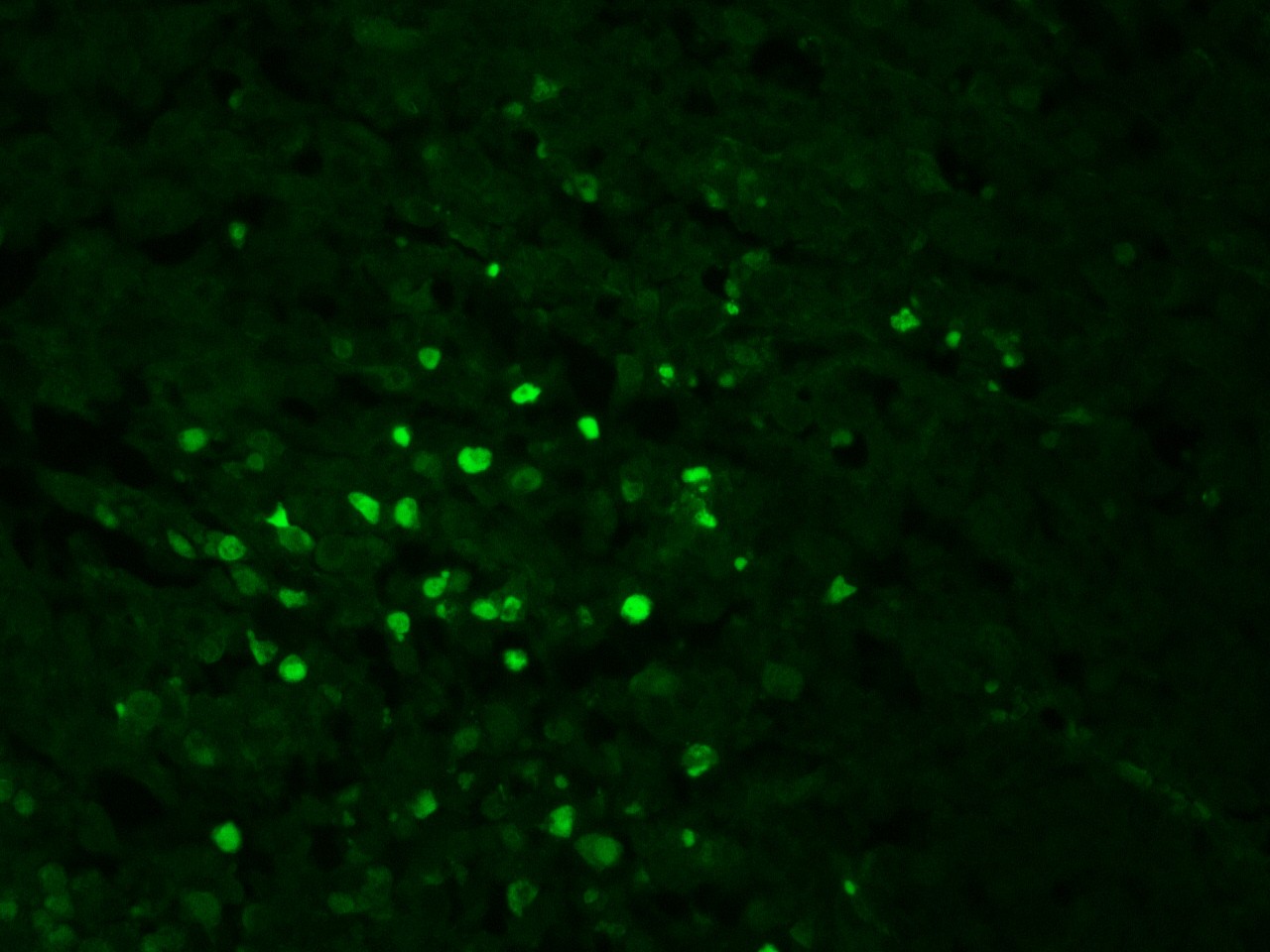

Supplement: Supplementary file 1 [file DataSheet1.ZIP › Raw data/Ki67& TUNEL stain/TUNEL/60mgkg-400X-1.jpg]

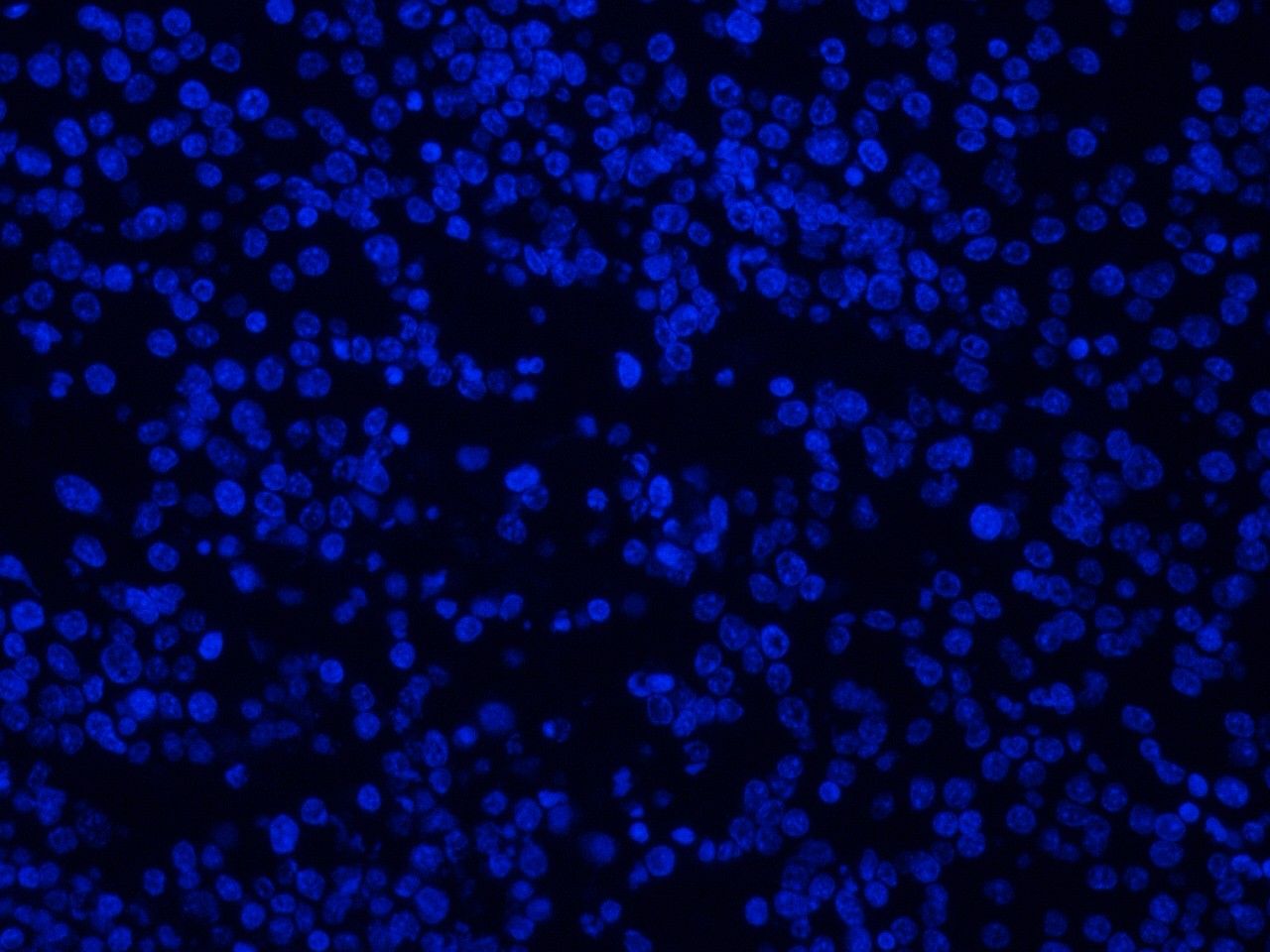

Supplement: Supplementary file 1 [file DataSheet1.ZIP › Raw data/Ki67& TUNEL stain/TUNEL/60mgkg-400X-2.jpg]

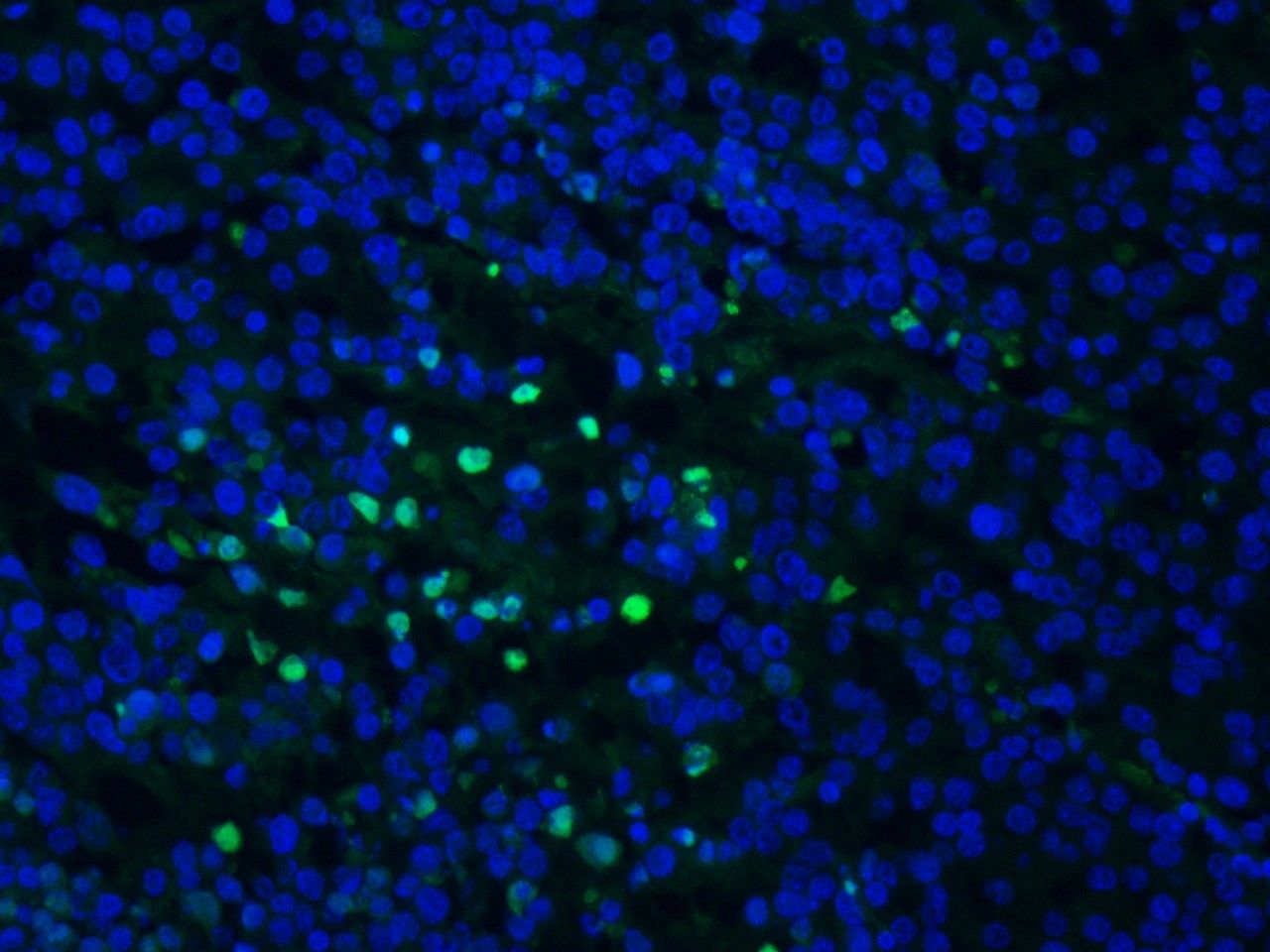

Supplement: Supplementary file 1 [file DataSheet1.ZIP › Raw data/Ki67& TUNEL stain/TUNEL/60mgkg-400X.jpg]

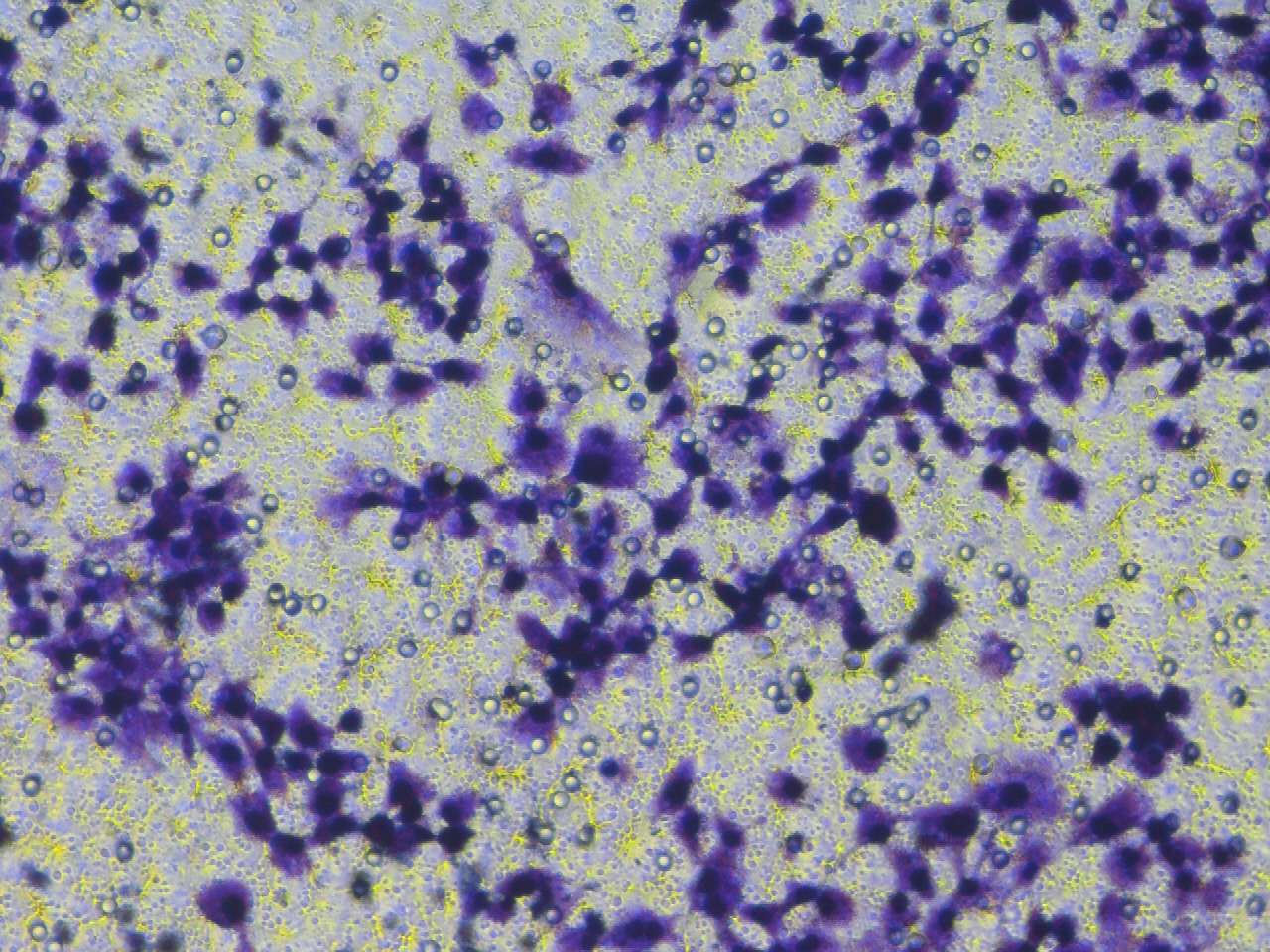

Supplement: Supplementary file 1 [file DataSheet1.ZIP › Raw data/migratory and invasion-SPSS&GraphPad7 statistics/0-200x.jpg]

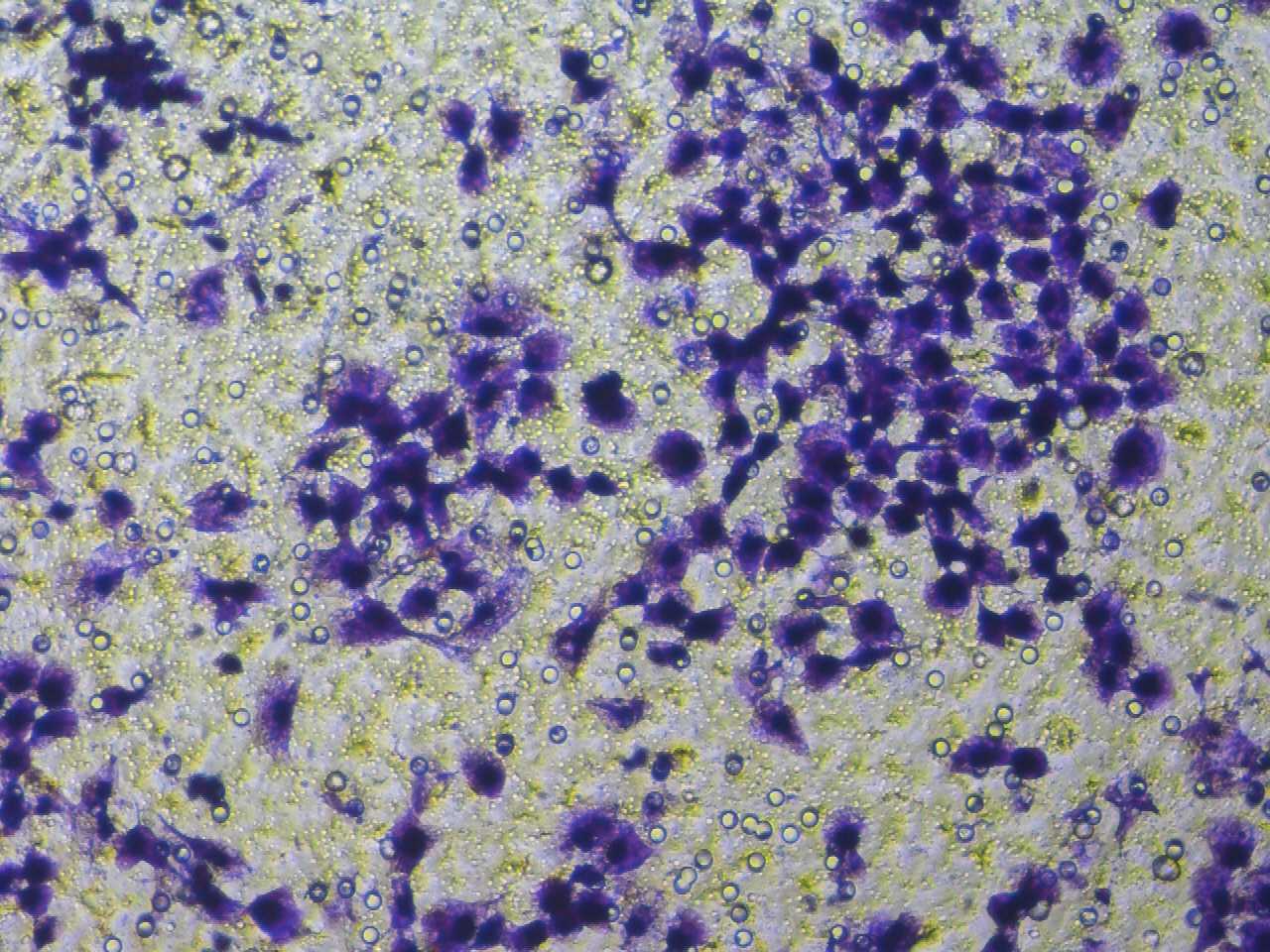

Supplement: Supplementary file 1 [file DataSheet1.ZIP › Raw data/migratory and invasion-SPSS&GraphPad7 statistics/0-200x1.jpg]

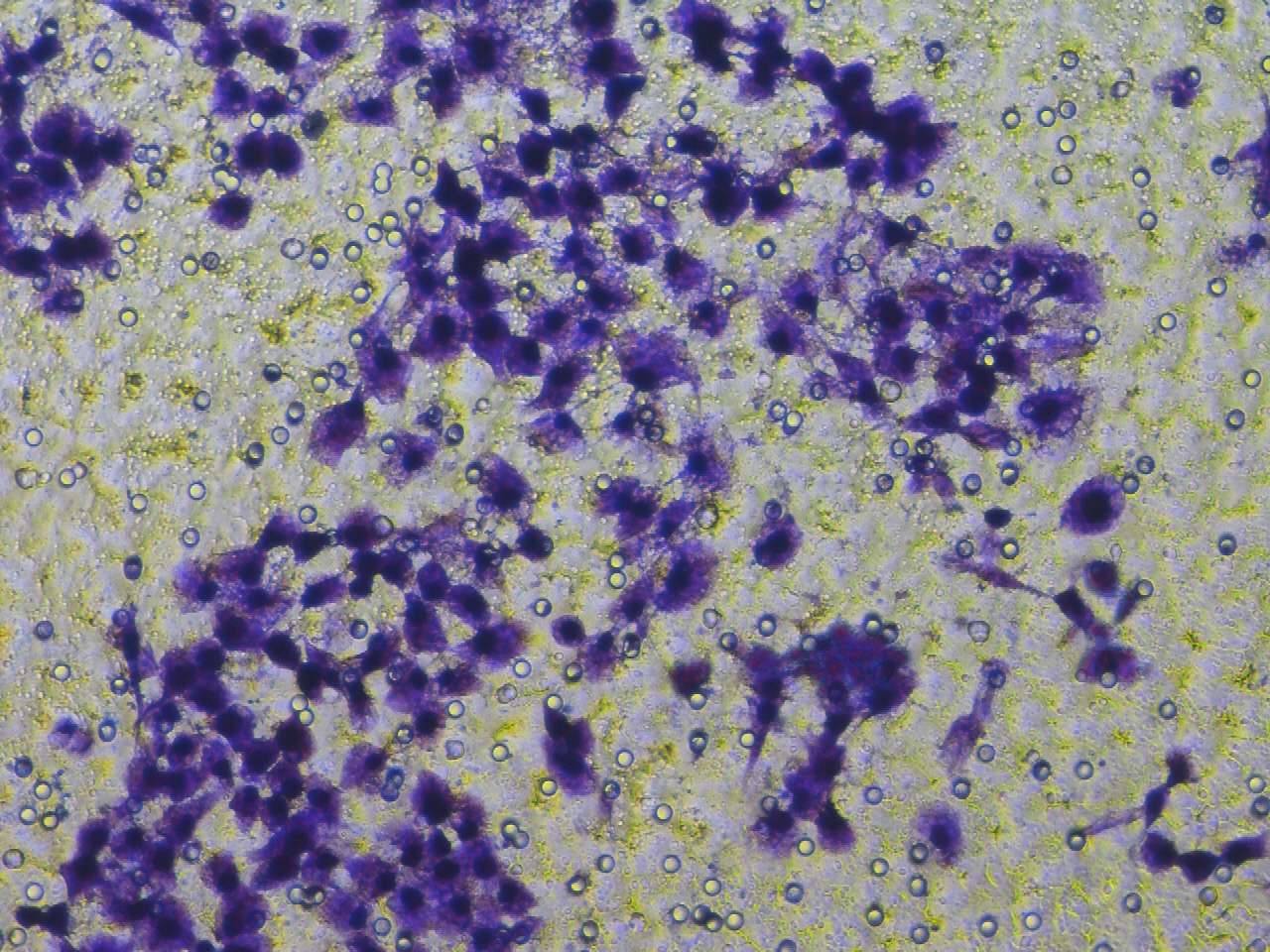

Supplement: Supplementary file 1 [file DataSheet1.ZIP › Raw data/migratory and invasion-SPSS&GraphPad7 statistics/0-200x2.jpg]

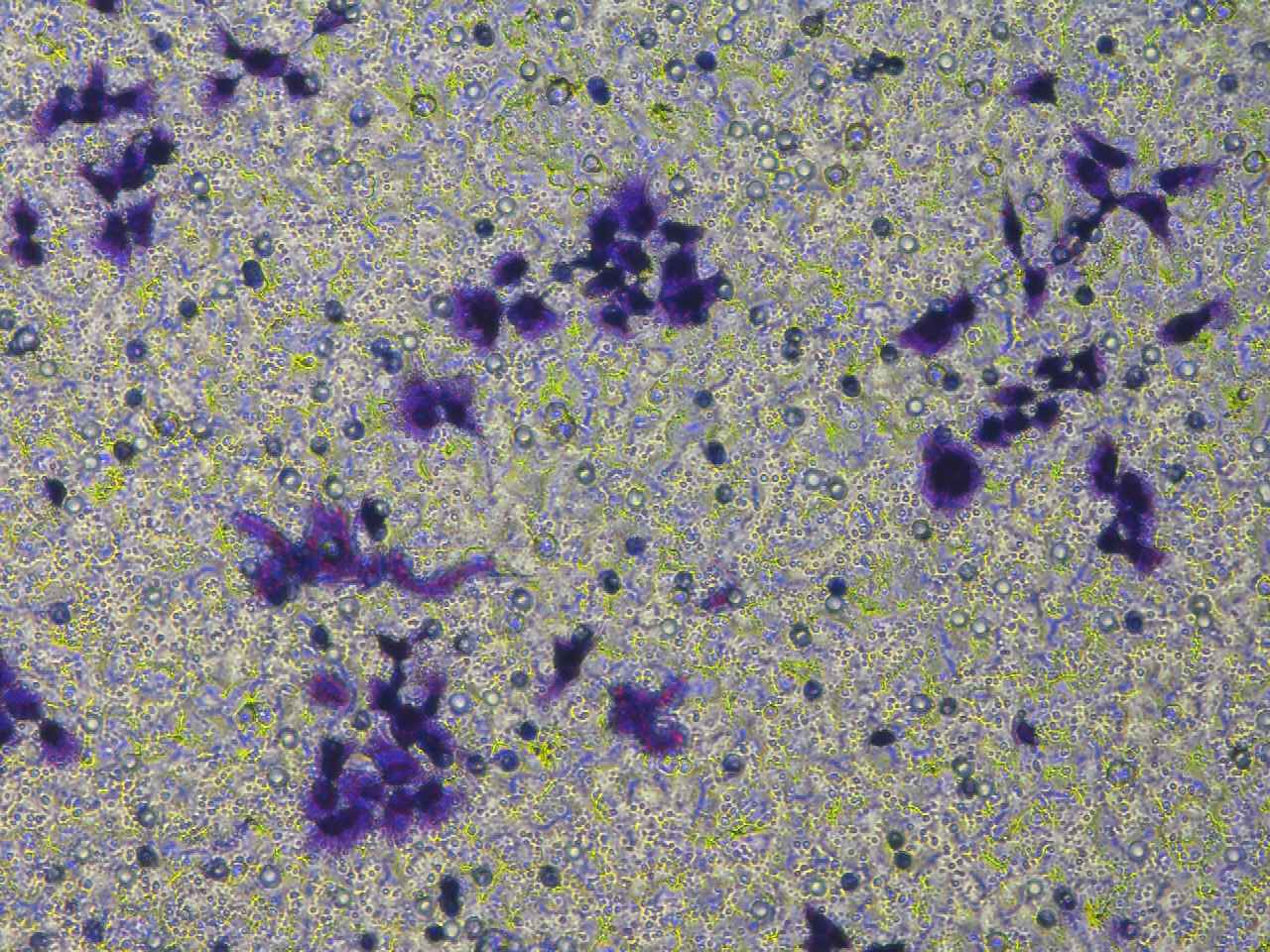

Supplement: Supplementary file 1 [file DataSheet1.ZIP › Raw data/migratory and invasion-SPSS&GraphPad7 statistics/1.25-200x.jpg]

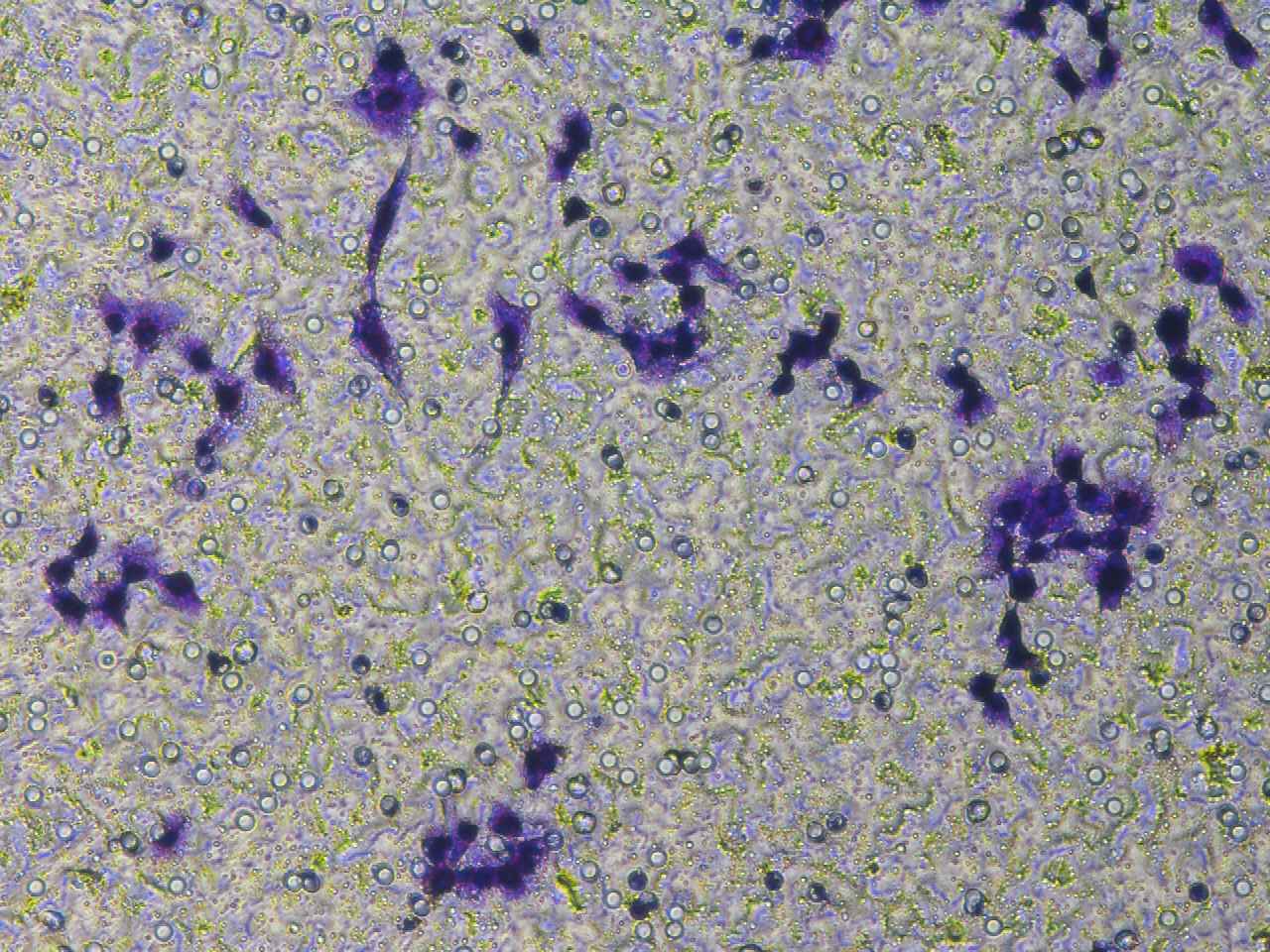

Supplement: Supplementary file 1 [file DataSheet1.ZIP › Raw data/migratory and invasion-SPSS&GraphPad7 statistics/1.25-200x1.jpg]

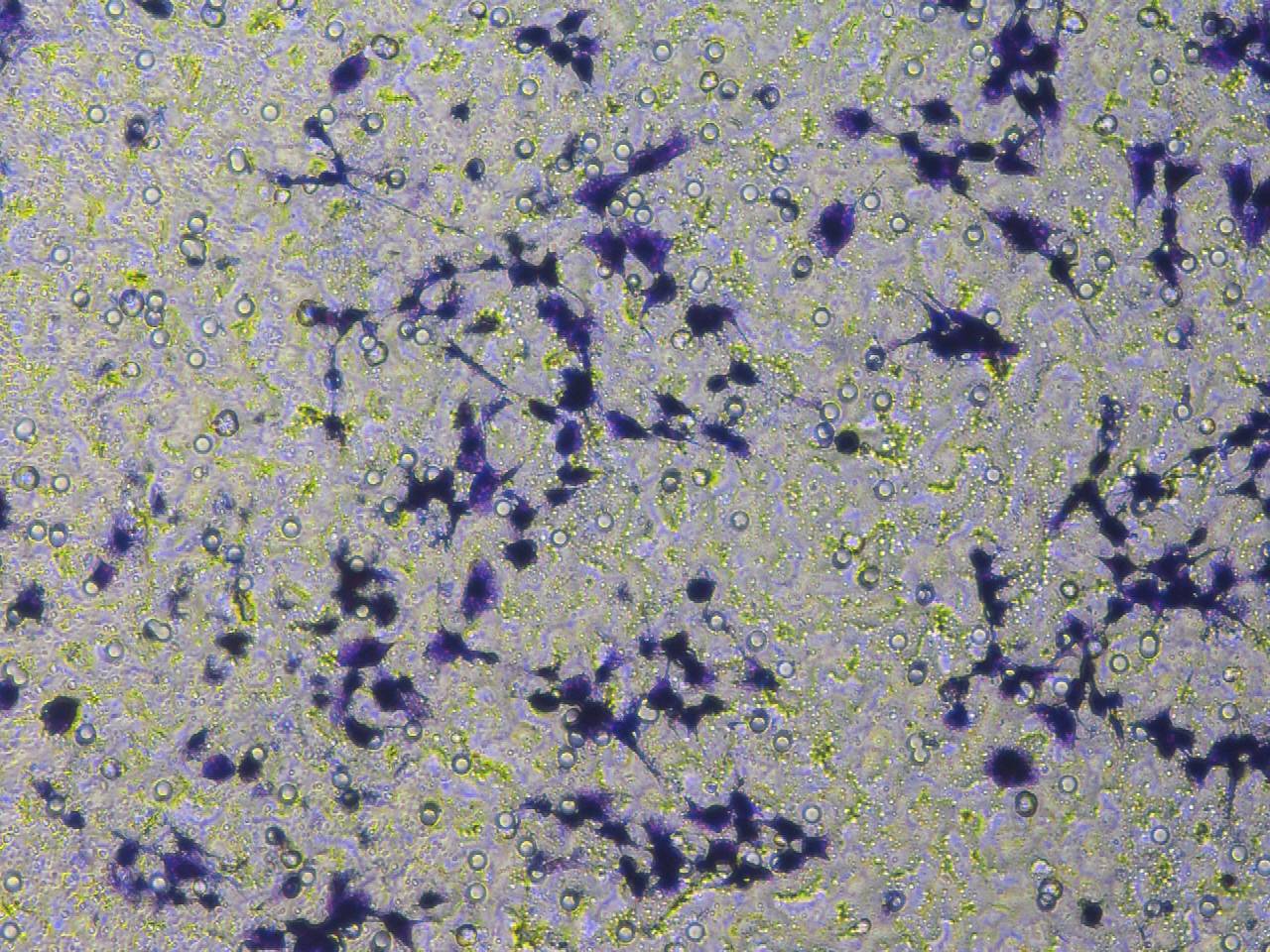

Supplement: Supplementary file 1 [file DataSheet1.ZIP › Raw data/migratory and invasion-SPSS&GraphPad7 statistics/1.25-200x4.jpg]

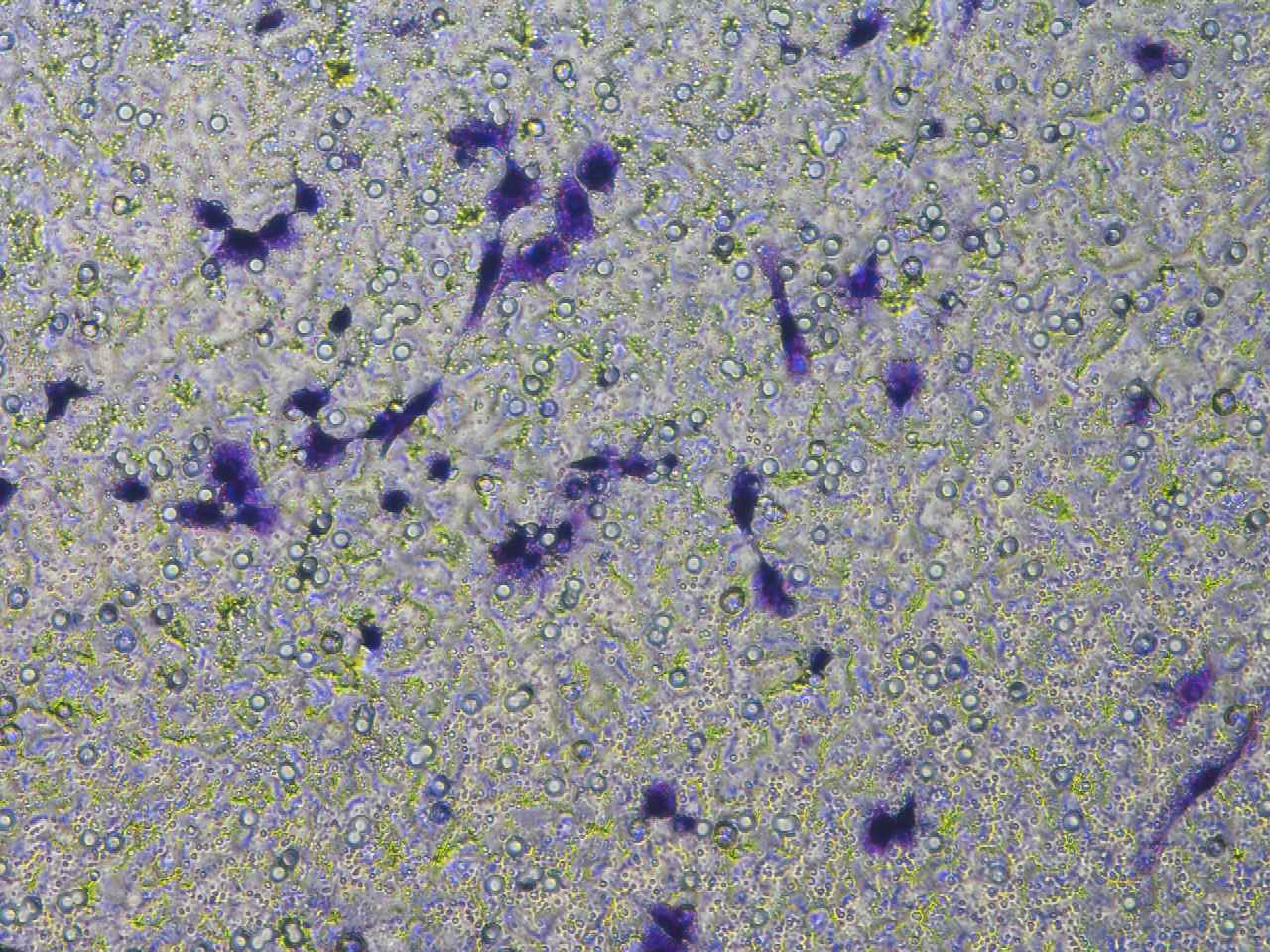

Supplement: Supplementary file 1 [file DataSheet1.ZIP › Raw data/migratory and invasion-SPSS&GraphPad7 statistics/2.5-200x.jpg]

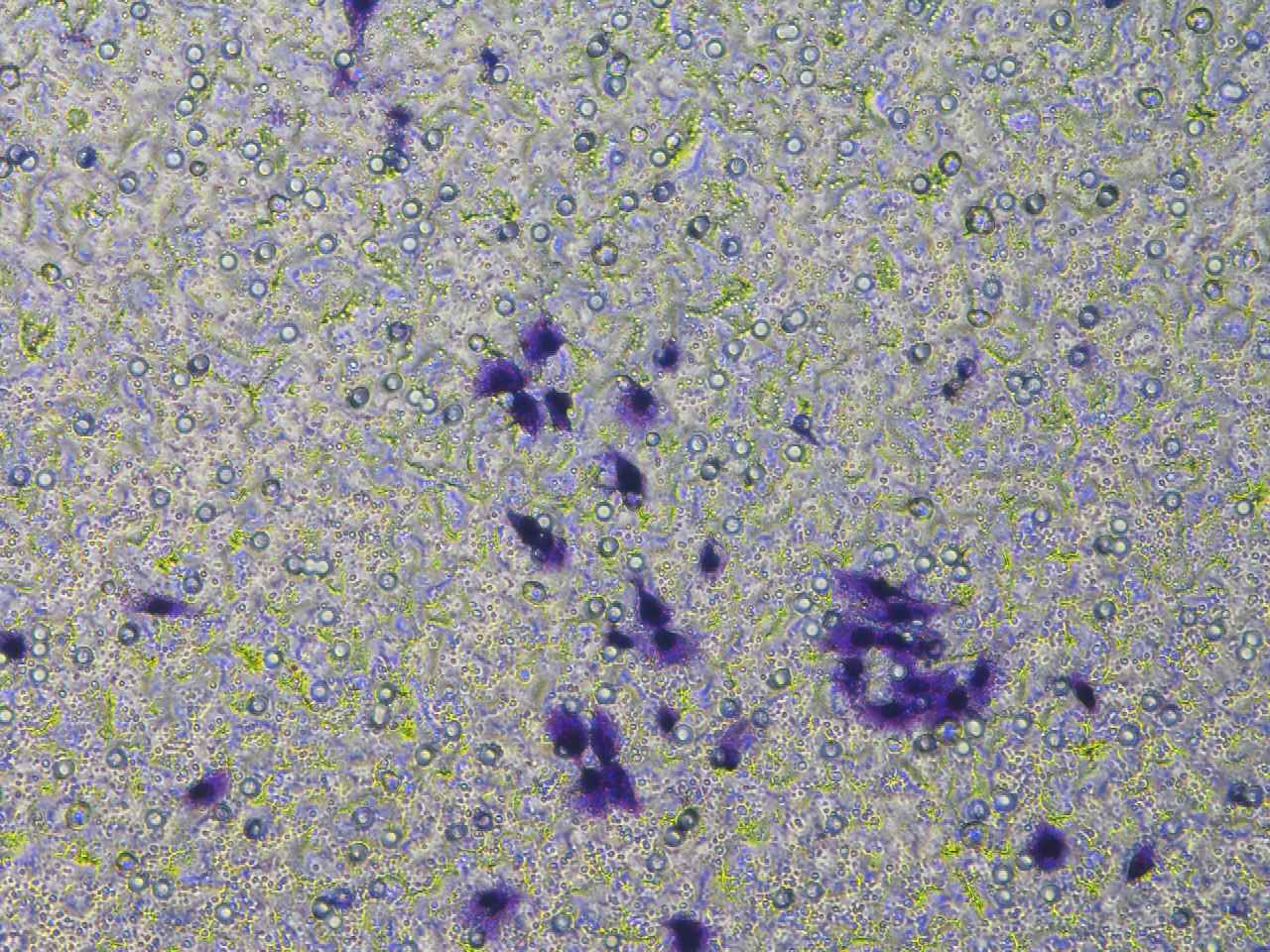

Supplement: Supplementary file 1 [file DataSheet1.ZIP › Raw data/migratory and invasion-SPSS&GraphPad7 statistics/2.5-200x2.jpg]

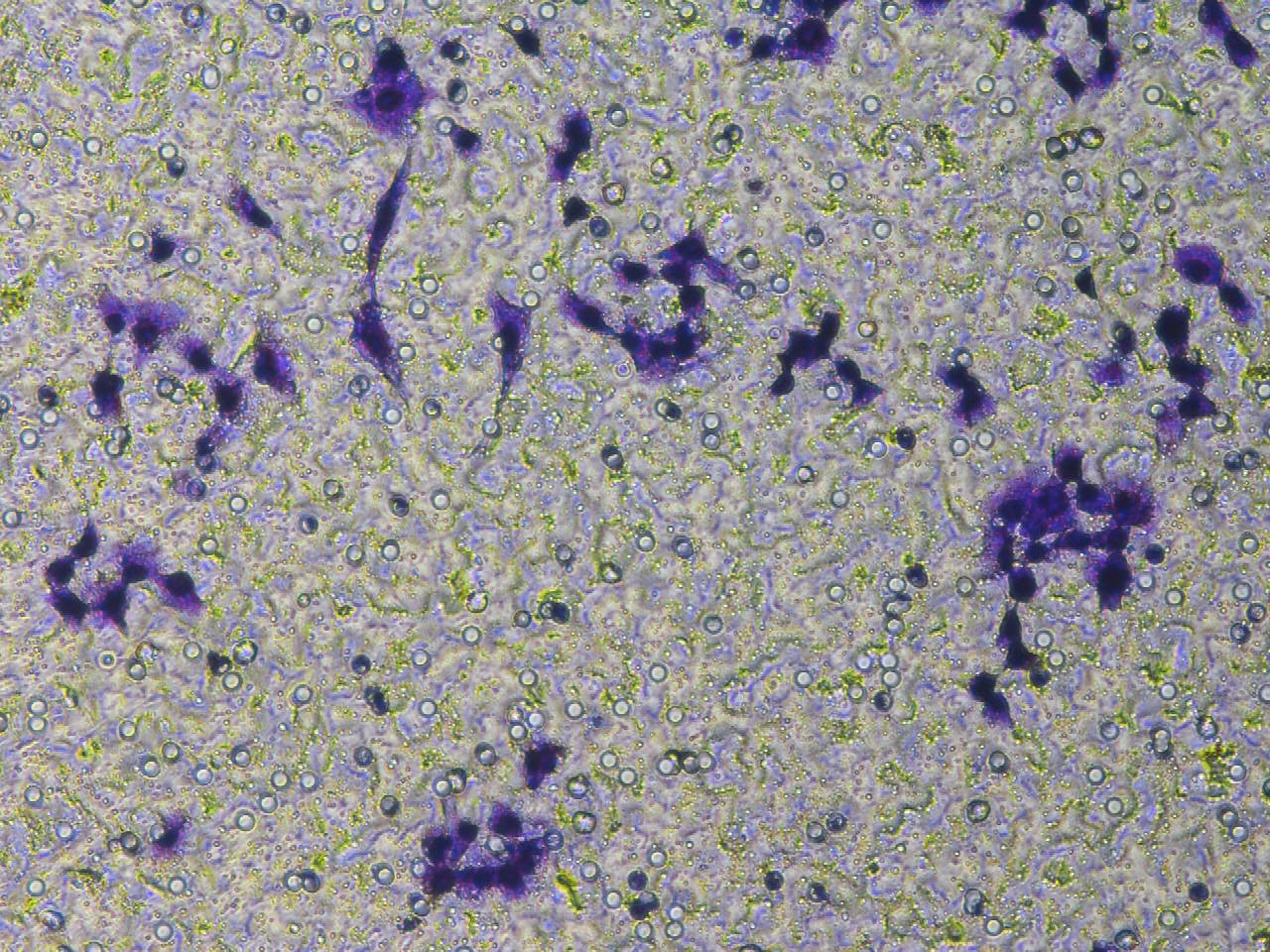

Supplement: Supplementary file 1 [file DataSheet1.ZIP › Raw data/migratory and invasion-SPSS&GraphPad7 statistics/2.5-200x4.jpg]

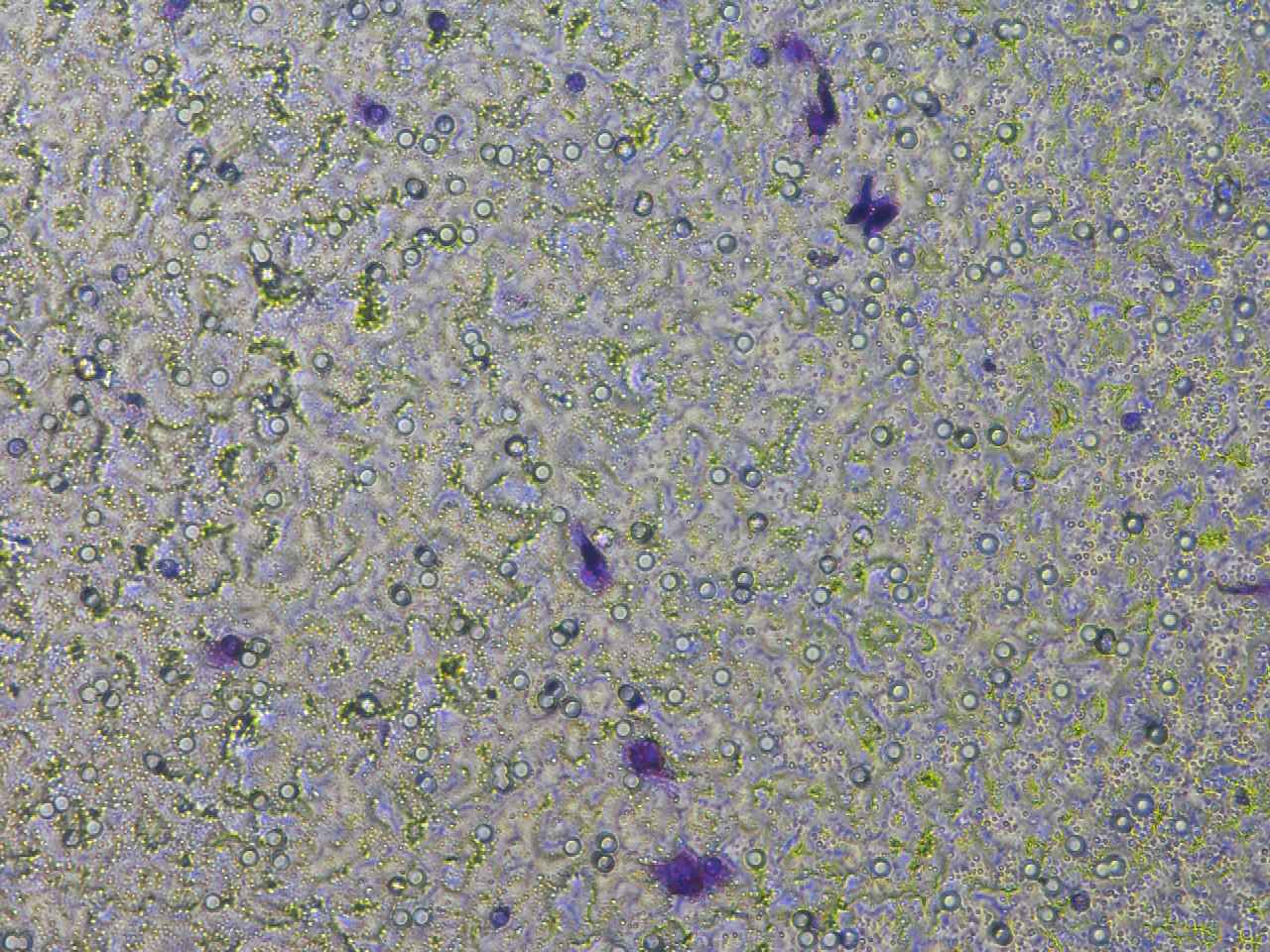

Supplement: Supplementary file 1 [file DataSheet1.ZIP › Raw data/migratory and invasion-SPSS&GraphPad7 statistics/5-200x1.jpg]

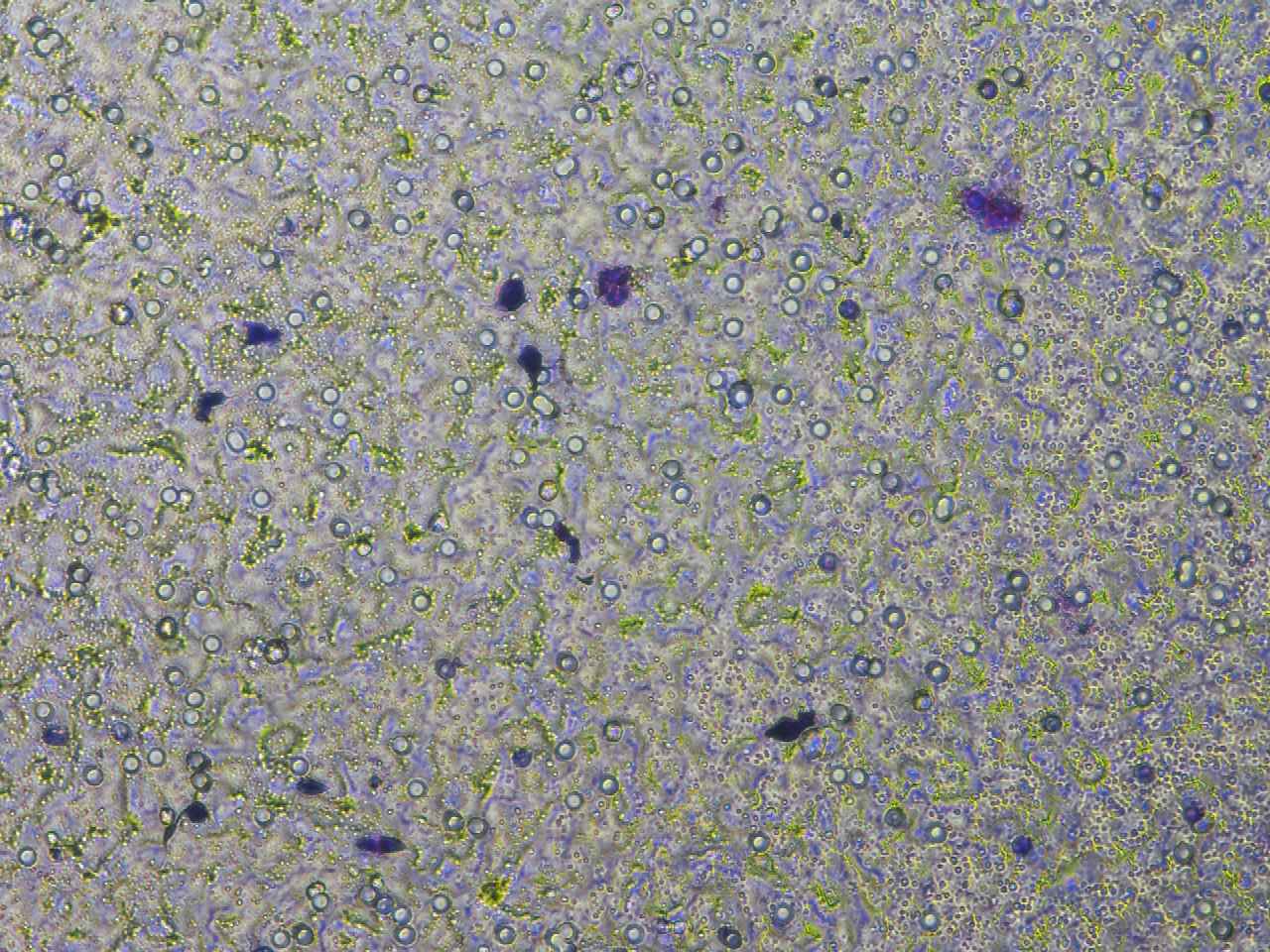

Supplement: Supplementary file 1 [file DataSheet1.ZIP › Raw data/migratory and invasion-SPSS&GraphPad7 statistics/5-200x2.jpg]

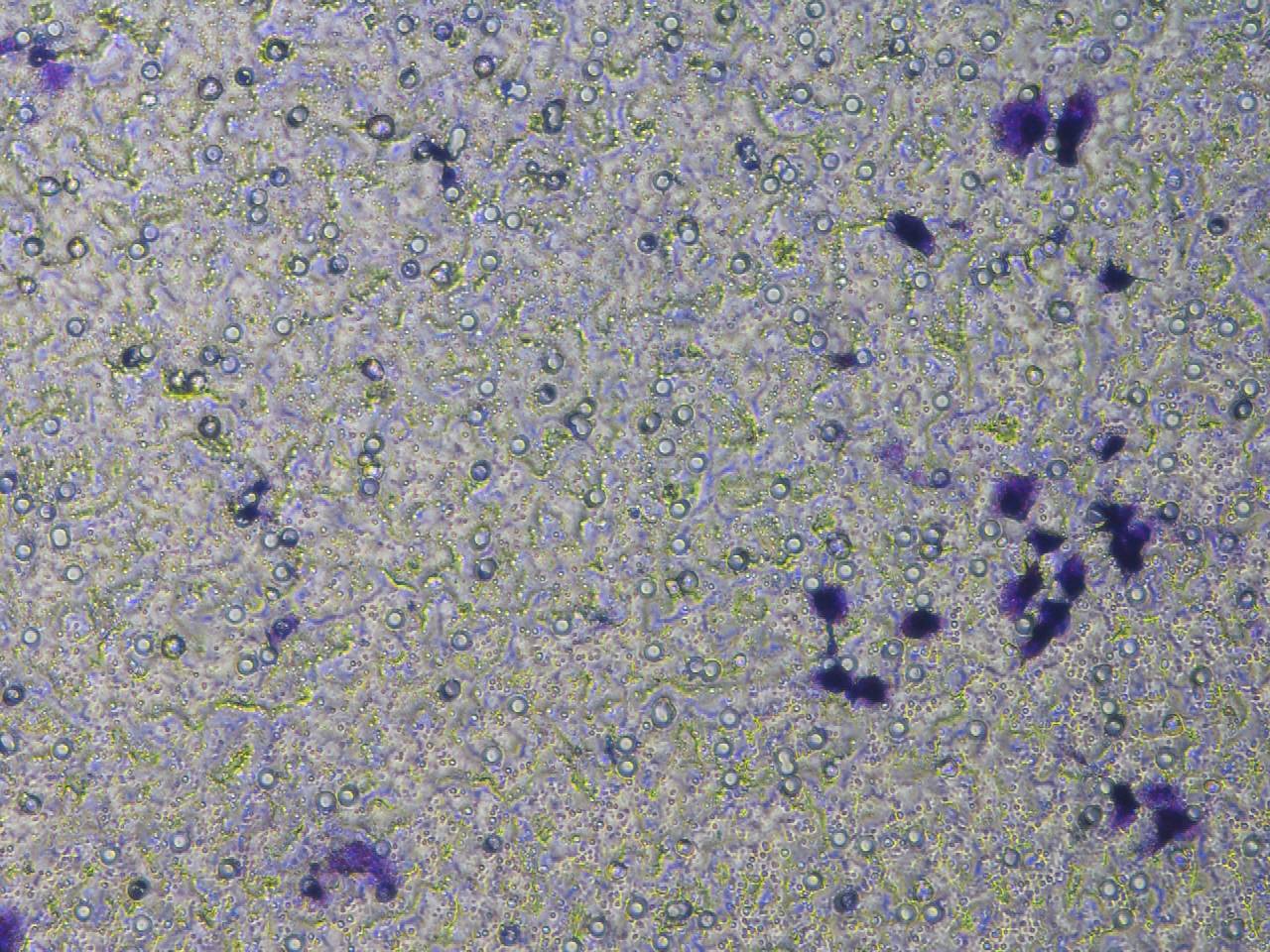

Supplement: Supplementary file 1 [file DataSheet1.ZIP › Raw data/migratory and invasion-SPSS&GraphPad7 statistics/5-200x4.jpg]

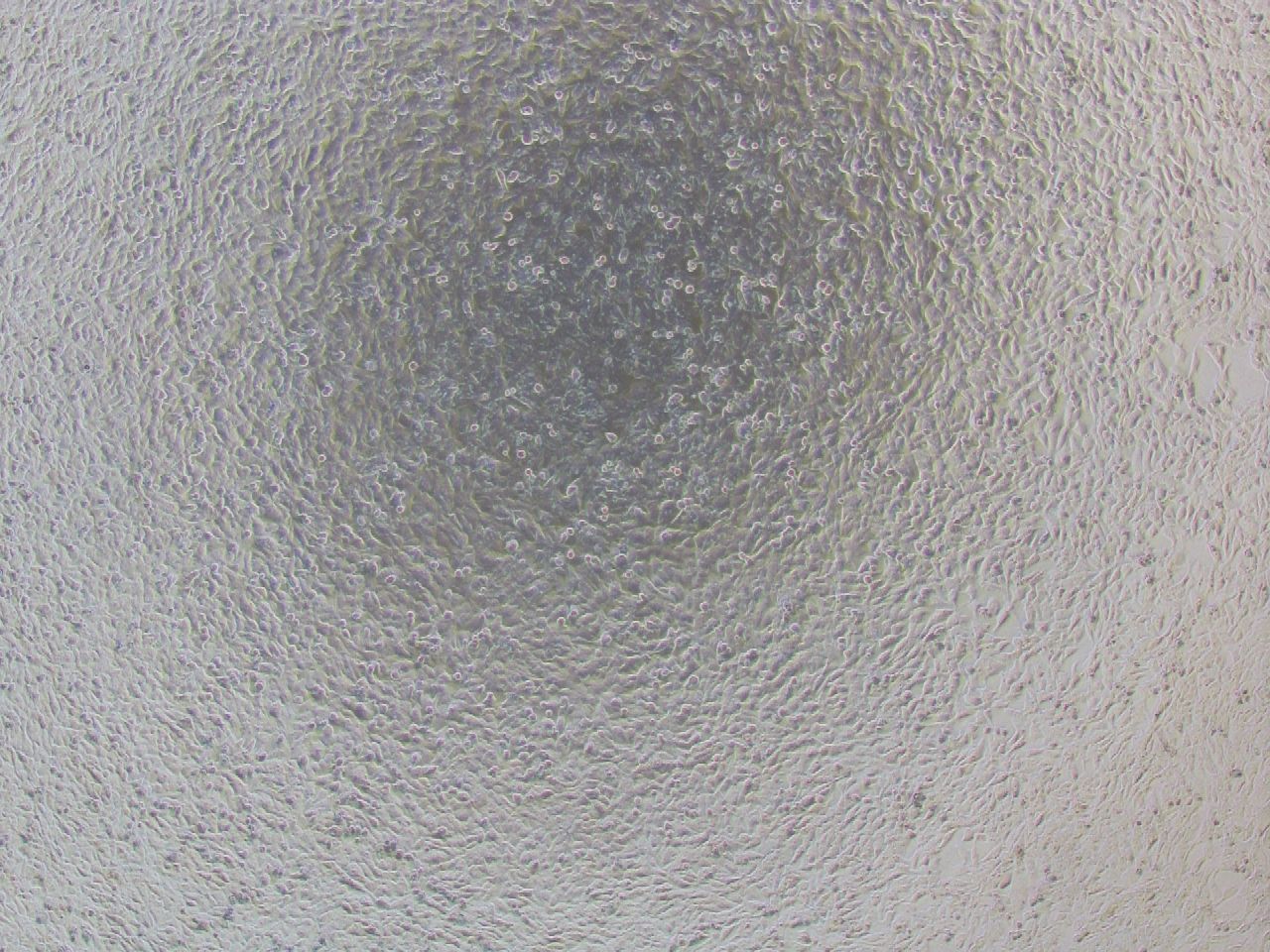

Supplement: Supplementary file 1 [file DataSheet1.ZIP › Raw data/morphology of RM-1 cells/0-1.jpg]

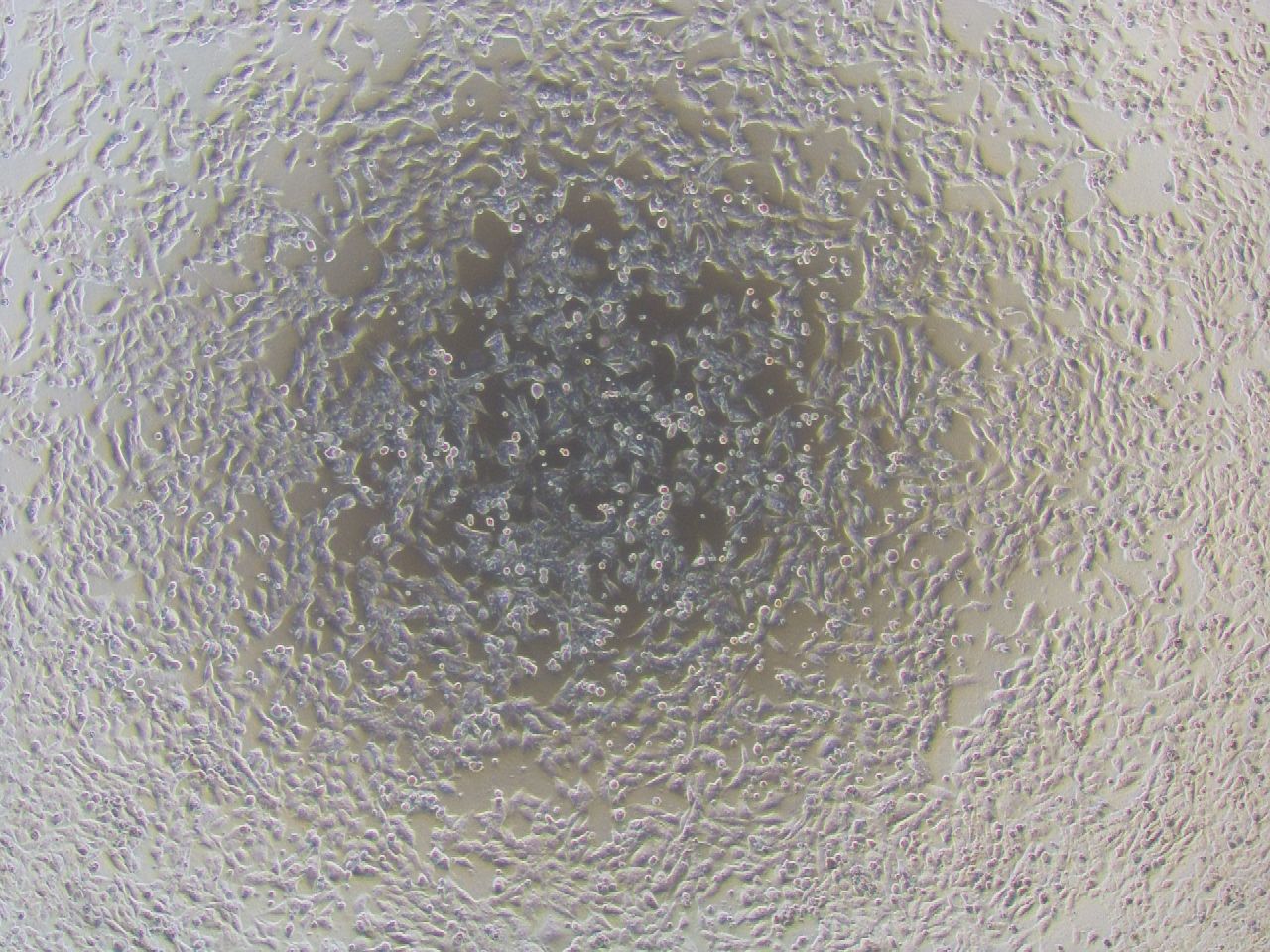

Supplement: Supplementary file 1 [file DataSheet1.ZIP › Raw data/morphology of RM-1 cells/1.25-1.jpg]

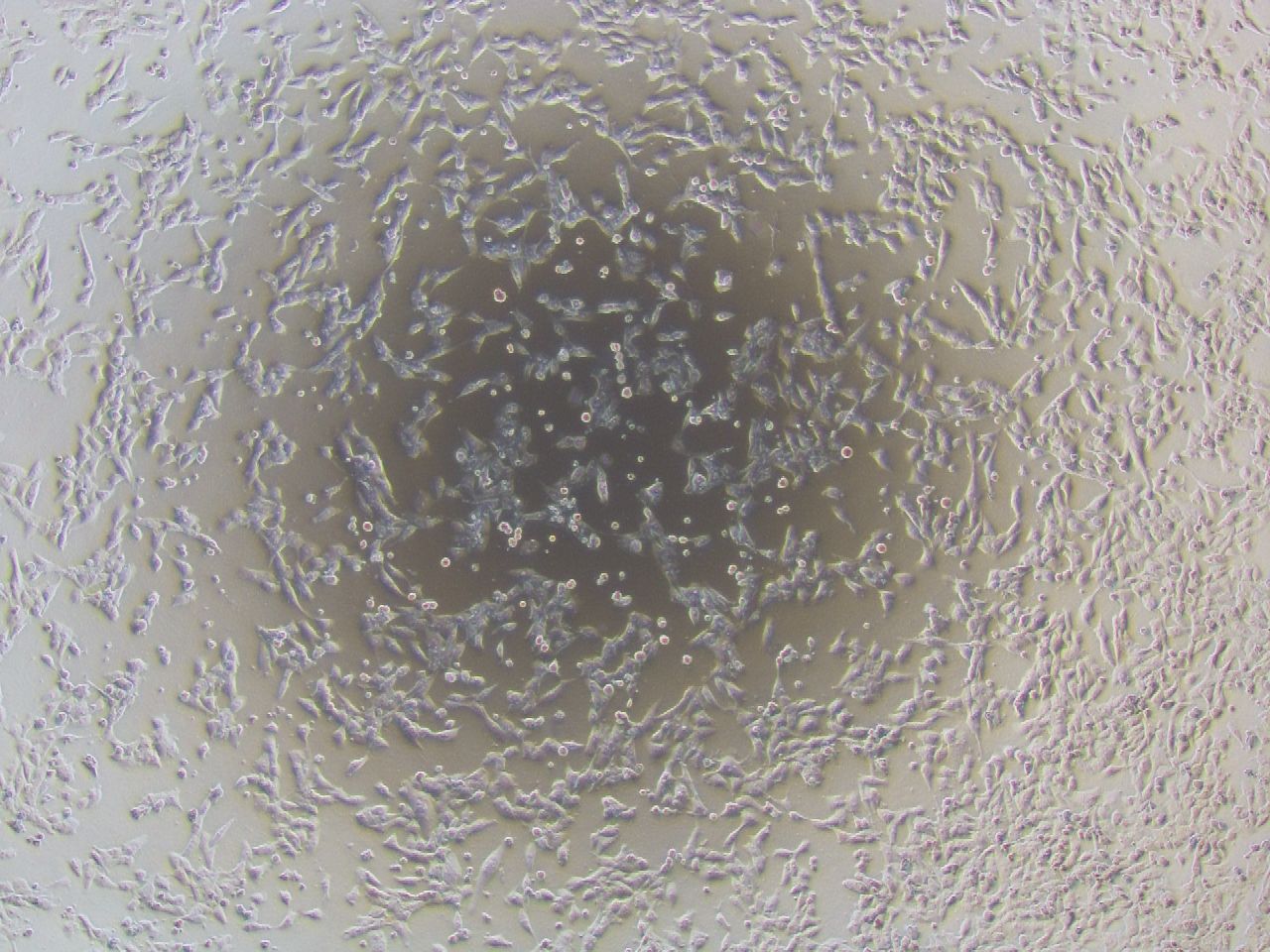

Supplement: Supplementary file 1 [file DataSheet1.ZIP › Raw data/morphology of RM-1 cells/2.5-4.jpg]

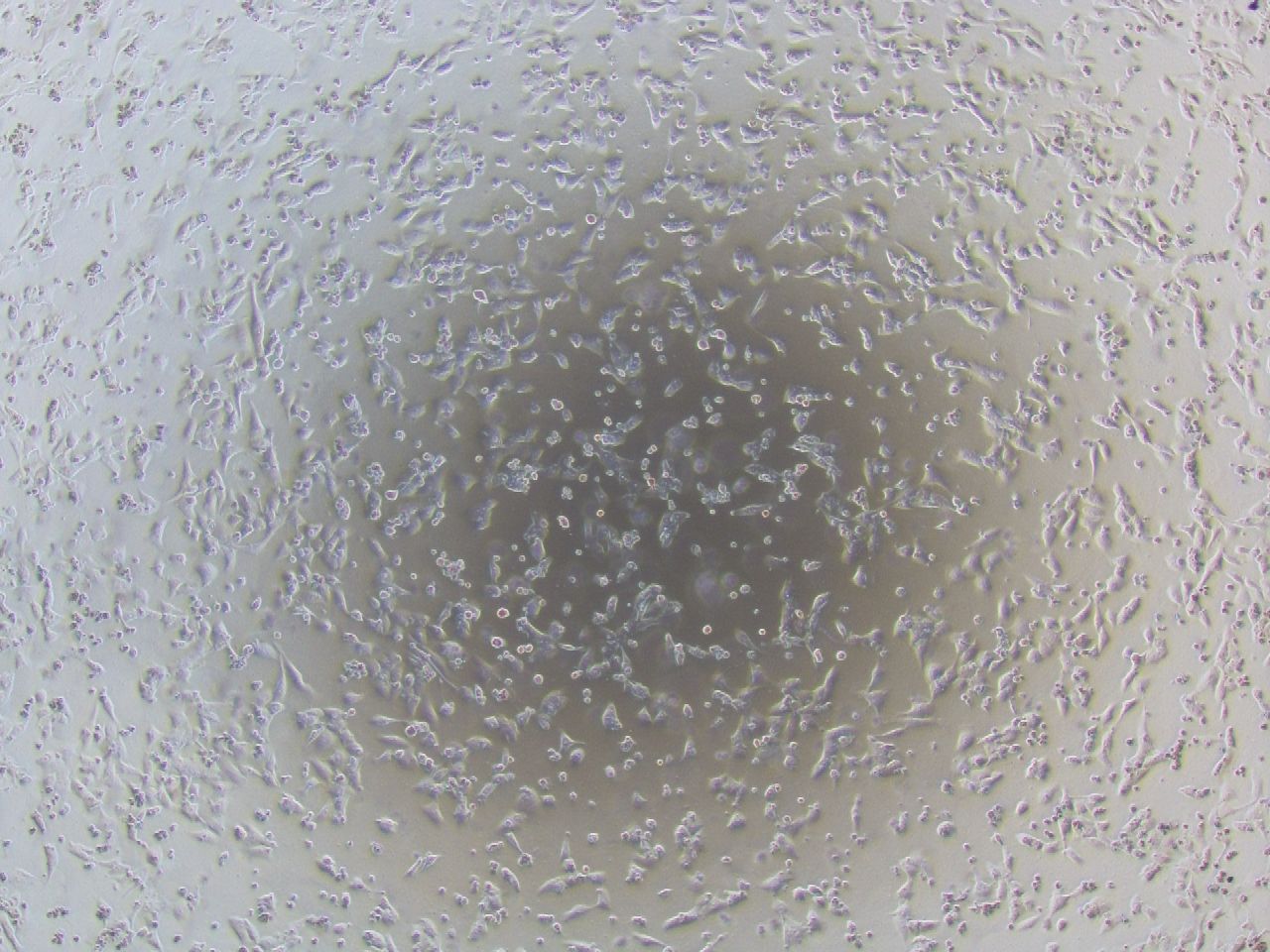

Supplement: Supplementary file 1 [file DataSheet1.ZIP › Raw data/morphology of RM-1 cells/5-4.jpg]

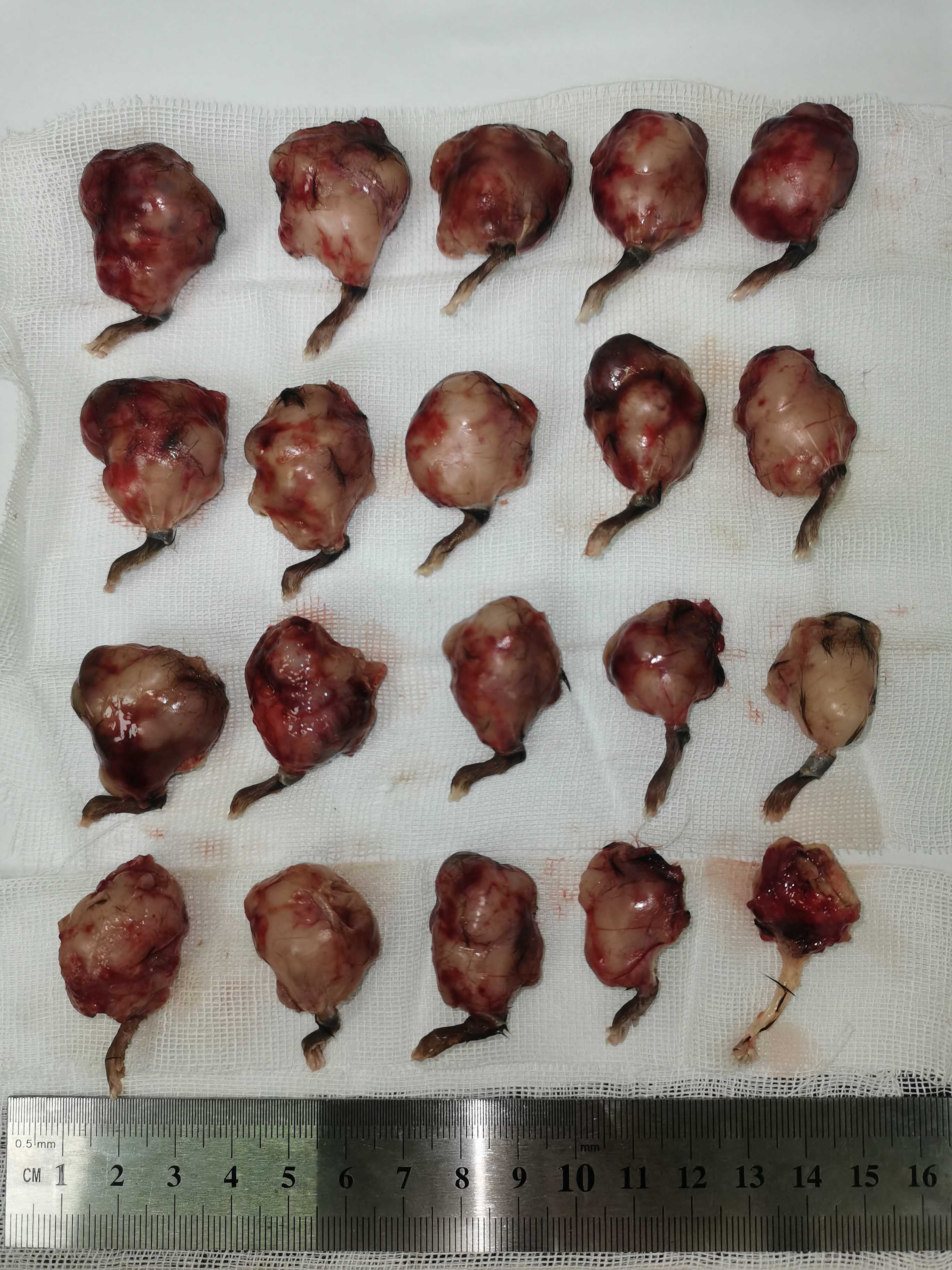

Supplement: Supplementary file 1 [file DataSheet1.ZIP › Raw data/tumor weigh & volume-SPSS&GraphPad7 statistics/tumor.jpg]

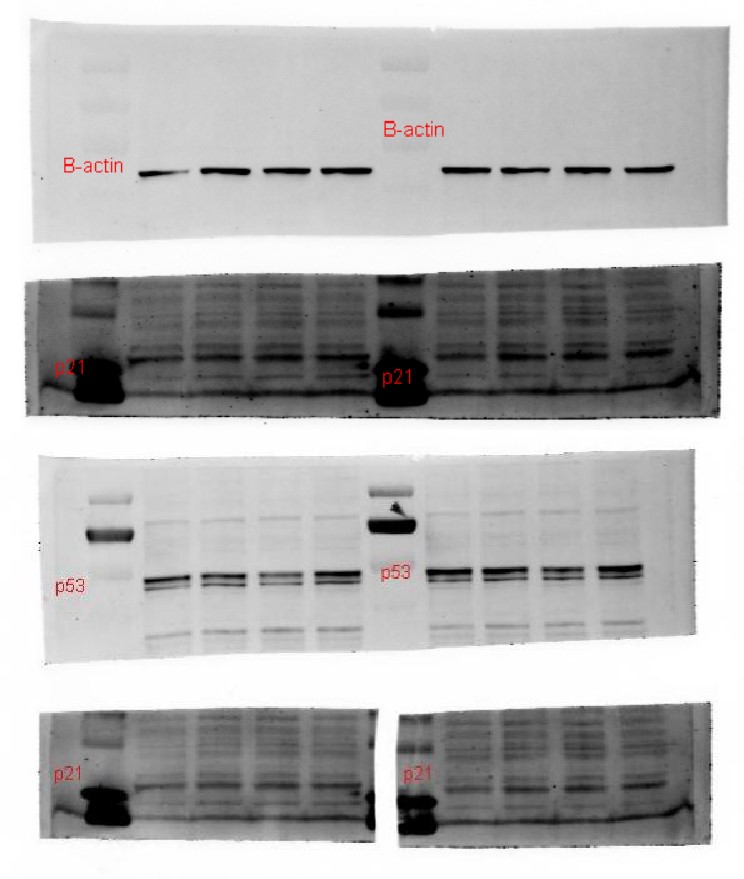

Supplement: Supplementary file 1 [file DataSheet1.ZIP › Raw data/WB/1 P53-P21.jpg]

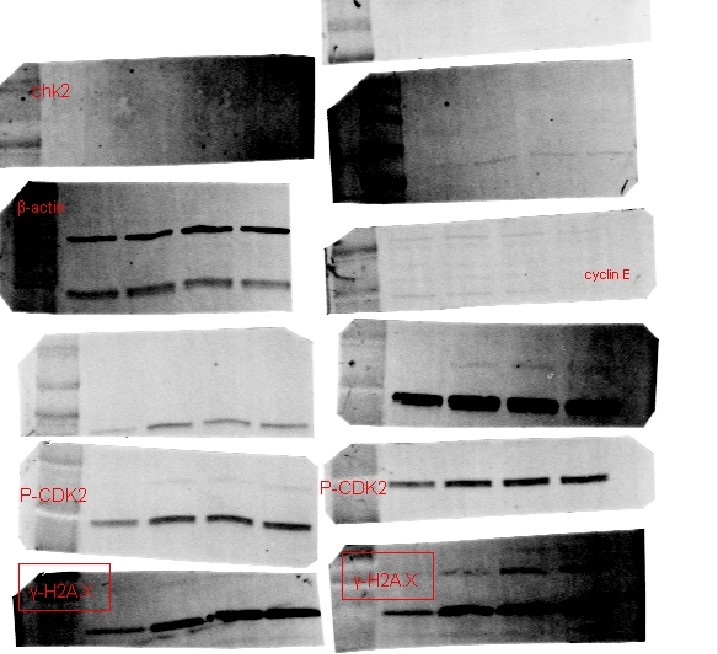

Supplement: Supplementary file 1 [file DataSheet1.ZIP › Raw data/WB/10 P-CDk2, γ-H2AX.jpg]

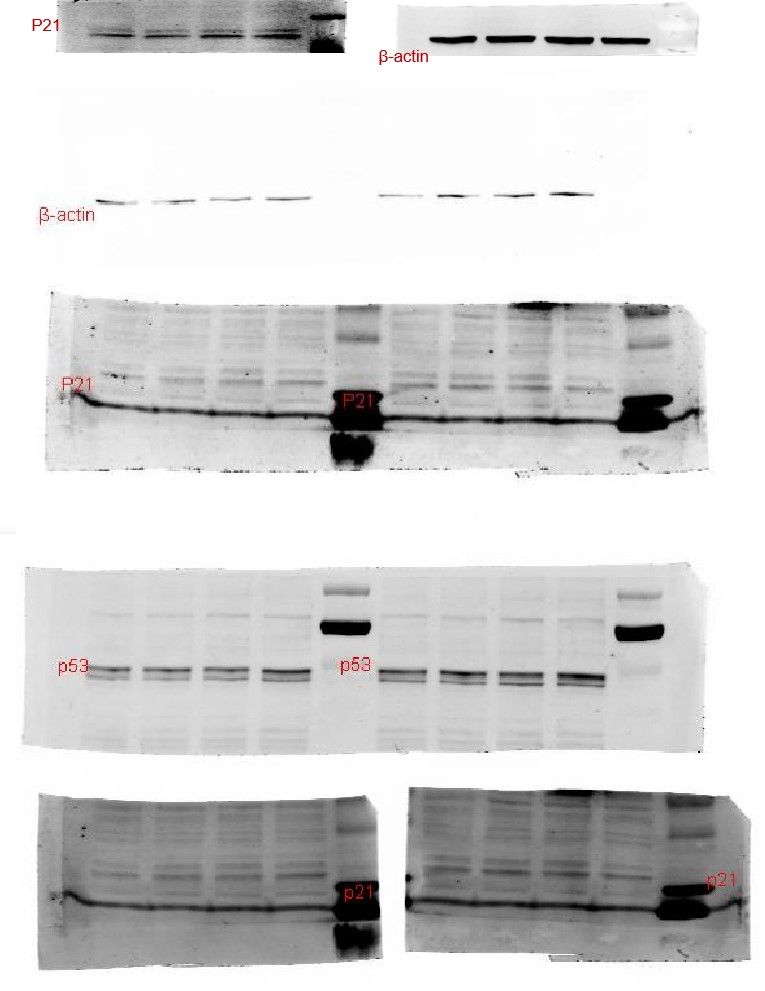

Supplement: Supplementary file 1 [file DataSheet1.ZIP › Raw data/WB/2 p21-p53.jpg]

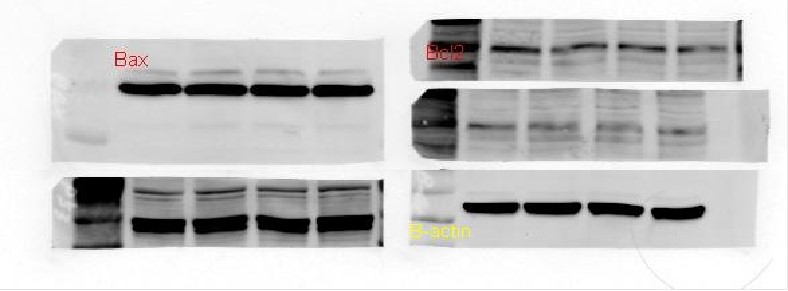

Supplement: Supplementary file 1 [file DataSheet1.ZIP › Raw data/WB/3 Bax-Bcl-2.jpg]

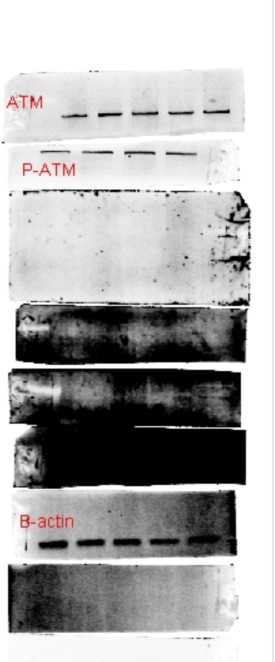

Supplement: Supplementary file 1 [file DataSheet1.ZIP › Raw data/WB/4-ATM-pATM.jpg]

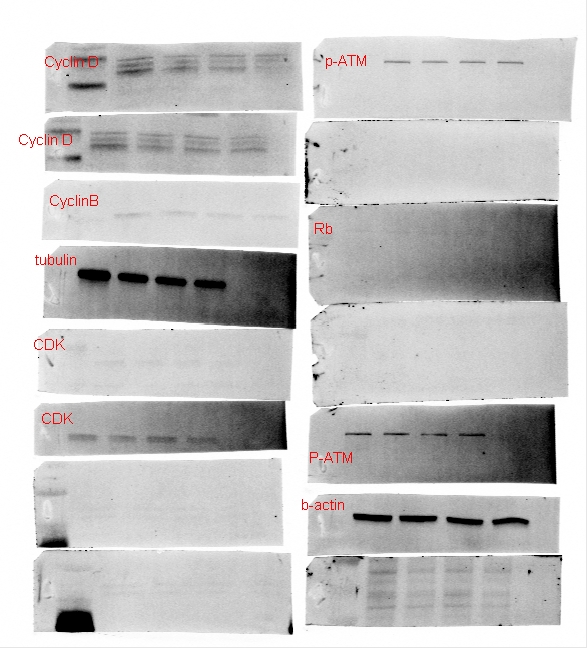

Supplement: Supplementary file 1 [file DataSheet1.ZIP › Raw data/WB/5 ATM-pATM.jpg]

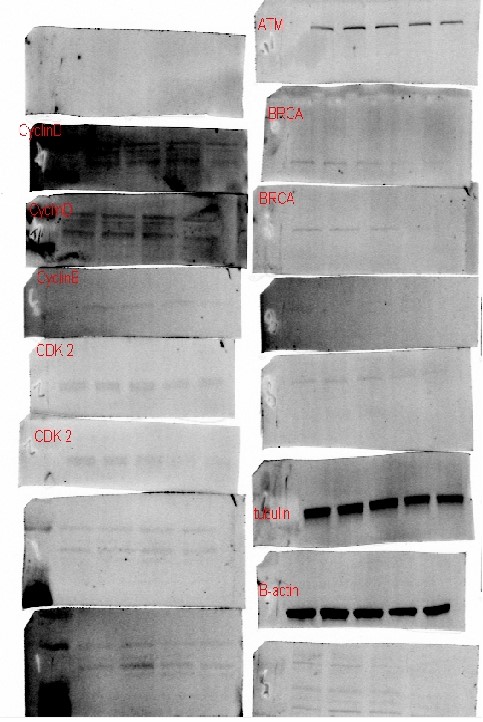

Supplement: Supplementary file 1 [file DataSheet1.ZIP › Raw data/WB/6 BRCA,CDK.jpg]

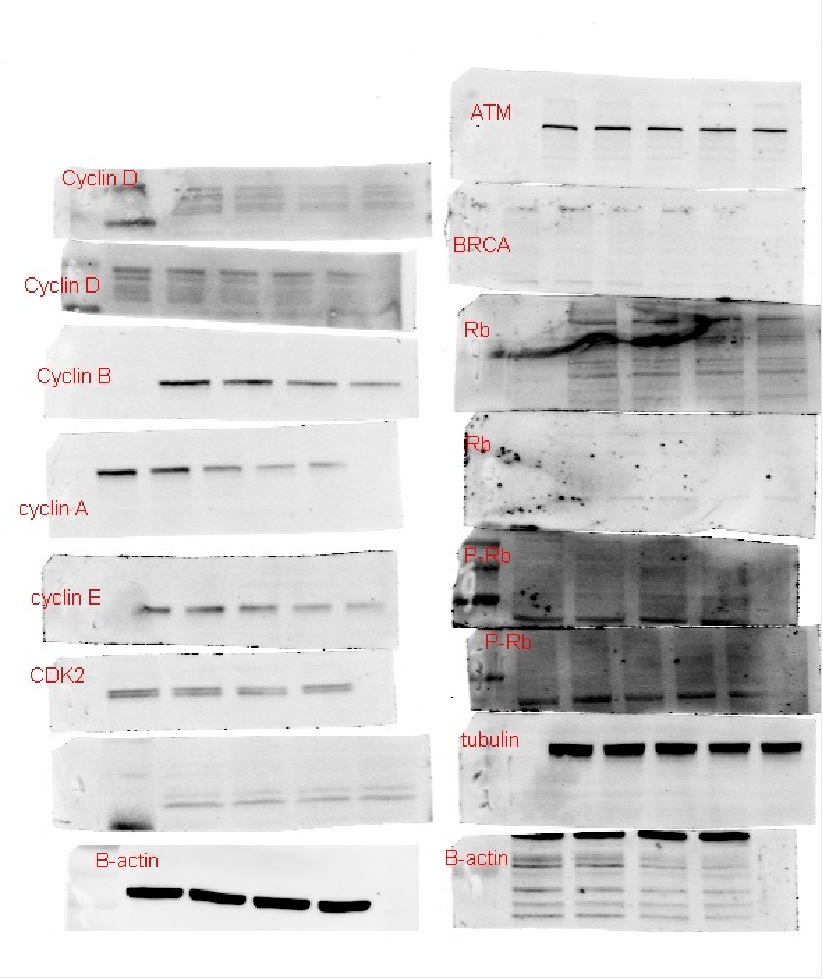

Supplement: Supplementary file 1 [file DataSheet1.ZIP › Raw data/WB/7 CDK2,A,B,D,E,ATM.png]

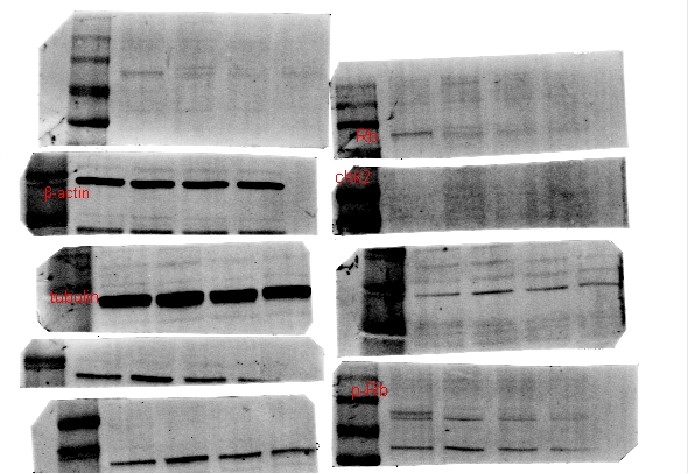

Supplement: Supplementary file 1 [file DataSheet1.ZIP › Raw data/WB/8 Rb p-Rb.jpg]

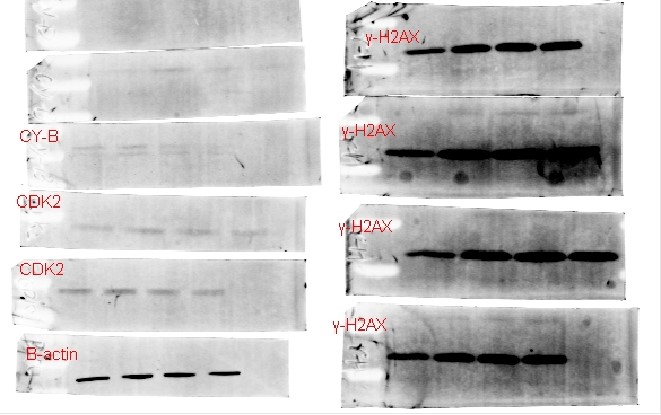

Supplement: Supplementary file 1 [file DataSheet1.ZIP › Raw data/WB/9 γ-H2A.X.jpg]

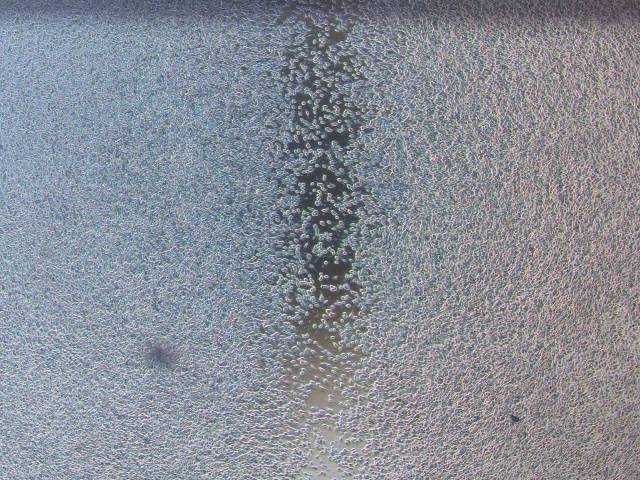

Supplement: Supplementary file 1 [file DataSheet1.ZIP › Raw data/wound healing piture & data-SPSS&GraphPad7 statistics/0-1.2.jpg]

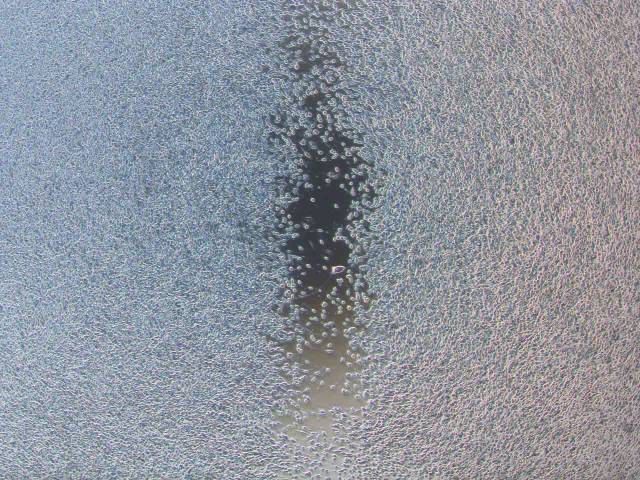

Supplement: Supplementary file 1 [file DataSheet1.ZIP › Raw data/wound healing piture & data-SPSS&GraphPad7 statistics/0-1.3.jpg]

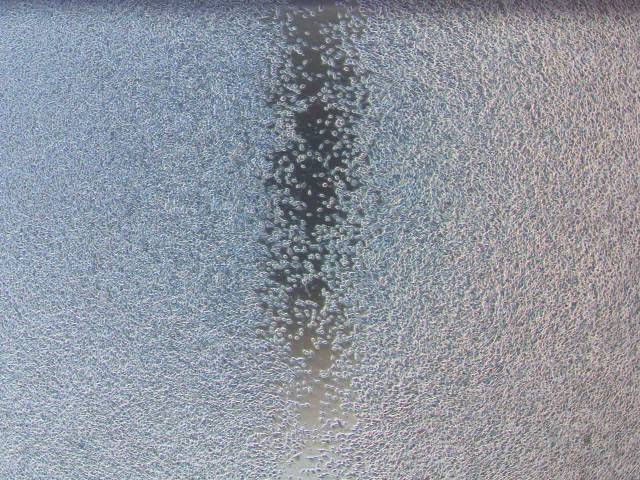

Supplement: Supplementary file 1 [file DataSheet1.ZIP › Raw data/wound healing piture & data-SPSS&GraphPad7 statistics/0-1.4.jpg]

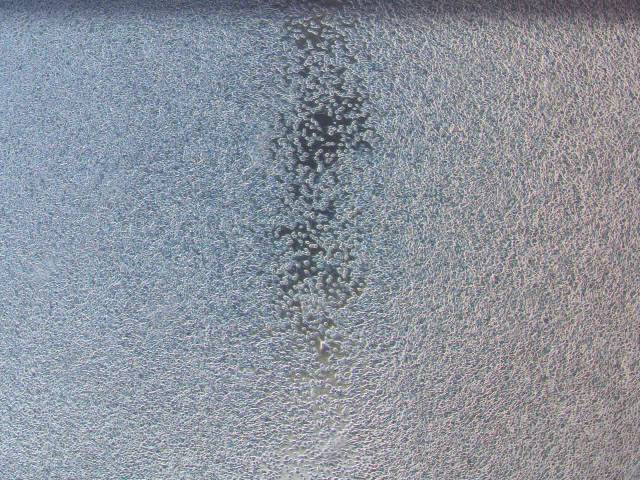

Supplement: Supplementary file 1 [file DataSheet1.ZIP › Raw data/wound healing piture & data-SPSS&GraphPad7 statistics/0-2.2.jpg]

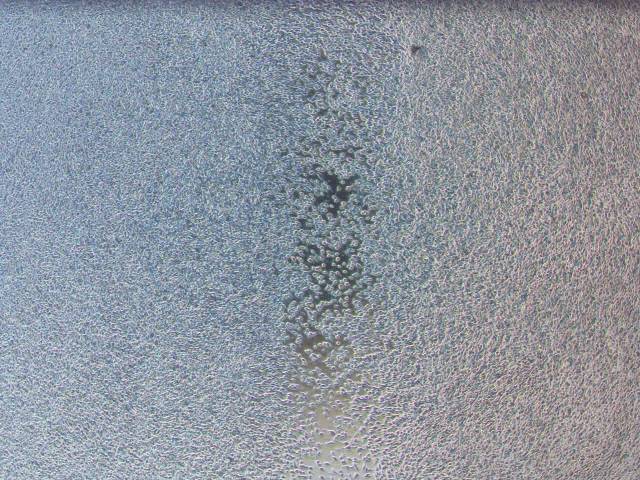

Supplement: Supplementary file 1 [file DataSheet1.ZIP › Raw data/wound healing piture & data-SPSS&GraphPad7 statistics/0-2.4.jpg]

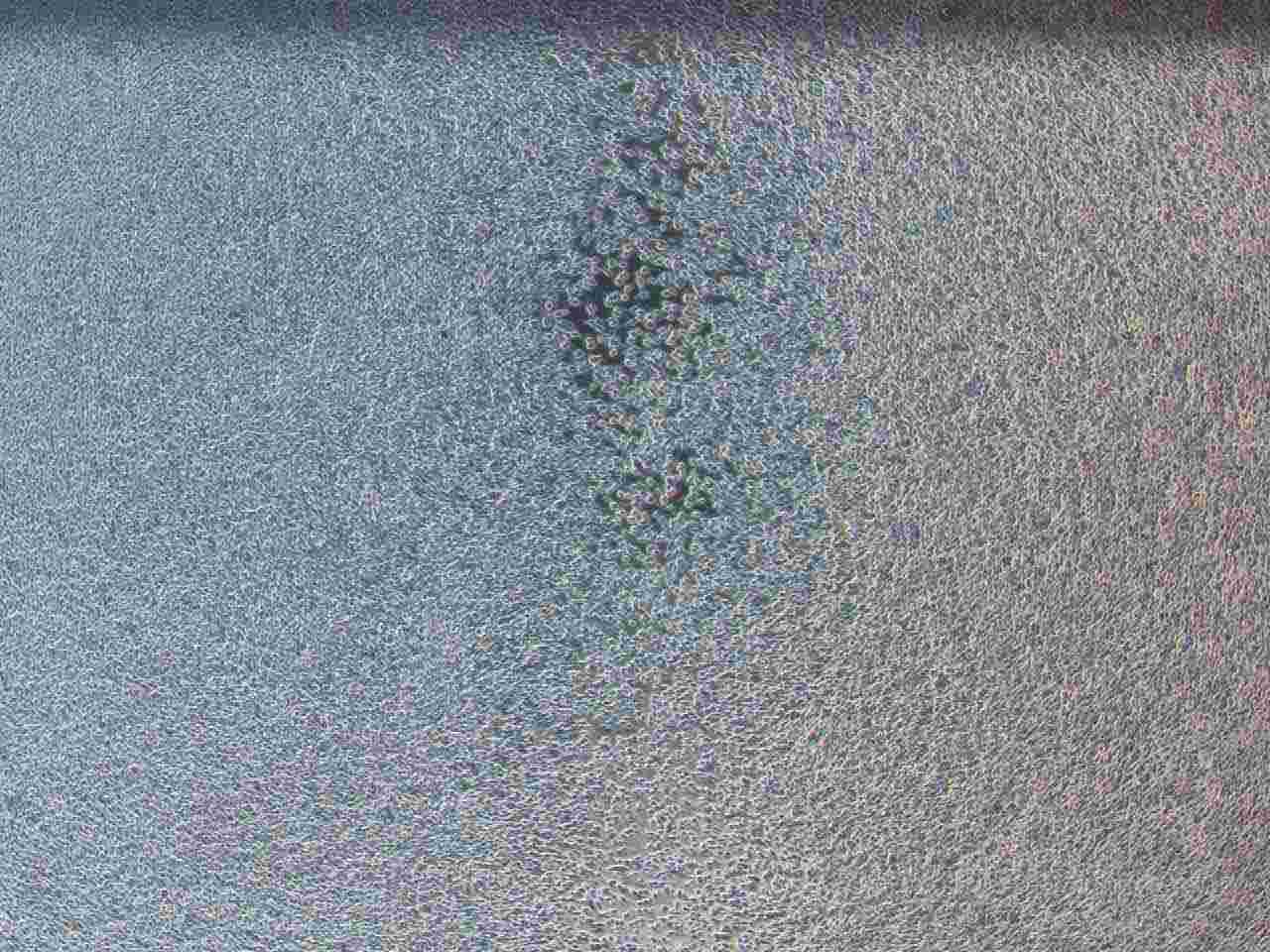

Supplement: Supplementary file 1 [file DataSheet1.ZIP › Raw data/wound healing piture & data-SPSS&GraphPad7 statistics/0-3.4.jpg]

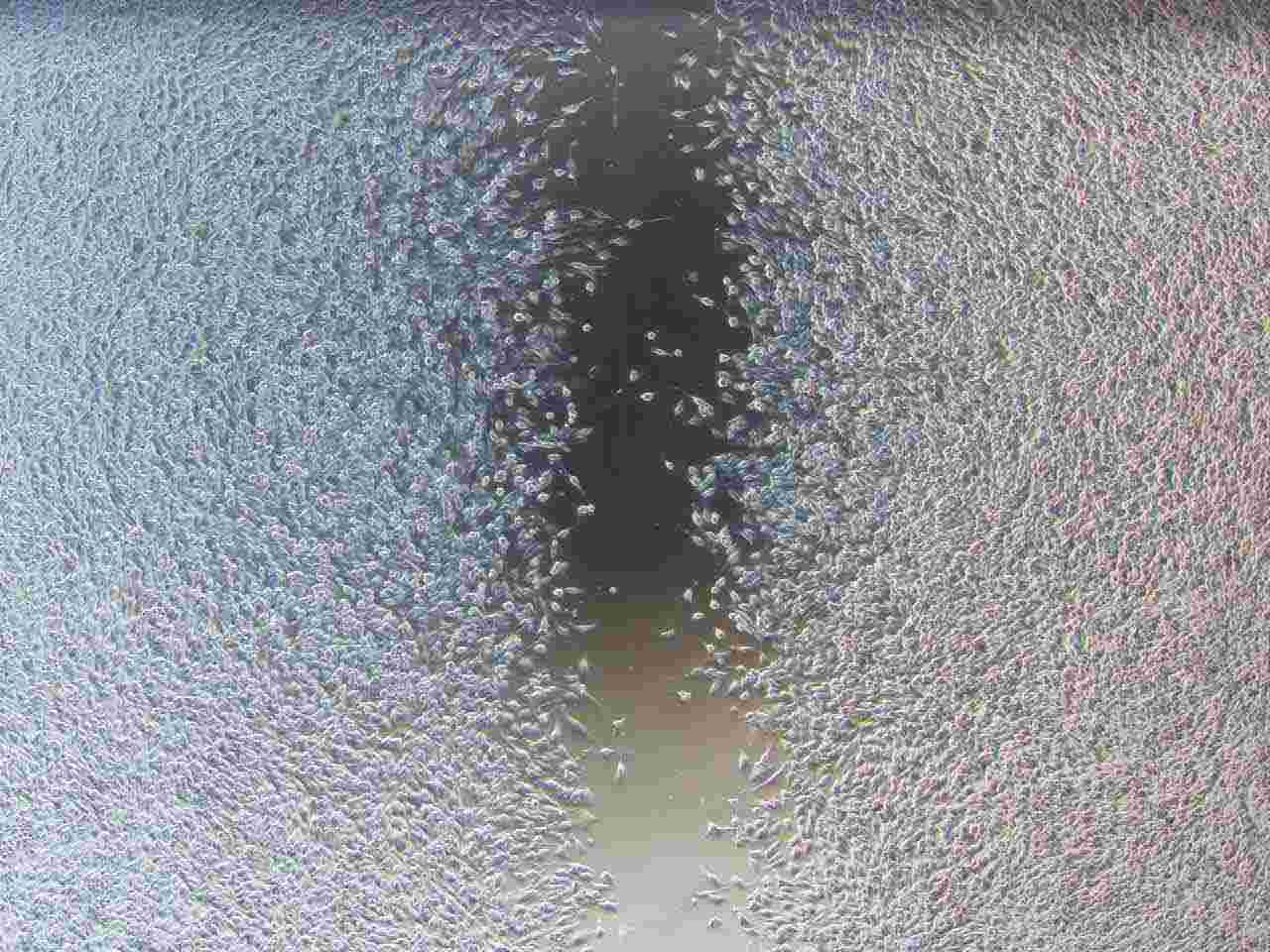

Supplement: Supplementary file 1 [file DataSheet1.ZIP › Raw data/wound healing piture & data-SPSS&GraphPad7 statistics/0.625-1.4.jpg]

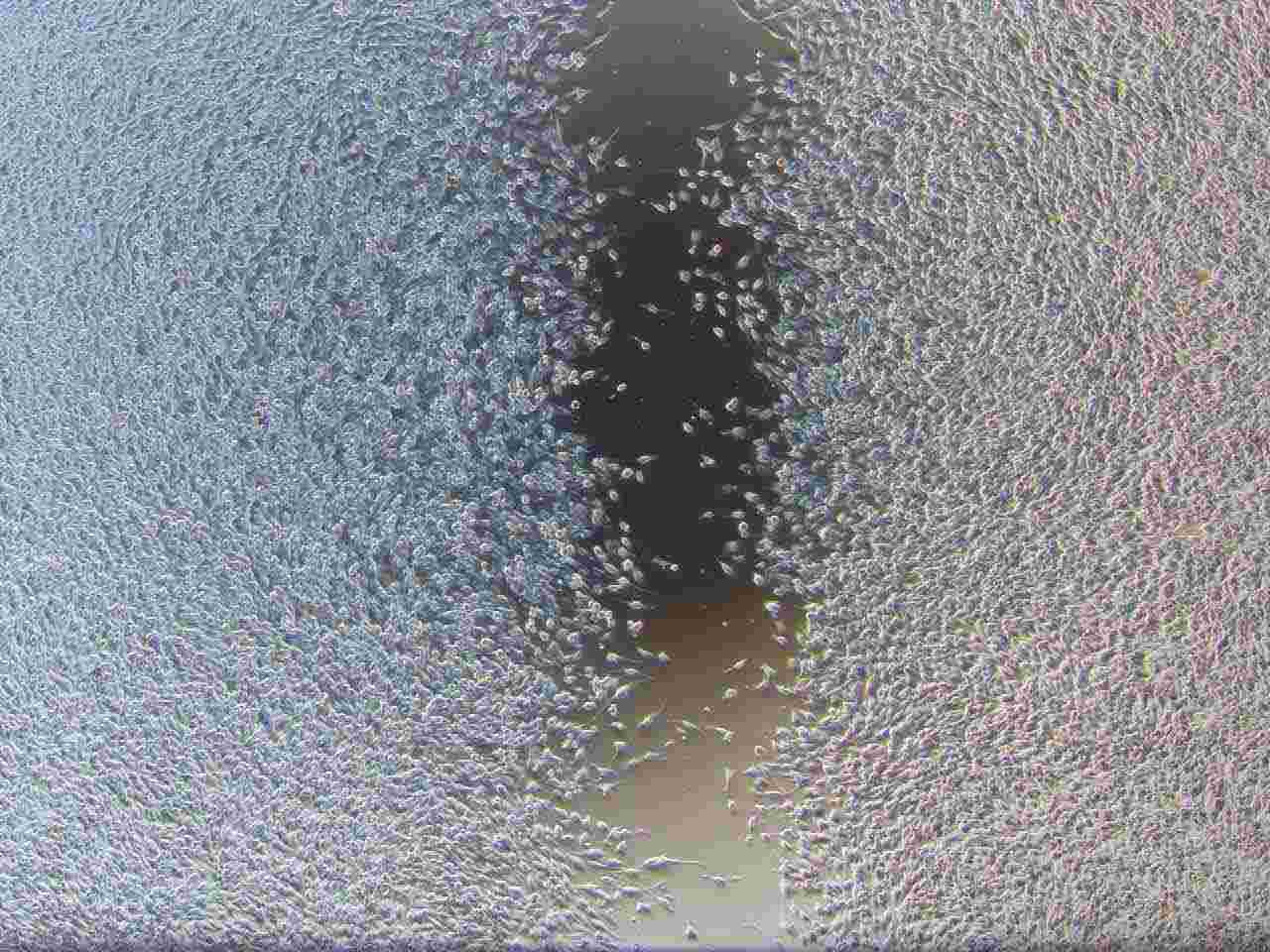

Supplement: Supplementary file 1 [file DataSheet1.ZIP › Raw data/wound healing piture & data-SPSS&GraphPad7 statistics/0.625-1.5.jpg]

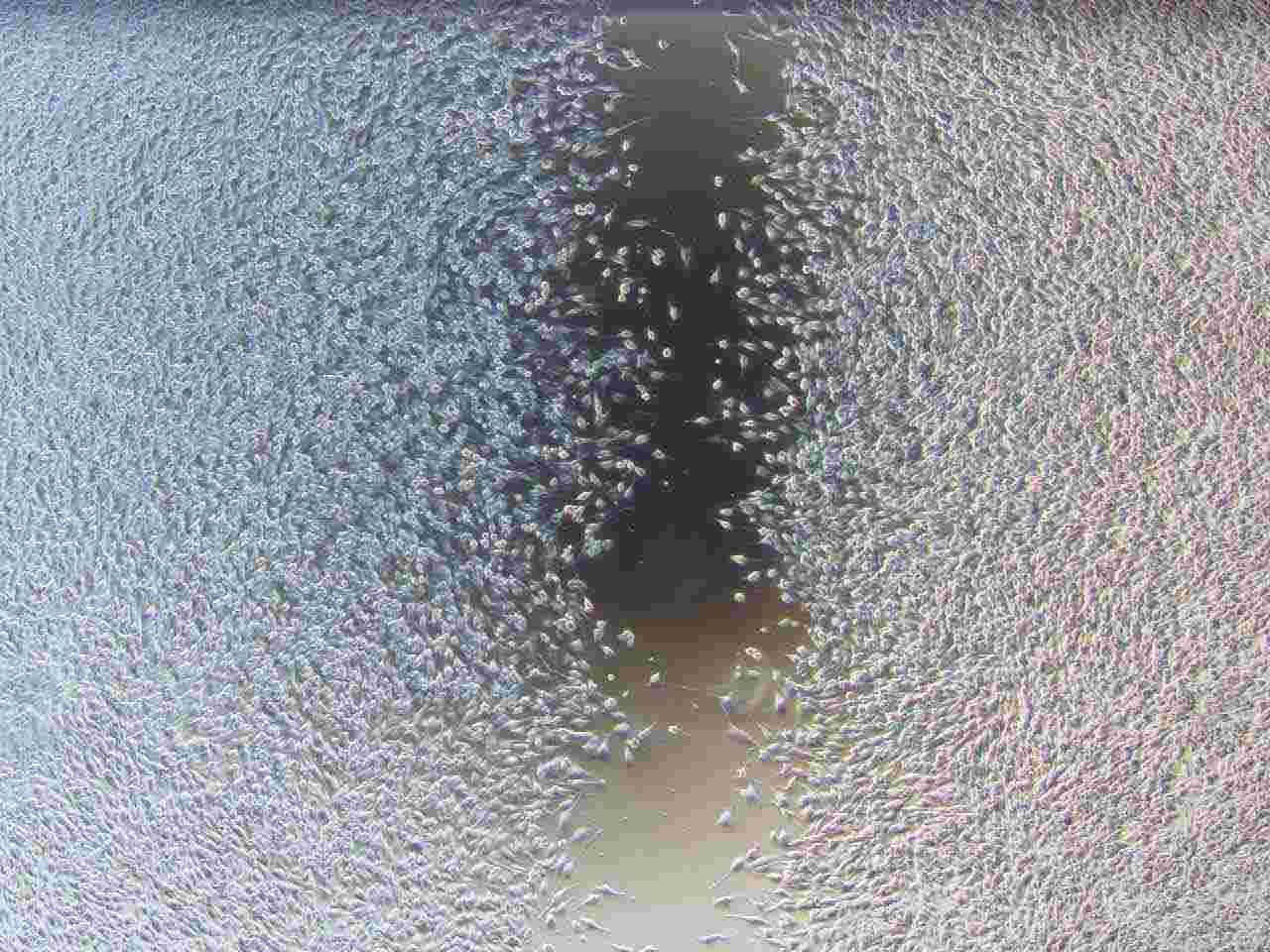

Supplement: Supplementary file 1 [file DataSheet1.ZIP › Raw data/wound healing piture & data-SPSS&GraphPad7 statistics/0.625-1.6.jpg]

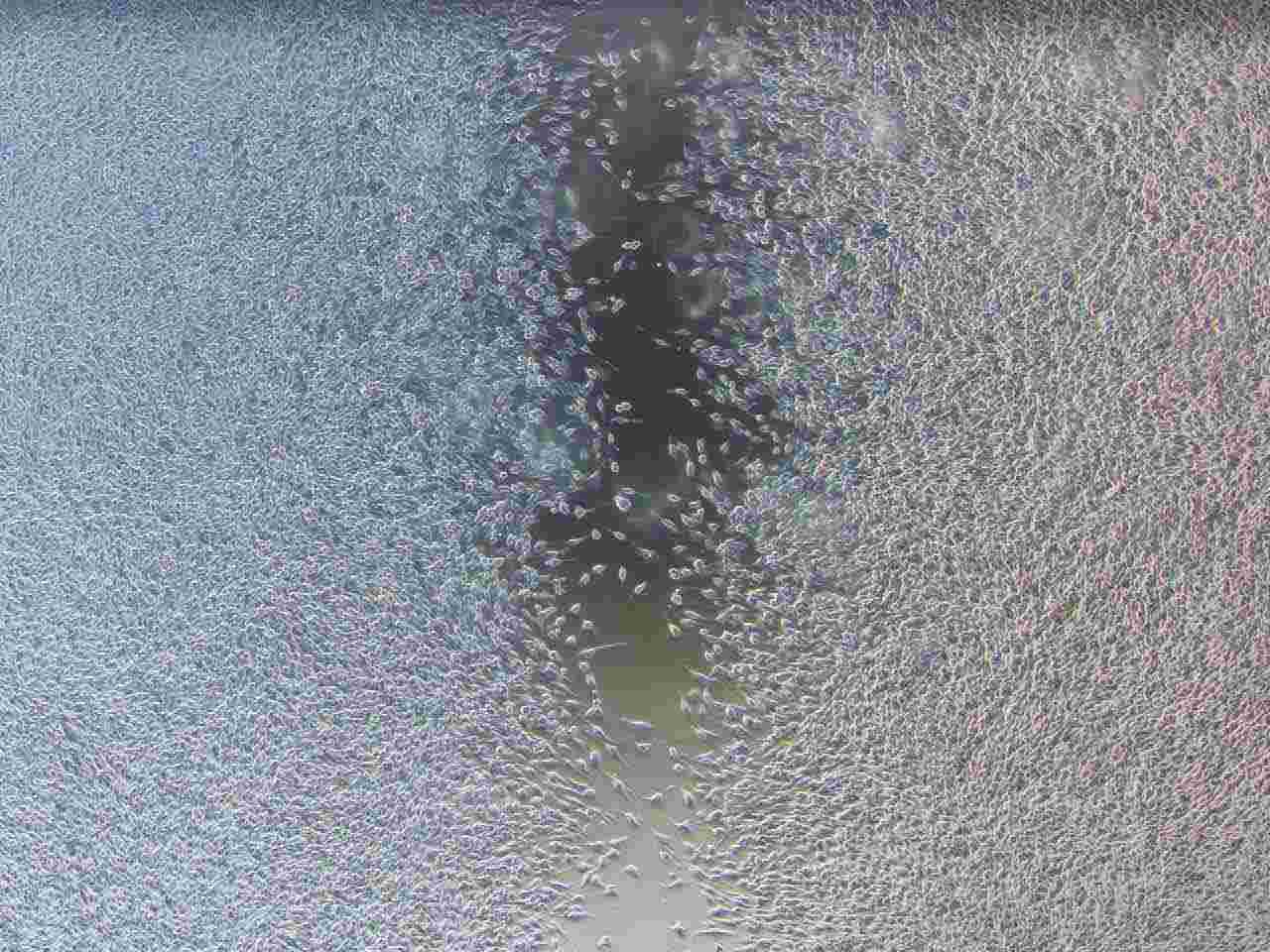

Supplement: Supplementary file 1 [file DataSheet1.ZIP › Raw data/wound healing piture & data-SPSS&GraphPad7 statistics/0.625-2.2.jpg]

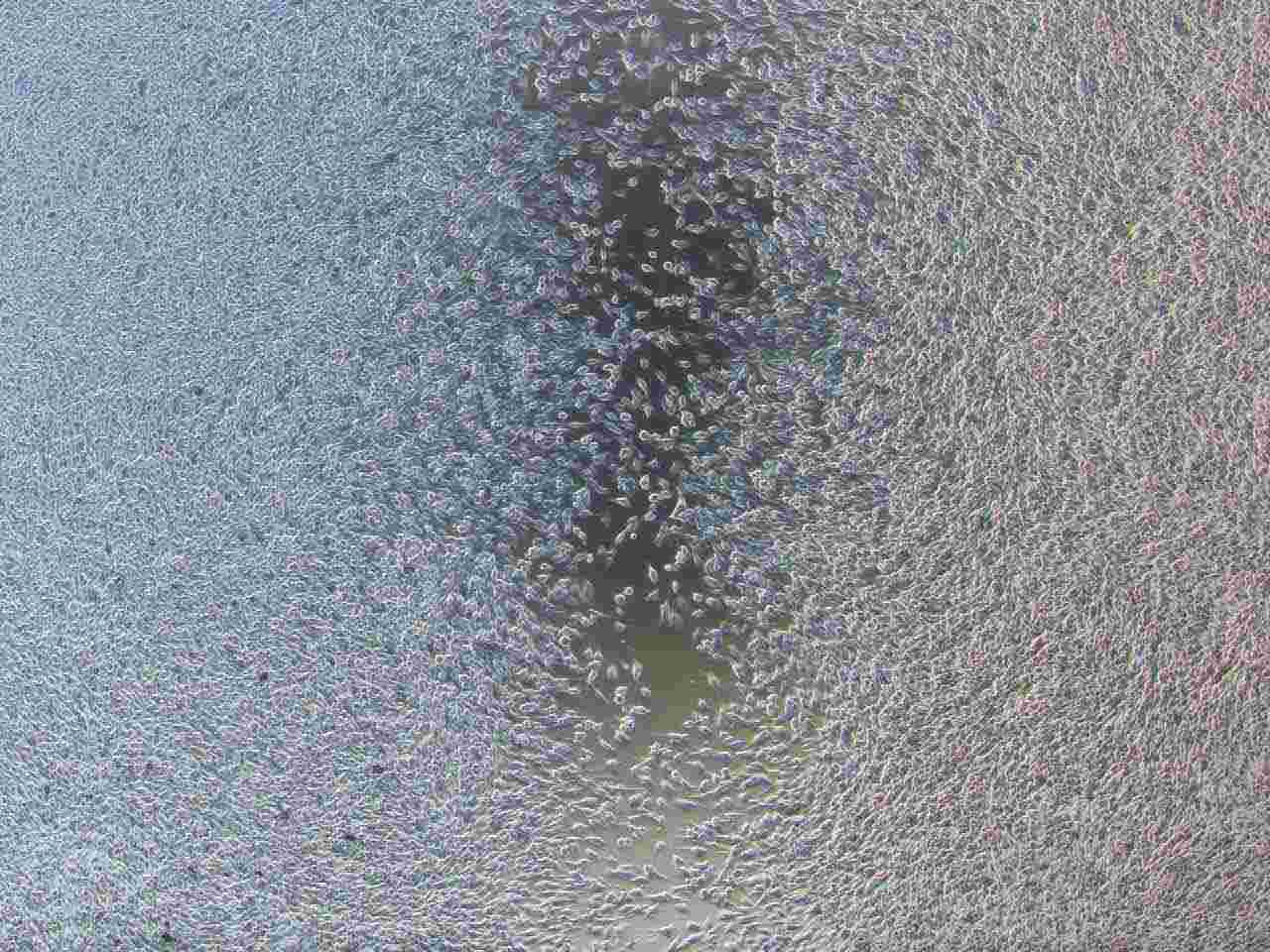

Supplement: Supplementary file 1 [file DataSheet1.ZIP › Raw data/wound healing piture & data-SPSS&GraphPad7 statistics/0.625-2.3.jpg]

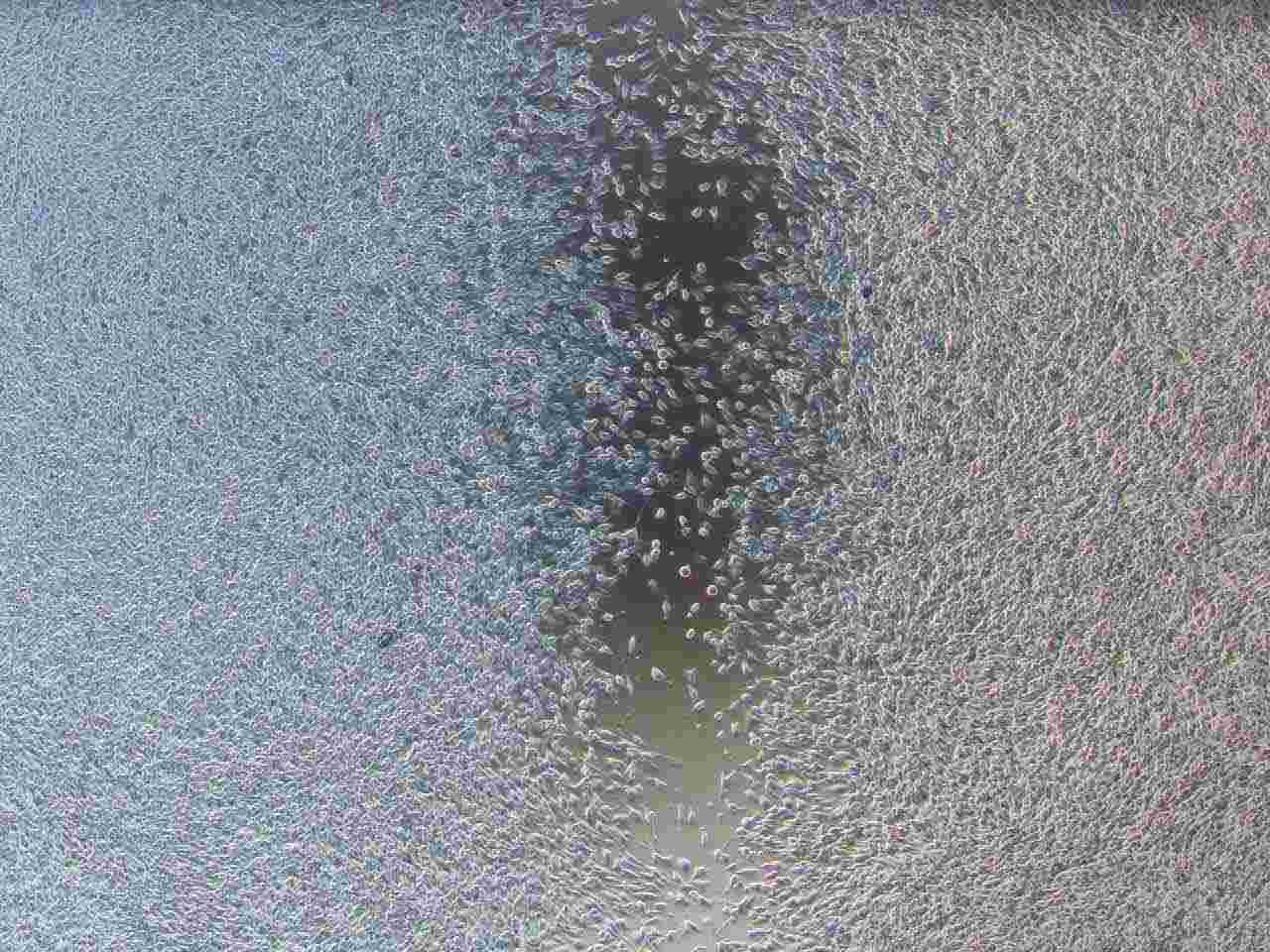

Supplement: Supplementary file 1 [file DataSheet1.ZIP › Raw data/wound healing piture & data-SPSS&GraphPad7 statistics/0.625-2.4.jpg]

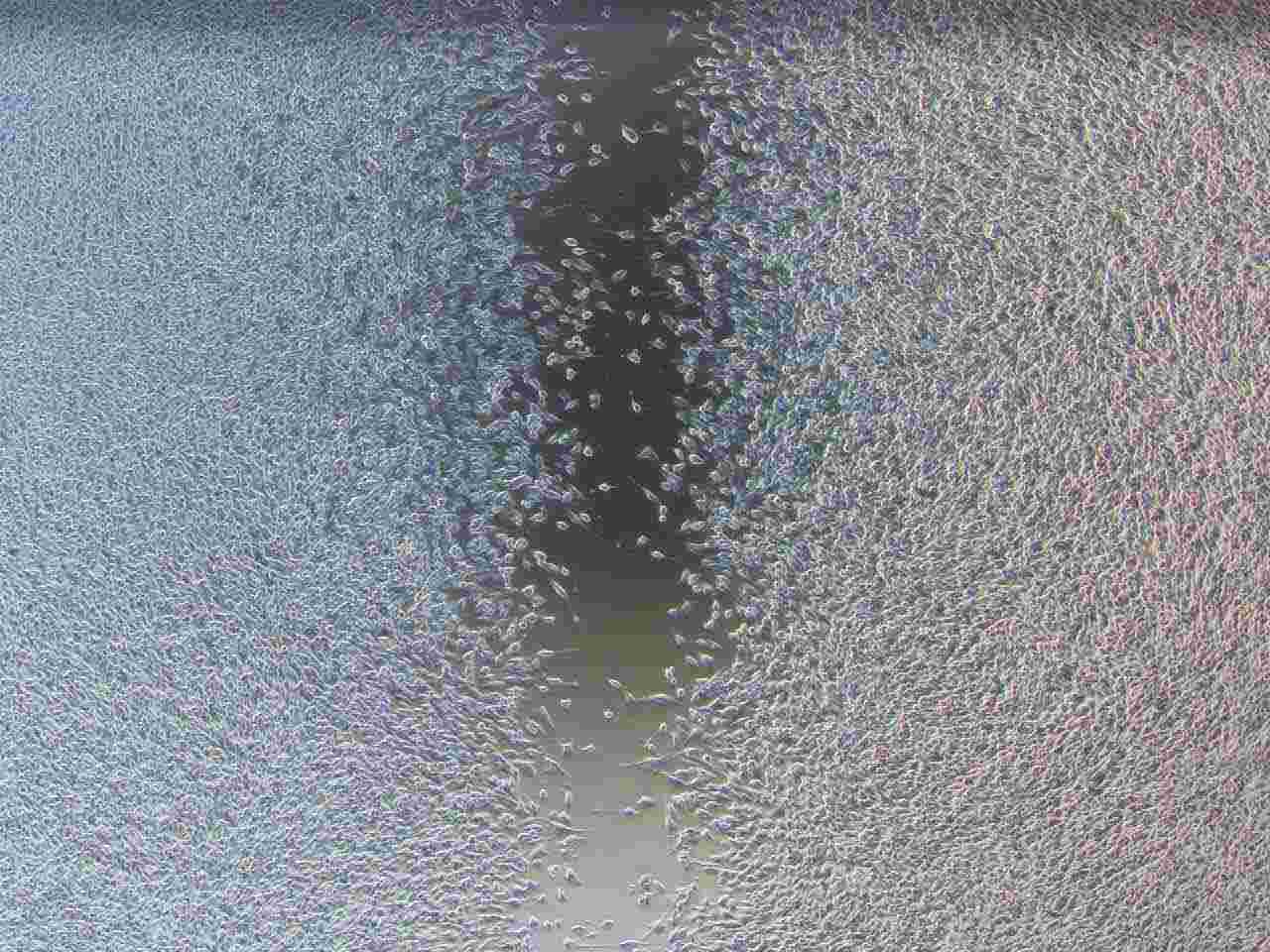

Supplement: Supplementary file 1 [file DataSheet1.ZIP › Raw data/wound healing piture & data-SPSS&GraphPad7 statistics/0.625-3.2.jpg]

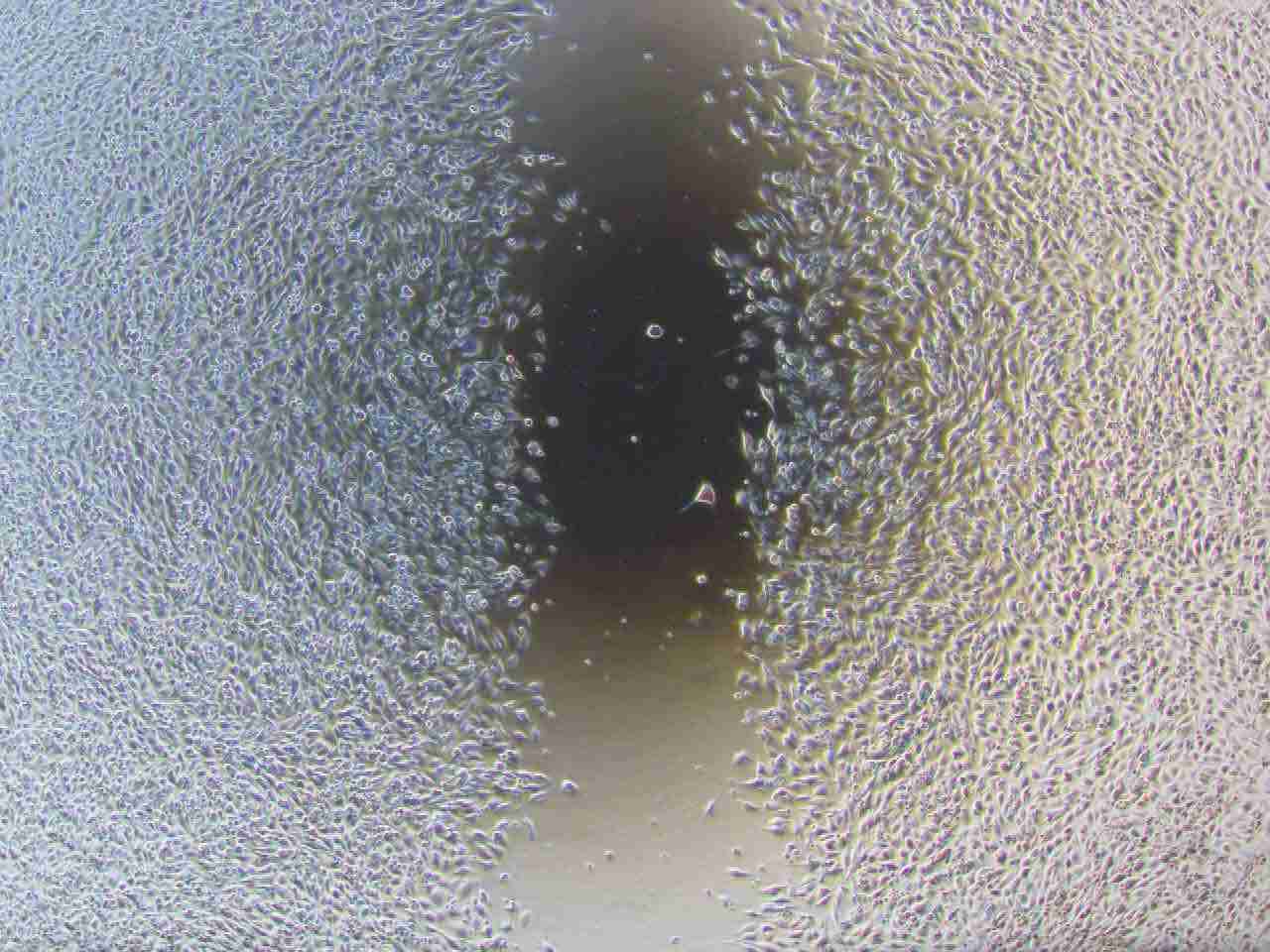

Supplement: Supplementary file 1 [file DataSheet1.ZIP › Raw data/wound healing piture & data-SPSS&GraphPad7 statistics/1.25-3.1.jpg]

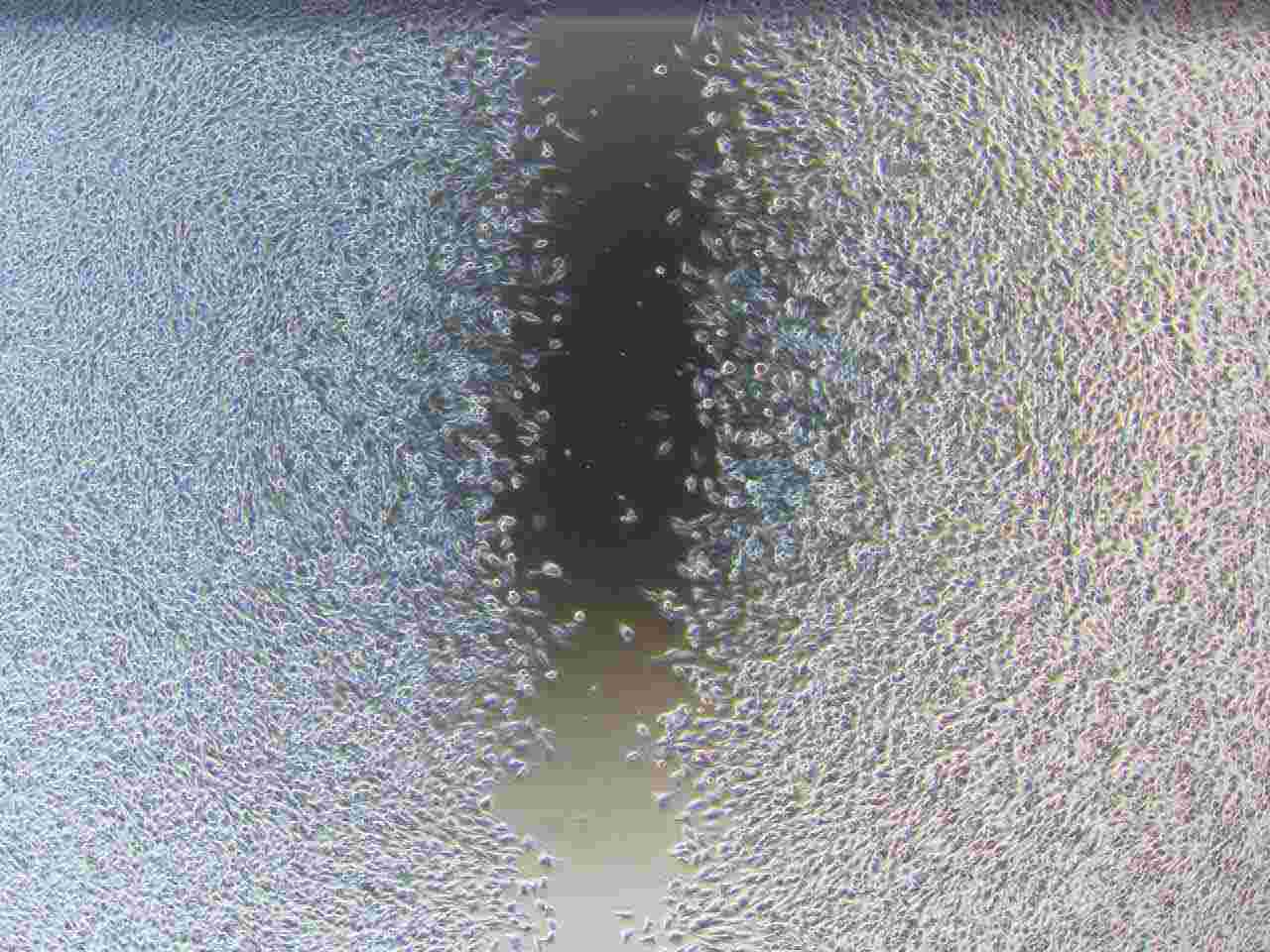

Supplement: Supplementary file 1 [file DataSheet1.ZIP › Raw data/wound healing piture & data-SPSS&GraphPad7 statistics/1.25-3.2.jpg]

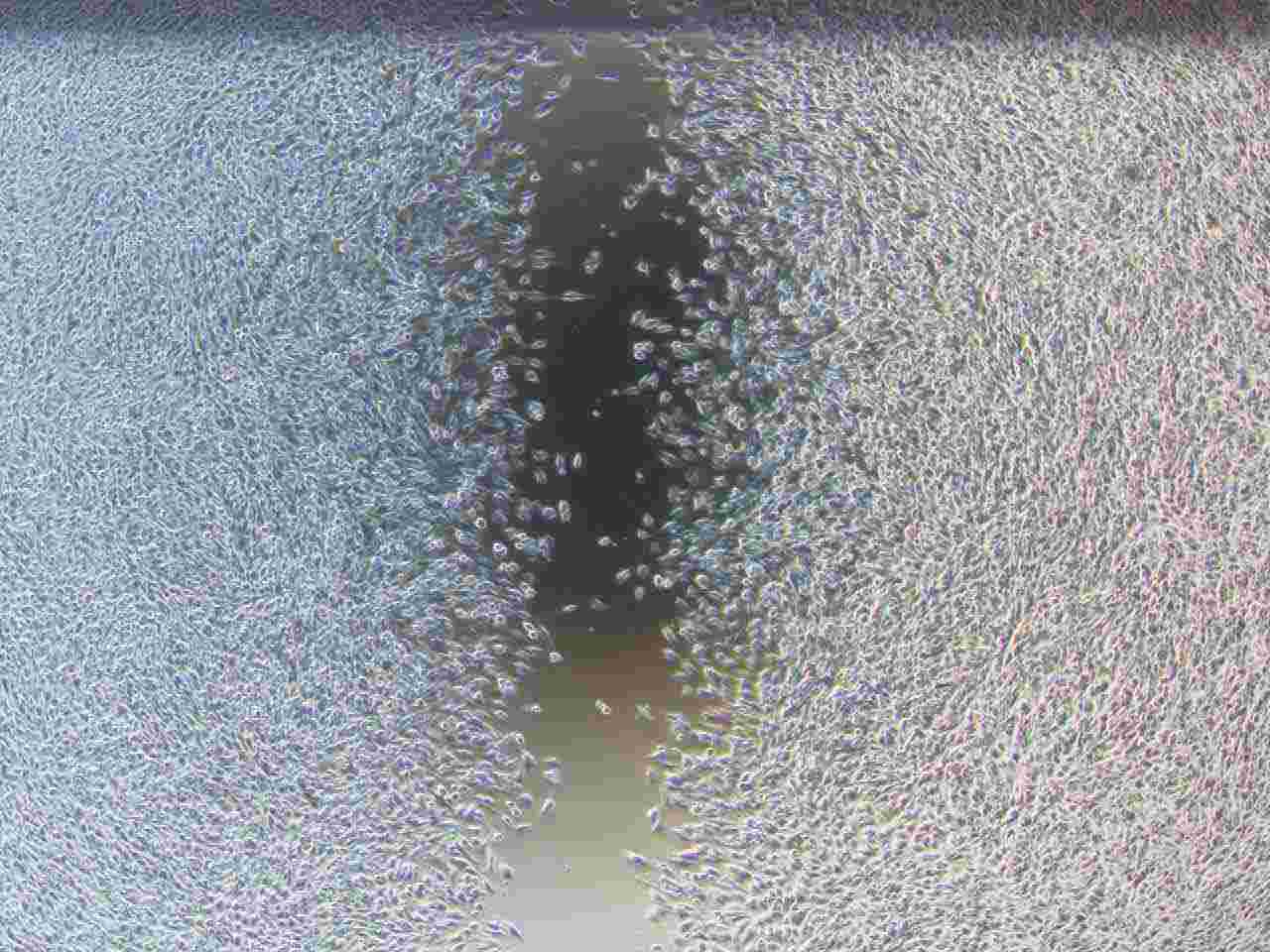

Supplement: Supplementary file 1 [file DataSheet1.ZIP › Raw data/wound healing piture & data-SPSS&GraphPad7 statistics/1.25-3.4.jpg]

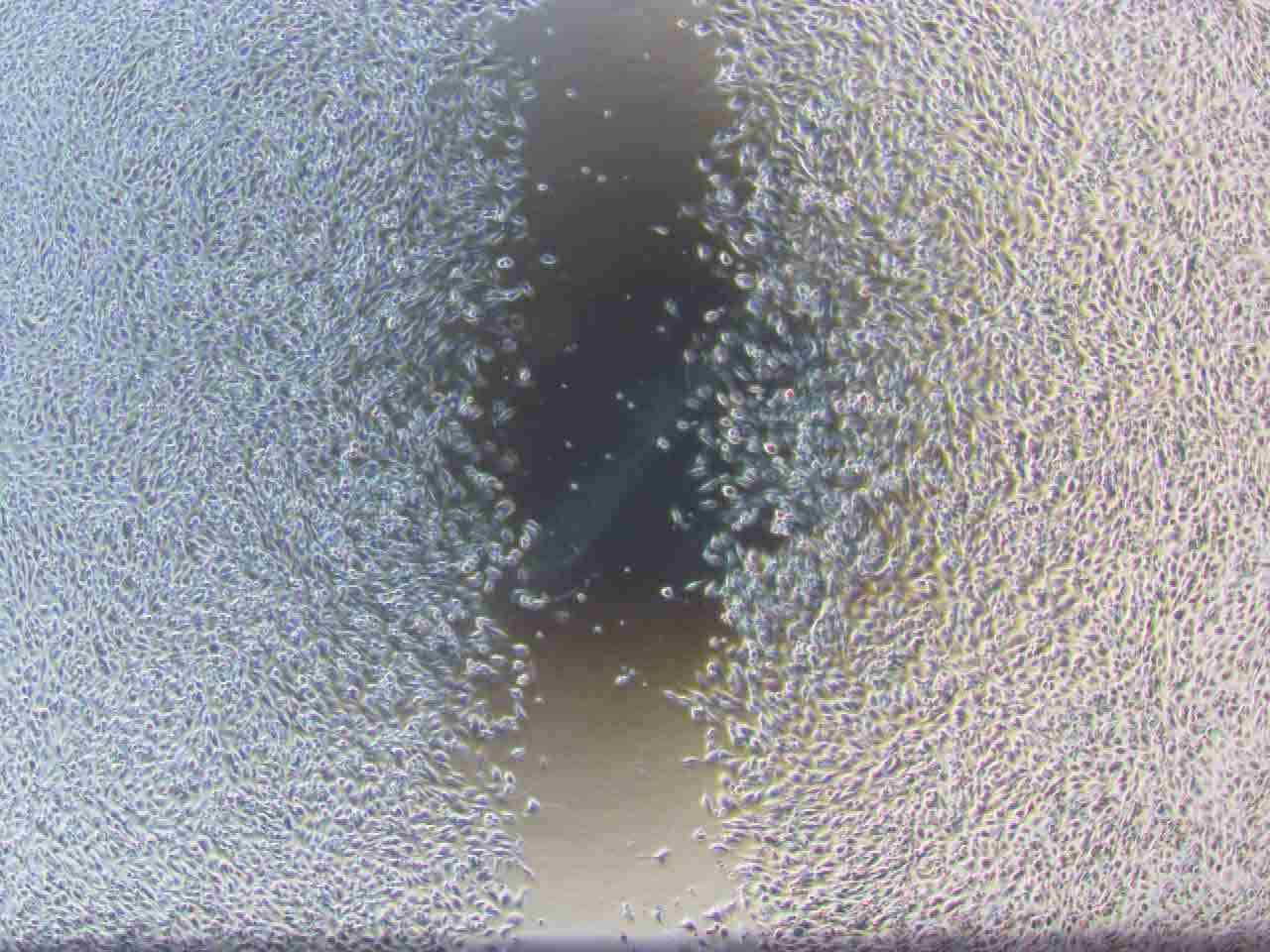

Supplement: Supplementary file 1 [file DataSheet1.ZIP › Raw data/wound healing piture & data-SPSS&GraphPad7 statistics/1.25-3.5.jpg]

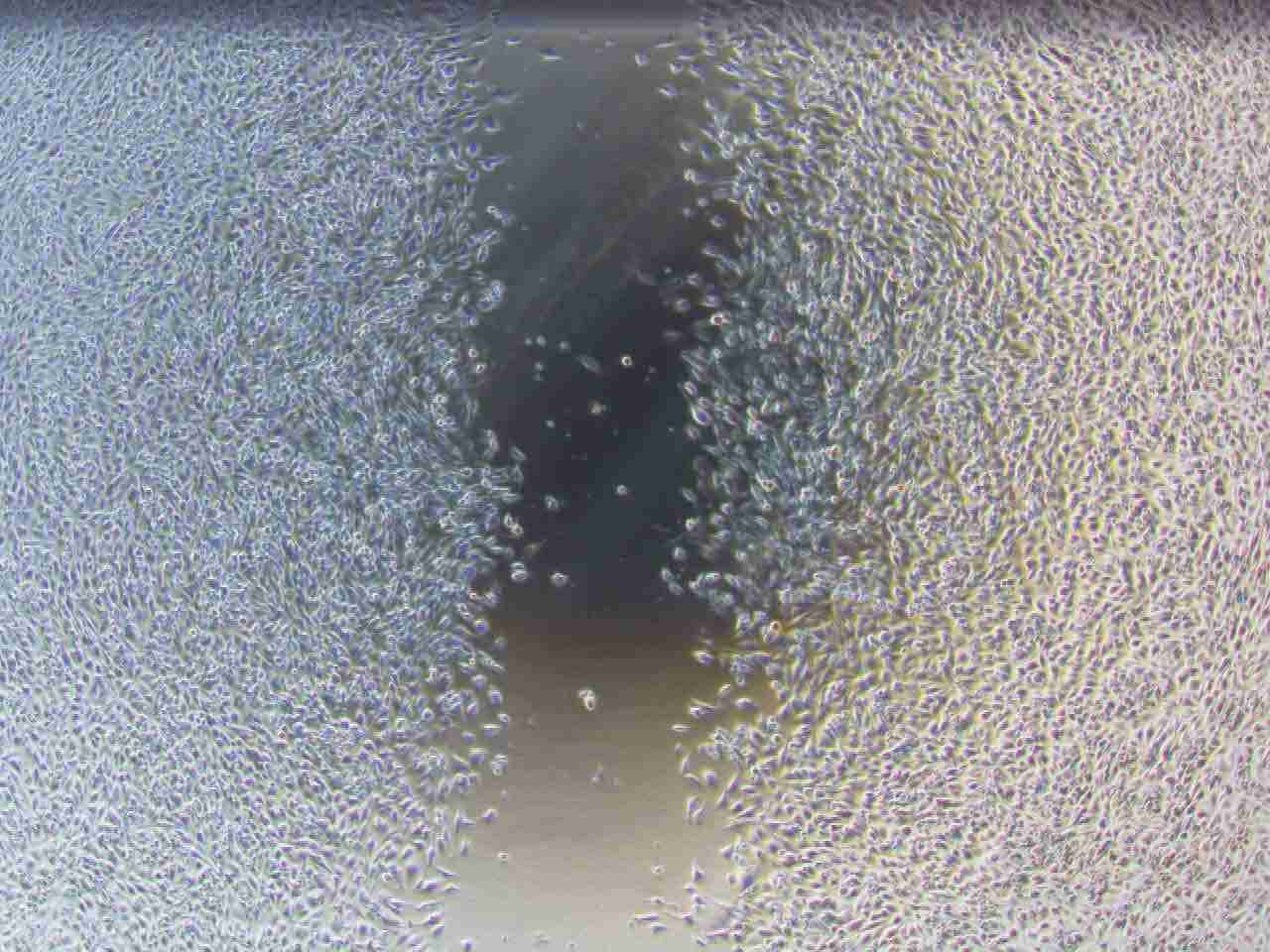

Supplement: Supplementary file 1 [file DataSheet1.ZIP › Raw data/wound healing piture & data-SPSS&GraphPad7 statistics/1.25-3.6.jpg]

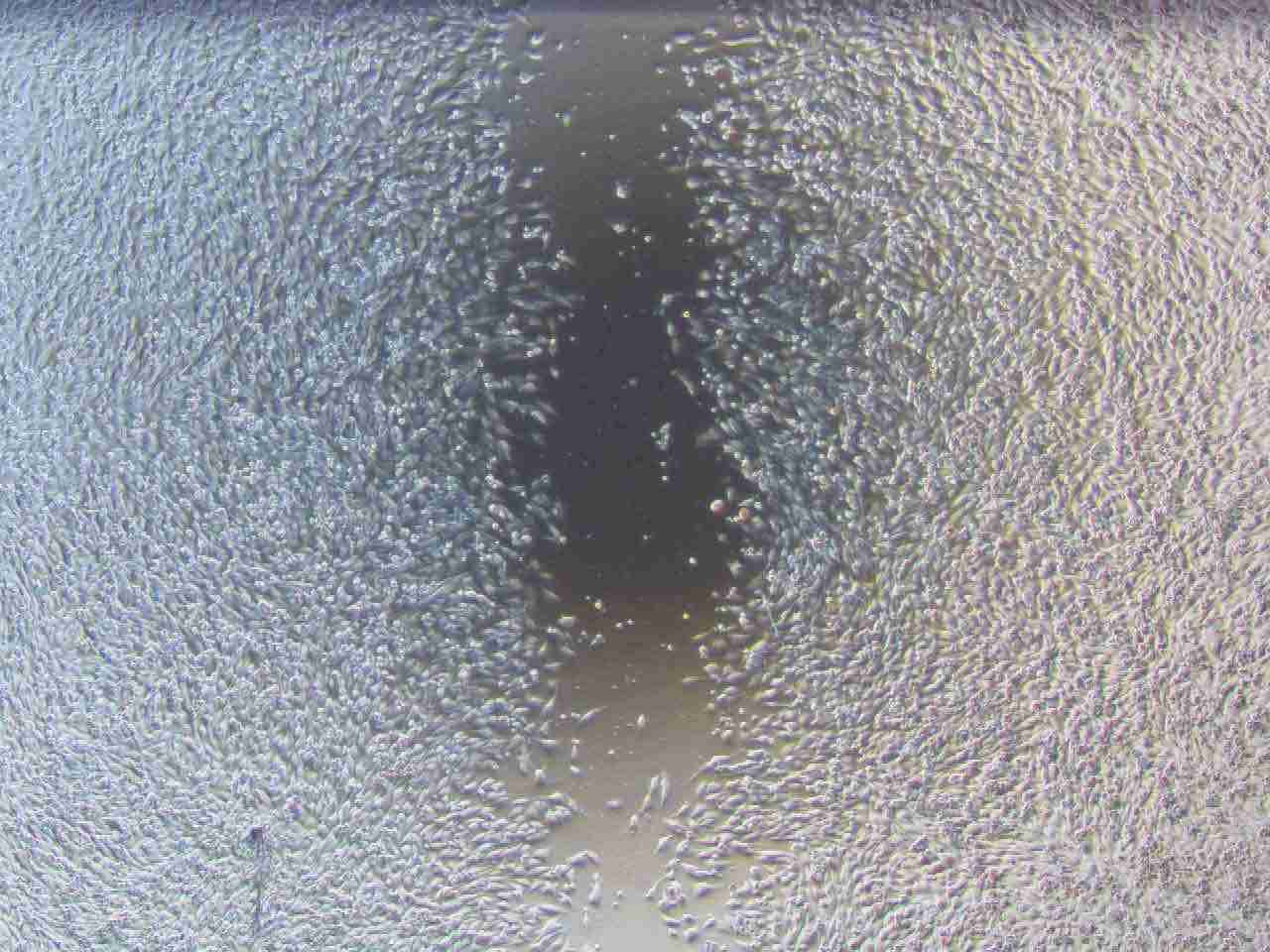

Supplement: Supplementary file 1 [file DataSheet1.ZIP › Raw data/wound healing piture & data-SPSS&GraphPad7 statistics/2.5-2.2.jpg]

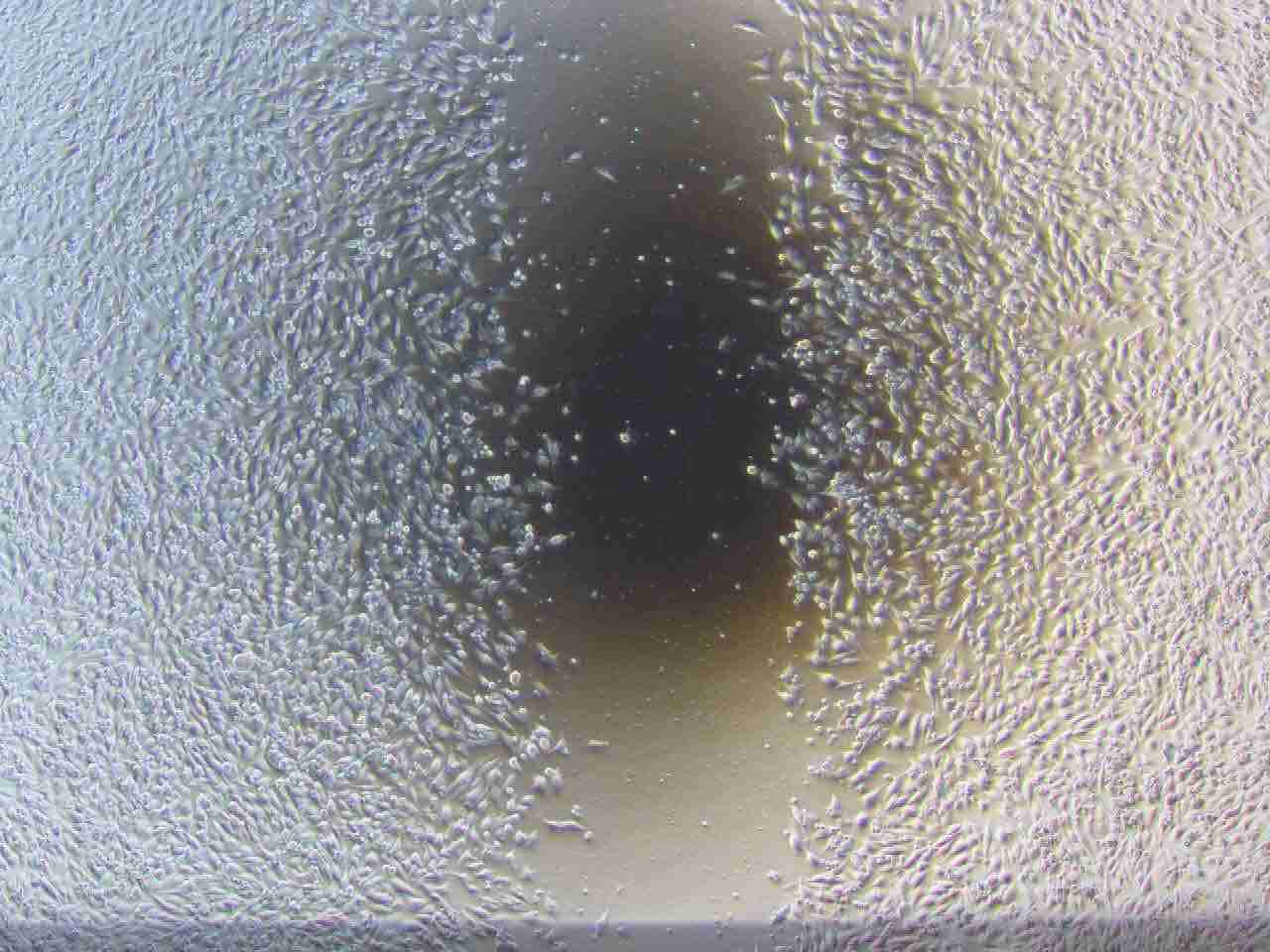

Supplement: Supplementary file 1 [file DataSheet1.ZIP › Raw data/wound healing piture & data-SPSS&GraphPad7 statistics/2.5-3.1.jpg]

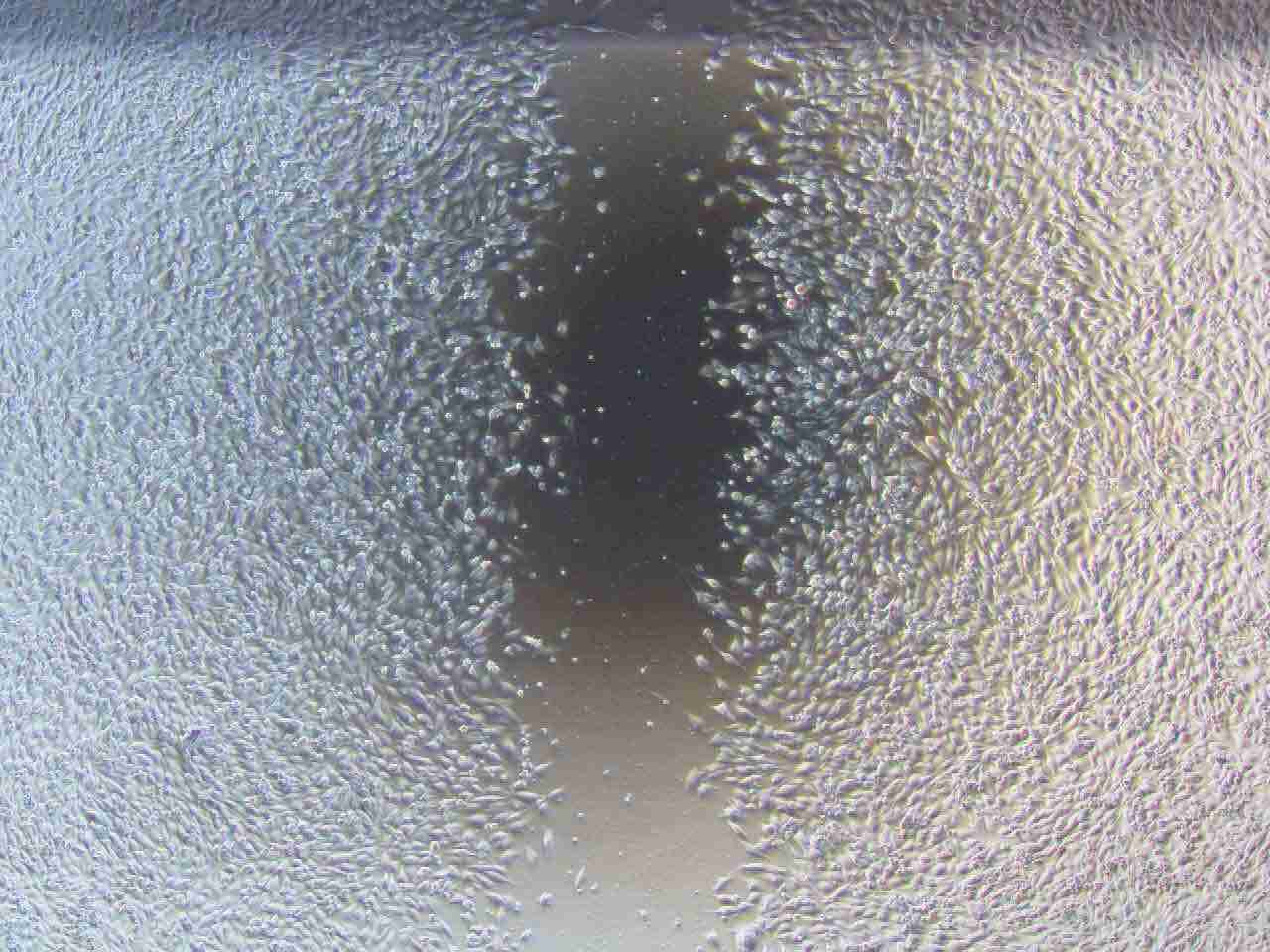

Supplement: Supplementary file 1 [file DataSheet1.ZIP › Raw data/wound healing piture & data-SPSS&GraphPad7 statistics/2.5-3.2.jpg]

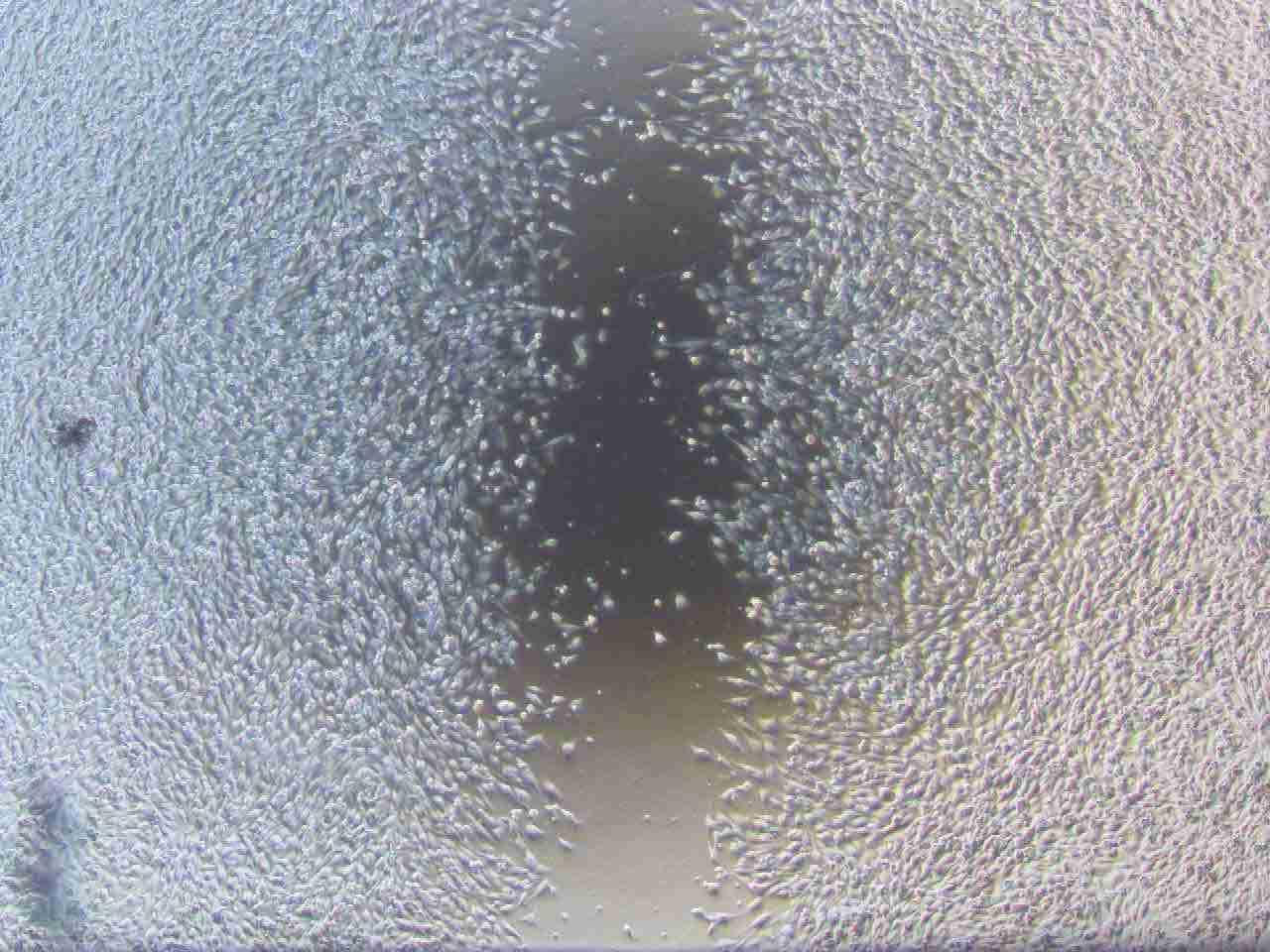

Supplement: Supplementary file 1 [file DataSheet1.ZIP › Raw data/wound healing piture & data-SPSS&GraphPad7 statistics/2.5-3.3.jpg]

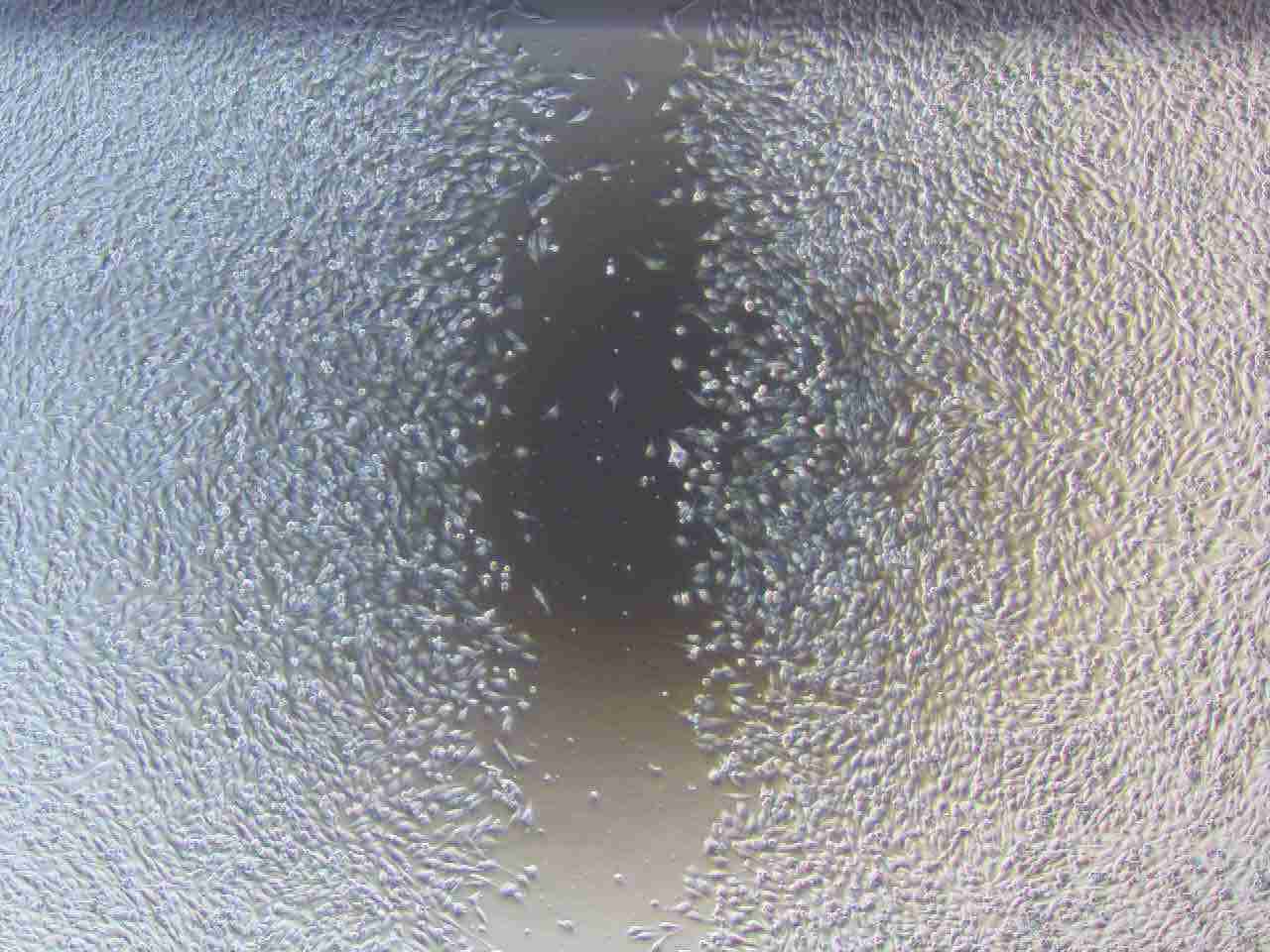

Supplement: Supplementary file 1 [file DataSheet1.ZIP › Raw data/wound healing piture & data-SPSS&GraphPad7 statistics/2.5-3.4.jpg]

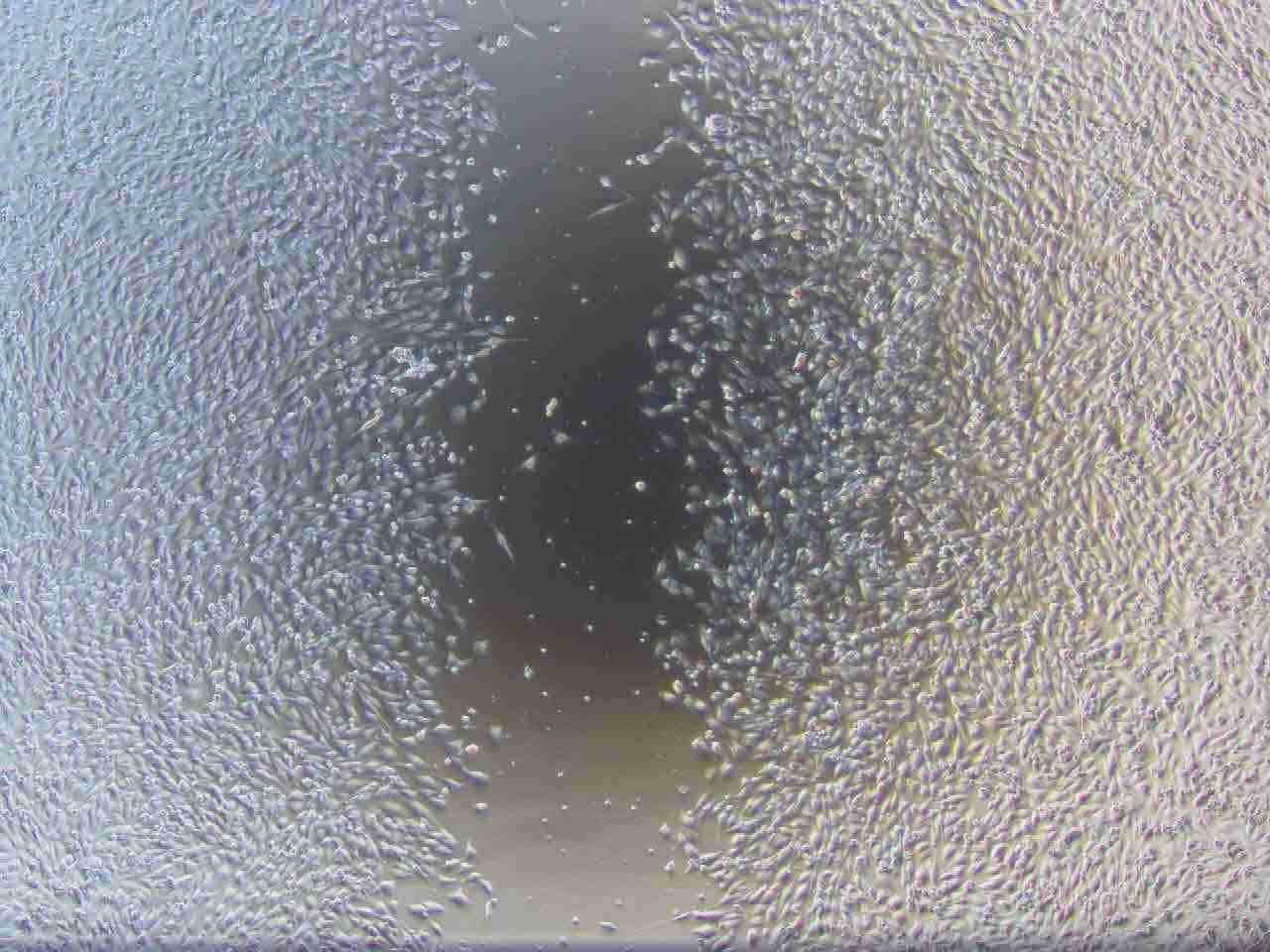

Supplement: Supplementary file 1 [file DataSheet1.ZIP › Raw data/wound healing piture & data-SPSS&GraphPad7 statistics/2.5-3.5.jpg]

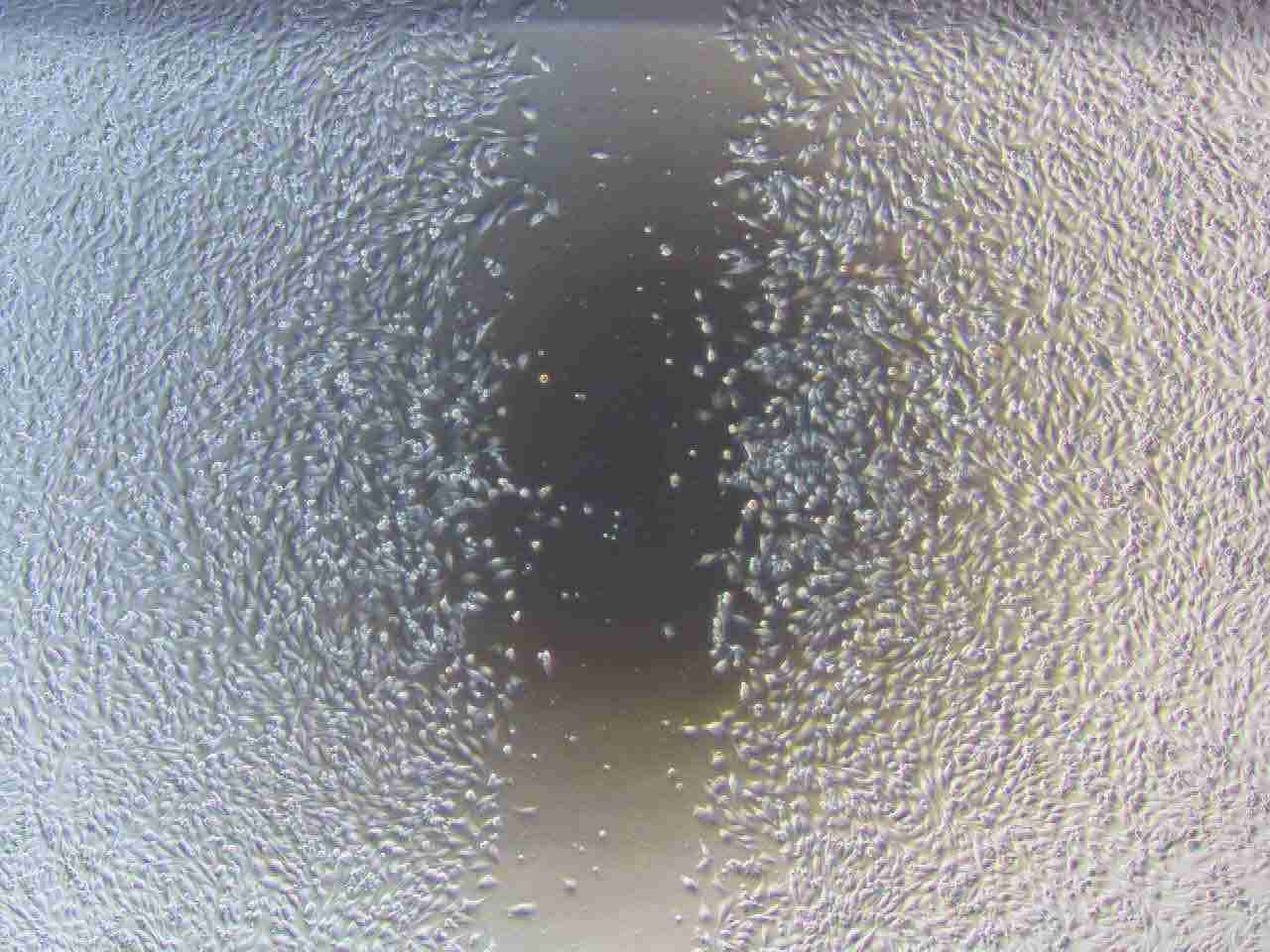

Supplement: Supplementary file 1 [file DataSheet1.ZIP › Raw data/wound healing piture & data-SPSS&GraphPad7 statistics/2.5-3.6.jpg]

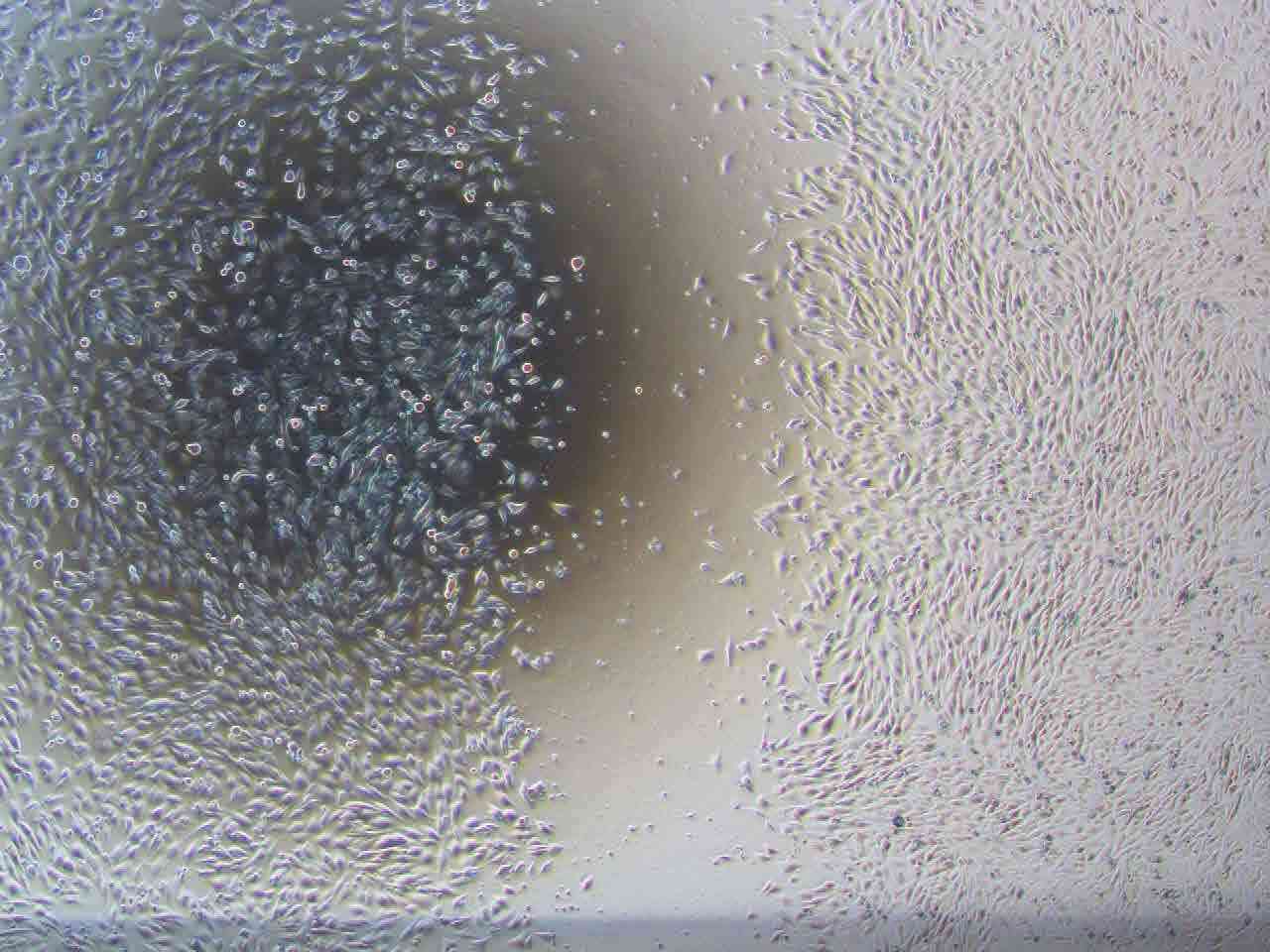

Supplement: Supplementary file 1 [file DataSheet1.ZIP › Raw data/wound healing piture & data-SPSS&GraphPad7 statistics/5-1.1.jpg]

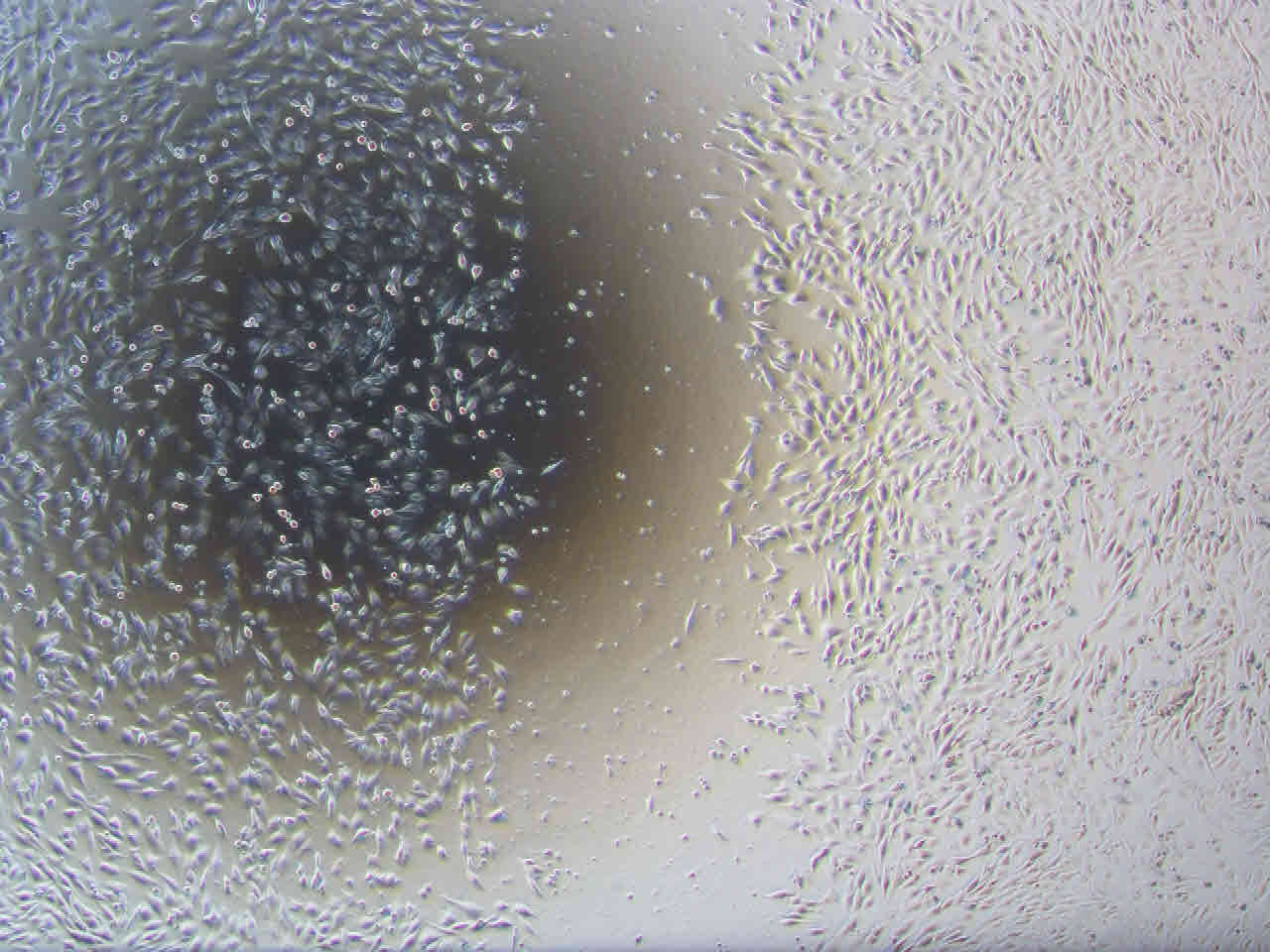

Supplement: Supplementary file 1 [file DataSheet1.ZIP › Raw data/wound healing piture & data-SPSS&GraphPad7 statistics/5-1.3.jpg]

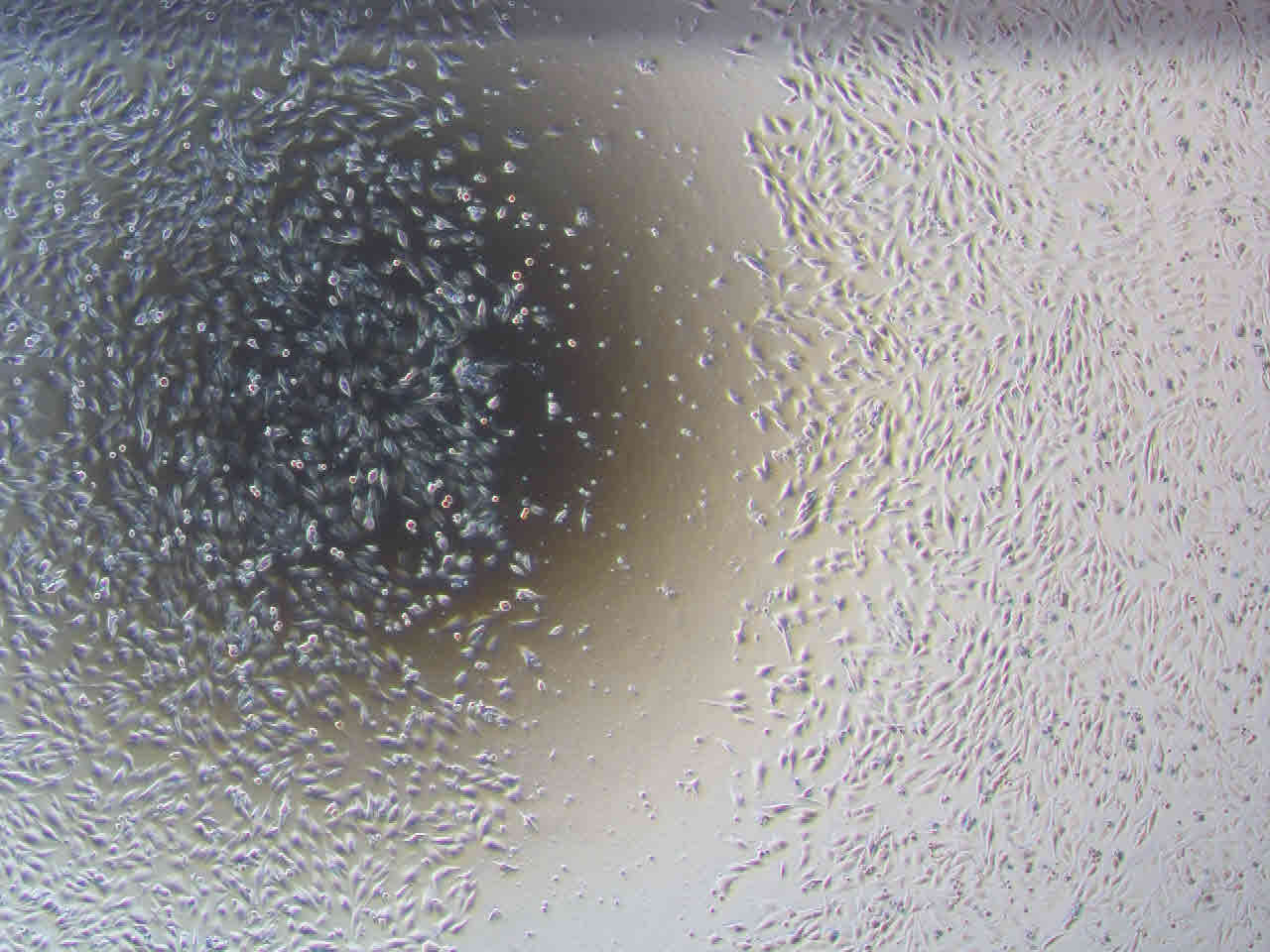

Supplement: Supplementary file 1 [file DataSheet1.ZIP › Raw data/wound healing piture & data-SPSS&GraphPad7 statistics/5-1.4.jpg]

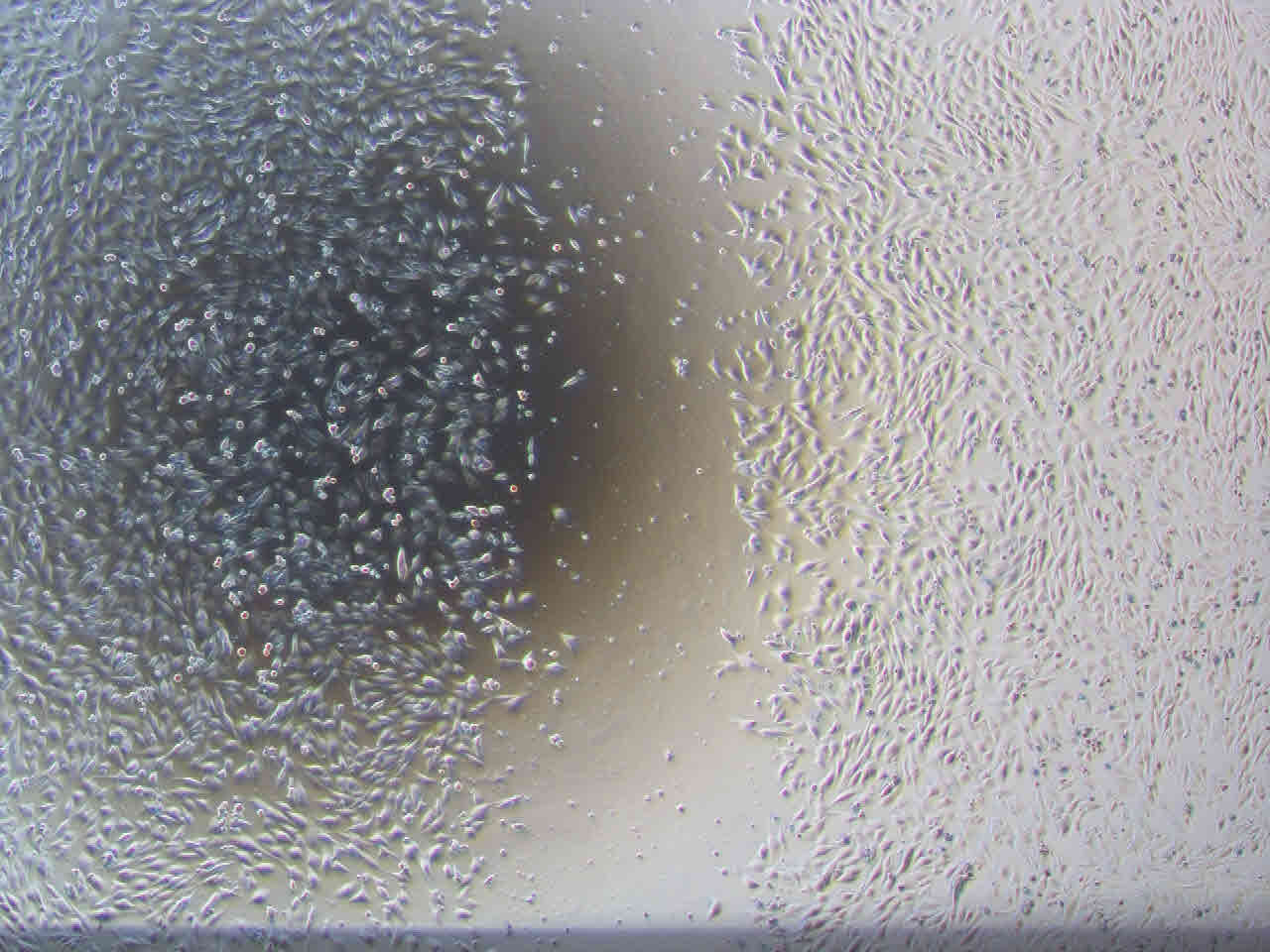

Supplement: Supplementary file 1 [file DataSheet1.ZIP › Raw data/wound healing piture & data-SPSS&GraphPad7 statistics/5-1.5.jpg]

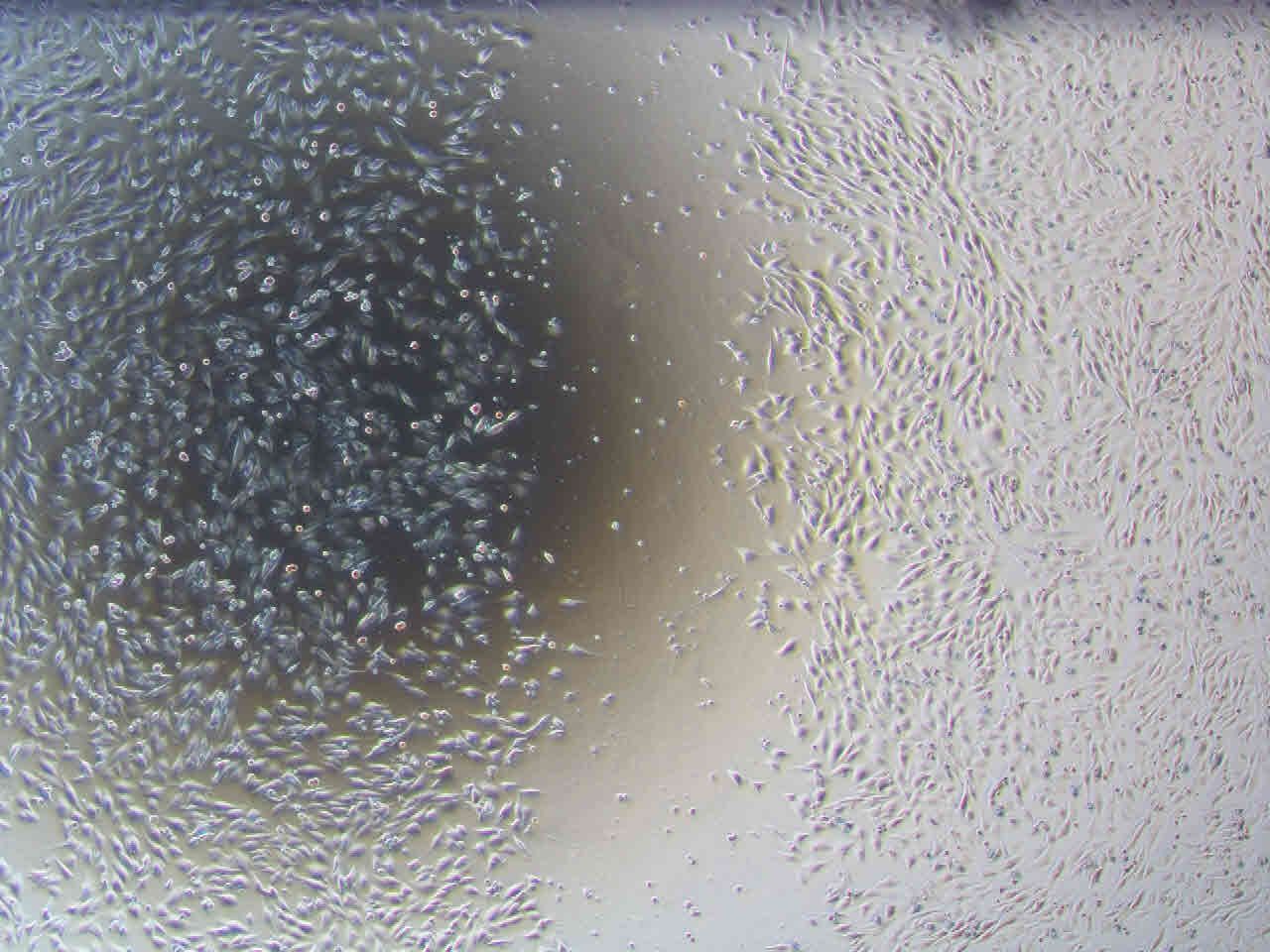

Supplement: Supplementary file 1 [file DataSheet1.ZIP › Raw data/wound healing piture & data-SPSS&GraphPad7 statistics/5-1.6.jpg]

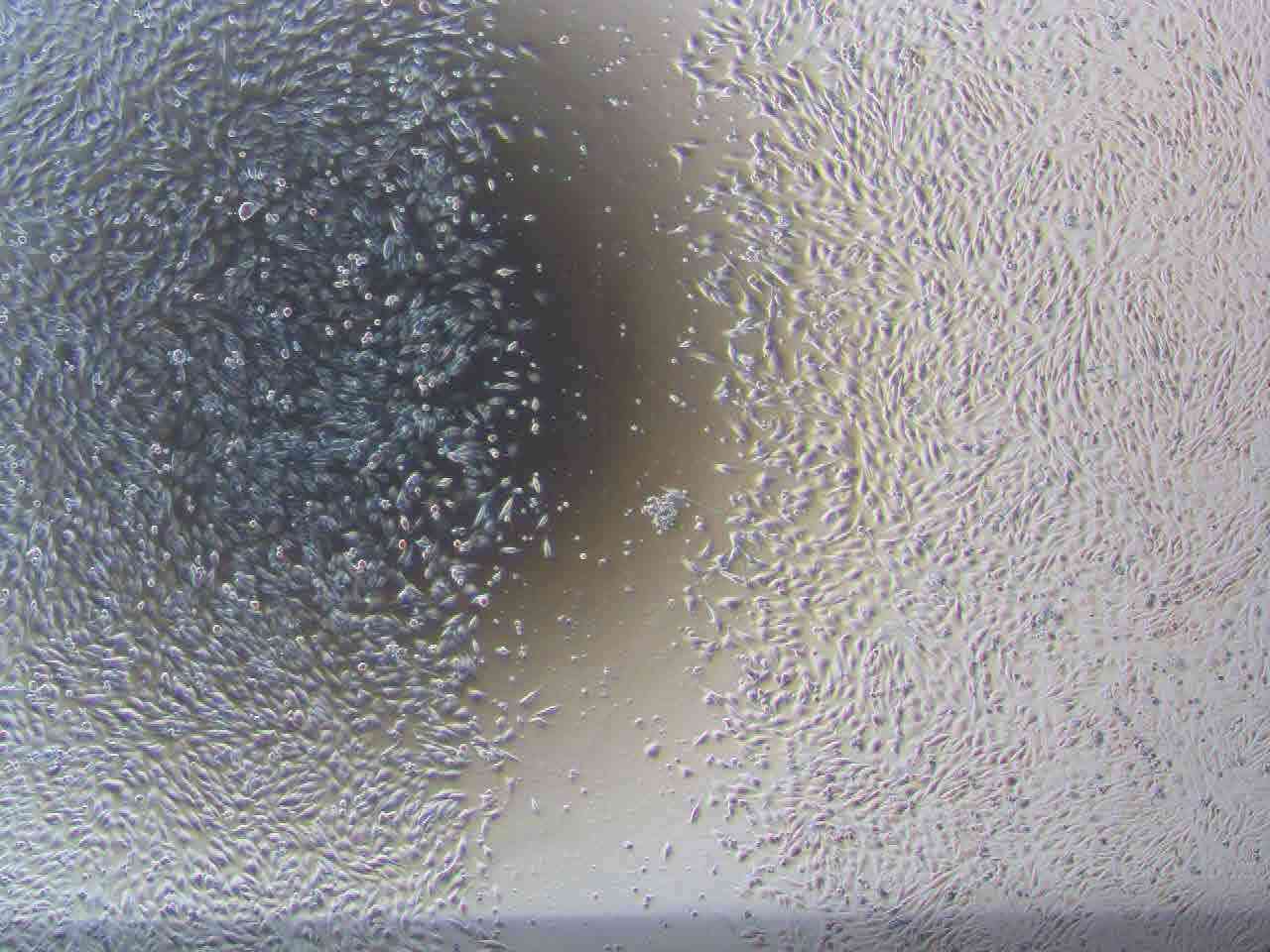

Supplement: Supplementary file 1 [file DataSheet1.ZIP › Raw data/wound healing piture & data-SPSS&GraphPad7 statistics/5-2.1.jpg]
